# Proceedings of the Canadian society of allergy and clinical immunology annual scientific meeting 2015

**DOI:** 10.1186/s13223-016-0118-0

**Published:** 2016-08-25

**Authors:** Marie-Ève Côté, Marie-Ève Boulay, Sophie Plante, Jamila Chakir, Louis-Philippe Boulet, Hanan Ahmed, Maria-Beatriz Ospina, Kyriaki Sideri, Harissios Vliagoftis, Sara F. Johnson, Roberta L. Woodgate, Guilhem Cros, Pierre Teira, Sonia Cellot, Henrique Bittencourt, Helene Decaluwe, Marie France Vachon, Michel Duval, Elie Haddad, Vy H. D. Kim, Anne Pham-Huy, Eyal Grunebaum, John-Paul Oliveria, Stephanie Phan, Mark W. Tenn, Damian Tworek, Steven G. Smith, Adrian J. Baatjes, Caitlin D. Obminski, Caroline E. Munoz, Tara X. Scime, Roma Sehmi, Gail M. Gauvreau, Brittany M. Salter, Steven G. Smith, Caitlin D. Obminski, Caroline E. Munoz, Abbey Schlatman, Tara X. Scime, Rick Watson, Roya Sherkat, Razieh Khoshnevisan, Saba Sheikhbahaei, Stephen Betschel, Richard Warrington, Robert Schellenberg, Michael N. Fein, Jean-Philippe Pelletier, Manstein Kan, Roxane Labrosse, Raymond Mak, James Loh, Amin Kanani, Dominik A. Nowak, Paul K. Keith, Daniel Pannozzo, Hermenio C. Lima, Diana Pham, Hoang Pham, Gonzalo G. Alvarez, Istvan T. Bencze, Krishna B. Sharma, Mark Smith, Shawn Aaron, Jennifer Block, Tara Keays, Judith Leech, David Schneidermen, Jodi Cameron, Jennifer Forgie, Alicia Ring, John W. O’Quinn, Stephanie Santucci, William H. Yang, Ena Gaudet, Shawn Aaron, Mathew R. Voisin, Rozita Borici-Mazi, Kateryna Vostretsova, Donald F. Stark, Elizabeth Yeboah, Michelle Martin-Rhee, Cheryl Gula, Clare Cheng, Geoff Paltser, Alizée Dery, Ann Clarke, Kari Nadeau, Laurie Harada, Kimberley Weatherall, Celia Greenwood, Denise Daley, Yuka Asai, Moshe Ben-Shoshan, Ling Ling, Maria B. Ospina, Jennifer L. P. Protudjer, Mirja Vetander, Marianne van Hage, Ola Olén, Magnus Wickman, Anna Bergström, Timothy Teoh, Christopher Mill, Tiffany Wong, Ingrid Baerg, Angela Alexander, Kyla J. Hildebrand, John Dean, Boris Kuzeljevic, Edmond S. Chan, Jonathan Argeny, Mia Gona-Hoepler, Petra Fucik, Edith Nachbaur, Saskia Gruber, Reto Crameri, Andreas Glaser, Zsolt Szépfalusi, Claudio Rhyner, Thomas Eiwegger, Greg Plunkett, Brad Mire, Mehtap Yazicioglu, Ceren Can, Gokce Ciplak, Victoria E. Cook, Elodie Portales-Casamar, Emil P. Nashi, Sofianne Gabrielli, Marie-Noel Primeau, Christine Lejtenyi, Elena Netchiporouk, Alizee Dery, Greg Shand, Erica Hoe, Joel Liem, Jason K. Ko, David J. T. Huang, Jorge A. Mazza, Mary McHenry, Anthony Otley, Wade Watson, John N. Kraft, Mihaela Paina, Ahmed A. Darwish Hassan, Delia Heroux, Lynn Crawford, Gail Gauvreau, Judah Denburg, Linda Pedder, Zave Chad, Gordon Sussman, Jacques Hébert, Charles Frankish, Timothy Olynych, Amarjit Cheema, Jaime Del Carpio, Rachel Harrison, Bahar Torabi, Elaine Medoff, Jennifer Mill, Jaclyn A. Quirt, Xia Wen, Jonathan Kim, Angel Jimenez Herrero, Harold L. Kim, Magdalena J. Grzyb, Marie-Noël Primeau, Meghan B. Azad, Zihang Lu, Allan B. Becker, Padmaja Subbarao, Piushkumar J. Mandhane, Stuart E. Turvey, Malcolm R. Sears, The CHILD Study Investigators, Anne-Marie Boucher-Lafleur, Valérie Gagné-Ouellet, Éric Jacques, Catherine Laprise, Michael Chen, Toby McGovern, Mikael Adner, James G. Martin, Nela Cosic, Henry Ntanda, Gerald Giesbrecht, Anita Kozyrskyj, Nicole Letourneau, Bassel Dawod, Jean Marshall, Sarah De Schryver, Michelle Halbrich, Sebastian La Vieille, Harley Eisman, Reza Alizadehfar, Lawrence Joseph, Judy Morris, Laura Y. Feldman, Jesse D. Thacher, Inger Kull, Erik Melén, Göran Pershagen, Jennifer L. P. Protudjer, Ali Hosseini, Tillie L. Hackett, Jeremy Hirota, Kelly McNagny, Susan Wilson, Chris Carlsten, Saiful Huq, Rishma Chooniedass, Brenda Gerwing, Henry Huang, Diana Lefebvre, Allan Becker, Mona M. Khamis, Hanan Awad, Kevin Allen, Darryl J. Adamko, Anas El-Aneed, Young Woong Kim, Daniel R. Gliddon, Casey P. Shannon, Amrit Singh, Pascal L. C. Hickey, Anne K. Ellis, Helen Neighbour, Mark Larche, Scott J. Tebbutt, Erika Ladouceur, Miriam Stewart, Josh Evans, Jeff Masuda, Teresa To, Malcolm King, Miriam Larouche, Liming Liang, Stephanie A. Legere, Ian D. Haidl, Jean-Francois Legaré, Jean S. Marshall, Malcolm Sears, Theo J. Moraes, Felix Ratjen, Per Gustafsson, Wendy Lou, Michelle L. North, Elizabeth Lee, Vanessa Omana, Jenny Thiele, Jeff Brook, Tanvir Rahman, Duncan Lejtenyi, Ryan Fiter, Ciriaco Piccirillo, Bruce Mazer, Elinor Simons, Kyla Hildebrand, Stuart Turvey, Mari DeMarco, Kim-Anh Le Cao, Gail M. Gauvreau, J. Mark FitzGerald, Paul M. O’Byrne, Leah T. Stiemsma, Marie-Claire Arrieta, Jasmine Cheng, Pedro A. Dimitriu, Lisa Thorson, Sophie Yurist, Diana L. Lefebvre, Piush Mandhane, Kelly M. McNagny, Tobias Kollmann, William W. Mohn, B. Brett Finlay, Maxwell M. Tran, Diana L. Lefebvre, Chinthanie F. Ramasundarahettige, Wei Hao Dai, Piush J. Mandhane, Damian Tworek, Seamus N. O’Byrne, Paul M. O’Byrne, Judah A. Denburg, Laura Walsh, Mena Soliman, Lisa M. Steacy, Daniel E. Adams, Linda Warner, Mary Ann Mauro, Robby Mamonluk, ChenXi Yang, Ed M. Conway

**Affiliations:** 1 Centre de Recherche de l’Institut, Universitaire de Pneumologie et de Cardiologie de Québec, Quebec, QC G1V 4G5 Canada; 2 Department of Medicine, University of Alberta, Edmonton, AB T6G2B7 Canada; 3 Alberta Health Services, Respiratory Health Strategic Clinical Network, Edmonton, AB T6G2B7 Canada; 4 College of Nursing, University of Manitoba, Winnipeg, MB R3T 2N2 Canada; 5 Department of Pediatric Hematology-Oncology, CHU Sainte Justine, University of Montreal, Montreal, QC Canada; 6 Department of Pediatric Immunology, University of Montreal, Montreal, QC Canada; 7 Division of Immunology/Allergy, Department of Pediatrics, Hospital for Sick Children, Toronto, ON Canada; 8 Division of Pediatric Infectious Diseases, Children’s Hospital of Eastern Ontario, Ottawa, ON Canada; 9 Department of Medicine, McMaster University, Hamilton, ON Canada; 10 Acquired Immunodeficiency Research Center, Isfahan University of Medical Sciences, Isfahan, Iran; 11 Division of Allergy and Clinical Immunology, University of Toronto, Toronto, ON Canada; 12 Division of Allergy and Clinical Immunology, University of Manitoba, Winnipeg, MB Canada; 13 Division of Allergy and Immunology, University of British Columbia, Vancouver, BC Canada; 14 Department of Clinical Immunology and Allergy, McGill University, Montreal, QC H3G 1A4 Canada; 15 Department of Cardiology, McGill University, Montreal, QC H3G 1A4 Canada; 16 Division of Allergy and Immunology, Department of Medicine, St Paul’s Hospital, University of British Columbia, Vancouver, BC V6Z 1Y6 Canada; 17 Department of Pediatric Immunology, CHU Sainte Justine, University of Montreal, Montreal, QC Canada; 18 Department of Pediatric Hematology-Oncology, University of Montreal, Montreal, QC Canada; 19 Internal Medicine, University of British Columbia, Vancouver, BC V5Z 1M9 Canada; 20 Division of Clinical Immunology and Allergy, University of British Columbia, Vancouver, BC V5Z 1M9 Canada; 21 School of Medicine, McMaster University, Hamilton, ON L8S 4L8 Canada; 22 Division of Clinical Immunology and Allergy, Department of Medicine, McMaster University, Hamilton, ON L8S 4L8 Canada; 23 Division of Dermatology, Department of Medicine, McMaster University, Hamilton, ON L8S 4L8 Canada; 24 Ottawa Allergy Research Corporation, Ottawa, ON Canada; 25 Division of Respiratory Medicine, The Ottawa Hospital, Ottawa, ON Canada; 26 Faculty of Medicine, University of Ottawa Medical School, Ottawa, ON Canada; 27 Division of Internal Medicine, Montfort Hospital, Ottawa, ON Canada; 28 Division of Respirology, The Ottawa Hospital, Ottawa, ON Canada; 29 University of Ottawa, Faculty of Medicine, Ottawa, ON Canada; 30 Faculty of Health Sciences, School of Medicine, Queen’s University, Kingston, ON K7L 3N6 Canada; 31 Division of Allergy and Immunology, Department of Medicine, Queen’s University, 166 Brock Street, Kingston, ON K7L 5G2 Canada; 32 Internal Medicine, University of British Columbia, Vancouver, BC V5R 3V8 Canada; 33 Allergy and Immunology, University of British Columbia, Vancouver, BC V5Z 1M9 Canada; 34 Division of Clinical Immunology and Allergy, Department of Medicine, McMaster University, Hamilton, ON L8S 4K1 Canada; 35 Canadian Institute for Health Information, Toronto, ON M2P 2B7 Canada; 36 Division of Clinical Epidemiology, Department of Medicine, McGill University Health Center, Montreal, QC Canada; 37 Division of Rheumatology, Department of Medicine, University of Calgary, Calgary, AB Canada; 38 Division of Allergy, Immunology, and Rheumatology, Department of Pediatrics, Stanford University School of Medicine, Palo Alto, CA USA; 39 Anaphylaxis Canada, Toronto, ON Canada; 40 Multiple Births Canada, Orleans, ON Canada; 41 Department of Medicine, University of British Columbia, Vancouver, BC Canada; 42 Division of Dermatology, Department of Medicine, Queen’s University, Kingston, ON Canada; 43 Division of Allergy and Clinical Immunology, Department of Pediatrics, McGill University, Montreal, QC Canada; 44 Lady Davis Institute, Jewish General Hospital, Montreal, QC Canada; 45 Departments of Oncology, Epidemiology, Biostatistics and Occupational Health, and Human Genetics, McGill University, Montreal, QC Canada; 46 Department of Medicine, University of Alberta, Edmonton, AB T6G 2S2 Canada; 47 Alberta Health Services, Respiratory Health Strategic Clinical Network, Edmonton, AB T6G 2G3 Canada; 48 Centre for Allergy Research, Karolinska Institutet, Stockholm, Sweden; 49 Institute for Environmental Medicine, Karolinska Institutet, Stockholm, Sweden; 50 Sachs’ Children and Youth Hospital, Södersjukhuset, Stockholm, Sweden; 51 Clinical Immunology and Allergy Unit, Department of Medicine Solna, Karolinska Institutet and University Hospital, Stockholm, Sweden; 52 Clinical Epidemiology, Karolinska Institutet, Stockholm, Sweden; 53 Faculty of Medicine, University of British Columbia, Vancouver, BC V6T 1Z3 Canada; 54 Division of Allergy and Immunology, Department of Pediatrics, BC Children’s Hospital, University of British Columbia, Vancouver, BC V6H 3V4 Canada; 55 Child and Family Research Institute, Vancouver, BC V5Z 4H4 Canada; 56 Department of Paediatrics and Adolescent Medicine, Medical University of Vienna, Vienna, Austria; 57 Swiss Institute of Asthma and Allergy Research, University of Zurich, Davos, Switzerland; 58 ALK, Round Rock, TX 78664 USA; 59 Department of Pediatric Allergy, Trakya University, 22030 Edirne, Turkey; 60 Department of Pediatrics, Trakya University, 22030 Edirne, Turkey; 61 Department of Pediatrics, Division of Allergy and Immunology, University of British Columbia, Vancouver, Canada; 62 Division of Pediatric Allergy and Clinical Immunology, Department of Pediatrics, McGill University Health Center, Montreal, QC Canada; 63 Division of Clinical Epidemiology, Department of Medicine, McGill University Healthy Center, Montreal, QC Canada; 64 Schulich School of Medicine and Dentistry, Western University, Windsor, ON N9B 3P4 Canada; 65 Windsor Allergy Asthma Education Centre, Windsor, N8X 2G1 Canada; 66 Schulich School of Medicine and Dentistry, University of Western Ontario, London, ON N6A 5C1 Canada; 67 Division of Allergy and Clinical Immunology, Department of Medicine, University of Western Ontario, London, ON N6A 3K6 Canada; 68 Department of Pediatrics, Faculty of Medicine, Dalhousie University, Halifax, NS Canada; 69 Division of Allergy, IWK Health Centre, Halifax, NS Canada; 70 Division of Gastroenterology, IWK Health Centre, Halifax, NS Canada; 71 Lynde Institute for Dermatology, Markham, ON L3P 1X2 Canada; 72 Department of Pediatrics, McMaster University, Hamilton, ON L8S 4K1 Canada; 73 Department of Medicine, McMaster University, Hamilton, ON L8N 3Z5 Canada; 74 Department of Pediatrics, University of Ottawa, Ottawa, ON Canada; 75 University of Toronto, Toronto, ON Canada; 76 Gordon Sussman Clinical Research, Toronto, ON Canada; 77 Centre de Recherche Appliquée en Allergie de Québec, Quebec, QC Canada; 78 Trillium Health Partners, Mississauga, ON Canada; 79 McGill University, Montreal, QC Canada; 80 Pediatric Allergy and Immunology, Montreal Children’s Hospital, McGill University, Montreal, QC H4A 3J1 Canada; 81 McGill University, Montreal, QC H3A 0G4 Canada; 82 Division of Clinical Immunology and Allergy, Department of Medicine, Western University, London, ON N6A 4V2 Canada; 83 Pediatric Allergy and Clinical Immunology, Department of Pediatrics, McGill University, Montreal, QC H4A 3J1 Canada; 84 Department of Pediatrics and Child Health, University of Manitoba and Children’s Hospital Research Institute of Manitoba, Winnipeg, MB Canada; 85 Department of Pediatrics, Hospital for Sick Children, University of Toronto, Toronto, ON Canada; 86 Department of Pediatrics, University of Alberta, Edmonton, AB Canada; 87 Department of Pediatrics, Child and Family Research Institute and British Columbia Children’s Hospital, University of British Columbia, Vancouver, BC Canada; 88 Canadian Healthy Infant Longitudinal Development Study, Hamilton, Canada; 89 Département des Sciences Fondamentales, Université du Québec à Chicoutimi, Chicoutimi, QC Canada; 90 Centre de recherche, Institut Universitaire de Cardiologie et de Pneumologie, Ste-Foy, QC Canada; 91 Research Institute of the McGill University Health Centre, Department of Medicine, McGill University, Montreal, QC Canada; 92 Institute of Environmental Medicine, Karolinska Institutet, Stockholm, Sweden; 93 Faculty of Nursing, Department of Pediatrics and Psychiatry, University of Calgary, Calgary, AB Canada; 94 Faculty of Medicine, Department of Pediatrics and Psychiatry, Cumming School of Medicine, University of Calgary, Calgary, AB Canada; 95 Faculty of Medicine, Department of Pediatrics, Cumming School of Medicine, University of Calgary, Calgary, AB Canada; 96 Faculty of Medicine and Dentistry, Department of Pediatrics, University of Alberta and School of Public Health, University of Alberta, Edmonton, AB Canada; 97 Department of Pathology, Dalhousie University, Halifax, NS Canada; 98 Department of Microbiology and Immunology, Dalhousie University, Halifax, NS Canada; 99 Division of Allergy and Clinical Immunology, Department of Pediatrics, Montréal Children’s Hospital, Montreal, QC Canada; 100 Division of Paediatric Allergy and Clinical Immunology, Department of Paediatrics, University of Manitoba, Winnipeg, MB Canada; 101 Food Directorate, Health Canada, Ottawa, ON Canada; 102 Department of Emergency Medicine, Department of Pediatrics, Montréal Children’s Hospital, Montreal, QC Canada; 103 Department of Epidemiology and Biostatistics, McGill University, Montreal, QC Canada; 104 Department of Emergency Medicine, Hôpital du Sacré-Cœur, Montreal, QC Canada; 105 Dalla Lana School of Public Health, University of Toronto, Toronto, ON Canada; 106 Child Health Evaluative Sciences, The Hospital for Sick Children, Toronto, ON Canada; 107 Centre for Allergy Research, Karolinska Institutet, Stockholm, Sweden; 108 Department of Medicine, Vancouver General Hospital, University of British Columbia, Vancouver, BC Canada; 109 Institute for Heart Lung Health, Centre for Heart Lung Innovation, St. Paul’s Hospital, University of British Columbia, Vancouver, BC Canada; 110 Biomedical Research Centre, University of British Columbia, Vancouver, BC Canada; 111 Histochemistry Research Unit, University of Southampton, Southampton, Hampshire UK; 112 Department of Pediatrics and Child Health, University of Manitoba, Winnipeg, MB Canada; 113 College of Pharmacy and Nutrition, University of Saskatchewan, Saskatoon, SK Canada; 114 College of Medicine, Department of Pediatrics, University of Saskatchewan, Saskatoon, SK Canada; 115 Experimental Medicine, University of British Columbia, Vancouver, BC Canada; 116 James Hogg Centre for Heart Lung Innovation, St. Paul’s Hospital, Vancouver, BC Canada; 117 Prevention of Organ Failure (PROOF) Centre of Excellence, Vancouver, BC Canada; 118 Circassia Ltd., Oxford, UK; 119 Adiga Life Sciences, Hamilton, ON Canada; 120 Departments of Medicine and Biomedical and Molecular Science, Queen’s University, Kingston, ON Canada; 121 Allergy Research Unit, Kingston General Hospital, Kingston, ON Canada; 122 Firestone Institute for Respiratory Health, McMaster University, Hamilton, ON Canada; 123 Faculty of Nursing, University of Alberta, Edmonton, AB Canada; 124 Faculty of Humanities and Social Sciences, Athabasca University, Athabasca, AB Canada; 125 School of Kinesiology and Health Studies, Queen’s University, Kingston, ON Canada; 126 Faculty of Nursing, University of Calgary, Calgary, AB Canada; 127 University of Toronto, Toronto, ON Canada; 128 Institute for Clinical Evaluative Sciences, North York, ON Canada; 129 Division of Pulmonary Medicine, University of Alberta, Edmonton, AB Canada; 130 Departments of Epidemiology and Biostatistics, Harvard T. H. Chan School of Public Health, Boston, MA USA; 131 Department of Microbiology and Immunology, Dalhousie University, Halifax, NS Canada; 132 Department of Surgery, Dalhousie University, Halifax, NS Canada; 133 Division of Respiratory Medicine, Department of Pediatrics, and Program in Physiology and Experimental Medicine, SickKids Research Institute, The Hospital for Sick Children and University of Toronto, Toronto, Canada; 134 Department of Medicine, McMaster University, Hamilton, Canada; 135 Department of Pediatrics, Central Hospital, Skövde, Sweden; 136 Dalla Lana School of Public Health, University of Toronto, Toronto, Canada; 137 Department of Biomedical and Molecular Sciences, Queen’s University, Kingston, ON Canada; 138 Division of Allergy and Immunology, Department of Medicine, Queen’s University, Kingston, ON Canada; 139 Air Quality Research Division, Environment Canada, Toronto, ON Canada; 140 Meakins-Christie Laboratories, McGill University, Montreal, QC Canada; 141 Division of Pediatric Allergy and Clinical Immunology, Montréal Children’s Hospital, Quebec, Canada; 142 Division of Allergy and Clinical Immunology, Montréal General Hospital, Montreal, QC Canada; 143 Section of Allergy and Immunology, Department of Pediatrics and Child Health, University of Manitoba, Winnipeg, MB Canada; 144 Division of Allergy and Immunology, Department of Pediatrics, British Columbia Children’s Hospital, Vancouver, BC Canada; 145 Department of Pediatrics, Hospital for Sick Children, Toronto, ON Canada; 146 Division of Respirology, Department of Medicine, McMaster University, Hamilton, ON Canada; 147 Department of Medicine, University of British Columbia, Vancouver, BC Canada; 148 Translational Research Institute, University of Queensland, Brisbane, Australia; 149 Centre de Pneumologie de L’Hopital, Université Laval, Quebec, QC Canada; 150 Department of Microbiology and Immunology, University of British Columbia, Vancouver, BC Canada; 151 Michael Smith Laboratories, University of British Columbia, Vancouver, BC Canada; 152 St. Joseph’s Healthcare, Hamilton, ON Canada; 153 Department of Pediatrics, University of Toronto, Toronto, ON Canada; 154 Hospital for Sick Children, Toronto, ON Canada; 155 Department of Pediatrics, University of Alberta, Edmonton, AB Canada; 156 School of Public Health, University of Alberta, Edmonton, AB Canada; 157 The Biomedical Research Centre, University of British Columbia, Vancouver, BC Canada; 158 Department of Pediatrics, University of British Columbia, Vancouver, BC Canada; 159 Department of Biochemistry and Molecular Biology, University of British Columbia, Vancouver, BC Canada; 160 Department of Paediatrics, University of Toronto, Toronto, ON Canada; 161 Department of Pediatrics, University of British Columbia, Vancouver, BC Canada; 162 Centre for Heart and Lung Innovation, University of British Columbia, Vancouver, BC Canada; 163 Centre for Blood Research, University of British Columbia, Vancouver, BC Canada

## Abstract

A1 Role of fibrocytes in allergic rhinitis

Marie-Ève Côté, Marie-Ève Boulay, Sophie Plante, Jamila Chakir, Louis-Philippe Boulet

A2 Patterns of aeroallergens sensitization in Northern Alberta

Hanan Ahmed, Maria-Beatriz Ospina, Kyriaki Sideri, Harissios Vliagoftis

A3 Addressing acceptable risk for adolescents with Food-Induced Anaphylaxis (FIA)

Sara F. Johnson, Roberta L. Woodgate

A4 Outcomes of matched related and unrelated bone marrow transplantation after reduced-toxicity conditioning for children suffering from Chronic Granulomatous Disease

Guilhem Cros, Pierre Teira, Sonia Cellot, Henrique Bittencourt, Helene Decaluwe, Marie France Vachon, Michel Duval, Elie Haddad

A5 Outcomes of patients with severe combined immunodeficiency (SCID) prior to and after initiation of newborn screening for SCID in Ontario

Vy H.D. Kim, Anne Pham-Huy, Eyal Grunebaum

A6 Detection of regulatory B cells in the airways of subjects with asthma

John-Paul Oliveria, Stephanie Phan, Mark W. Tenn, Damian Tworek, Steven G. Smith, Adrian J. Baatjes, Caitlin D. Obminski, Caroline E. Munoz, Tara X. Scime, Roma Sehmi, Gail M Gauvreau

A7 Characterization of IgE-expressing B cells in the airways and peripheral blood of allergic asthmatic subjects

John-Paul Oliveria, Stephanie Phan, Mark W. Tenn, Brittany M Salter, Steven G Smith, Caitlin D Obminski, Caroline E Munoz, Abbey Schlatman, Tara X Scime, Rick Watson, Roma Sehmi, Gail M Gauvreau

A8 Pregnancy: could it be a risk factor for primary immunodeficient patients

Roya Sherkat, Razieh Khoshnevisan, Saba Sheikhbahaei

A9 Clinical experience with Octagam: a Canadian retrospective chart review

Stephen Betschel, Richard Warrington, Robert Schellenberg

A10 Kounis syndrome secondary to contrast media with inferior ST elevations and bilateral ischemic stroke

Michael N Fein, Jean-Philippe Pelletier

A11 Honey bee venom immunotherapy ineffective in bumble bee-induced anaphylaxis: case report and review of literature

Manstein Kan, Robert Schellenberg

A12 Delayed immune reconstitution occurring after multiple immune complications of hematological stem cell transplantation for a leaky SCID

Roxane Labrosse, Guilhem Cros, Pierre Teira, Henrique Bittencourt, Helene Decaluwe, Michel Duval, Elie Haddad

A13 Comparison of Three Case Reports of Acquired Angioedema: presentation, management and outcome

Raymond Mak, James Loh, Amin Kanani

A14 Sitagliptin-associated angioedema not related to concurrent use of ARB or ACE inhibitor

Dominik A. Nowak, Paul K. Keith

A15 Sneddon-Wilkinson subcorneal pustular dermatosis associated with an IgA monoclonal gammopathy

Daniel Pannozzo, Dominik A. Nowak, Hermenio C. Lima

A16 Omalizumab can be effective in patients with allergic bronchopulmonary aspergillosis

Diana Pham, Hoang Pham, Gonzalo G. Alvarez, Istvan T. Bencze, Krishna B. Sharma, Mark Smith, Shawn Aaron, Jennifer Block, Tara Keays, Judith Leech, David Schneidermen, Jodi Cameron, Jennifer Forgie, Alicia Ring, John W. O’Quinn, Stephanie Santucci, William H. Yang

A17 Efficacious use of omalizumab in the treatment of cystic fibrosis

Diana Pham, Hoang Pham, Ena Gaudet, Shawn Aaron, Stephanie Santucci, William H. Yang

A18 HAE with normal C1-INH with inconsistent response to C1 esterase inhibitor infusion but reliably responsive to icatibant

Hoang Pham, Stephanie Santucci, William H. Yang

A19 Anaphylaxis reaction to lactase enzyme

Mathew R. Voisin, Rozita Borici-Mazi

A20 Risk of solid tumor malignancies in patients with primary immune deficiency

Kateryna Vostretsova, Donald F. Stark

A21 Is it time to adopt the chromogenic assay for measuring C1 esterase inhibitor function in patients with HAE Type 2?

Elizabeth Yeboah, Paul K. Keith

A22 Emergency department visits for anaphylaxis and allergic reactions

Michelle Martin-Rhee, Cheryl Gula, Clare Cheng, Geoff Paltser

A23 START: Susceptibility To food Allergies in a Registry of Twins

Alizée Dery, Ann Clarke, Kari Nadeau, Laurie Harada, Kimberley Weatherall, Celia Greenwood, Denise Daley, Yuka Asai, Moshe Ben-Shoshan

A24 Qualifying the diagnostic approach employed by allergists when managing patients with self-diagnosed non-celiac gluten sensitivity (NCGS)

Lee Horgan, Teresa Pun

A25 Retrospective analysis on the agreement between skin prick test and serum food specific IgE antibody in adults with suspected food allergy

Ling Ling, Maria B. Ospina, Kyriaki Sideri, Harissios Vliagoftis

A26 Staple food hypersensitivity from infancy to adolescence: a report from the BAMSE cohort

Jennifer L.P. Protudjer, Mirja Vetander, Marianne van Hage, Ola Olén, Magnus Wickman, Anna Bergström

A27 Evaluating the impact of supervised epinephrine autoinjector administration during food challenges on perceived parent confidence

Timothy Teoh, Christopher Mill, Tiffany Wong, Ingrid Baerg, Angela Alexander, Kyla J. Hildebrand, John Dean, Boris Kuzeljevic, Edmond S. Chan

A28 Local immunoglobulin production to *Aspergillus fumigatus* cystic fibrosis

Jonathan Argeny, Mia Gona-Hoepler, Petra Fucik, Edith Nachbaur, Saskia Gruber, Reto Crameri, Andreas Glaser, Zsolt Szépfalusi, Claudio Rhyner, Thomas Eiwegger

A29 Extract consumption with skin prick test (SPT) devices

Greg. Plunkett, Brad Mire

A30 Evaluation of our cases with nonsteroidal anti-inflammatory drug reactions

Mehtap Yazicioglu, Ceren Can, Gokce Ciplak

A31 Reasons for referral and final diagnoses in a tertiary care pediatric allergy clinic

Victoria E. Cook, Kyla J. Hildebrand, Elodie Portales-Casamar, Christopher Mill, Edmond S. Chan

A32 Internist referral practices for inpatients with self-reported penicillin allergies at a tertiary care teaching hospital

Michael N Fein, Emil P Nashi

A33 Assessing the risk of reactions in children with a negative oral challenge after a subsequent use of amoxicillin

Sofianne Gabrielli, Christopher Mill, Marie-Noel Primeau, Christine Lejtenyi, Elena Netchiporouk, Alizee Dery, Greg Shand, Moshe Ben-Shoshan

A34 Validity of self-reported penicillin allergies

Erica Hoe, Joel Liem

A35 Effectiveness of allergy-test directed elimination diets in eosinophilic esophagitis

Jason K. Ko, David J.T. Huang, Jorge A. Mazza

A36 Allergy testing and dietary management in pediatric eosinophilic esophagitis (EoE): A retrospective review of a tertiary Canadian centre’s experience

Mary McHenry, Anthony Otley,Wade Watson

A37 Visualizing the impact of atopic and allergic skin disease

Dominik A. Nowak, John N. Kraft

A38 Cystic fibrosis with and without nasal polyposis in pediatric patients: a cross-sectional comparative study

Mihaela Paina, Ahmed A. Darwish Hassan, Delia Heroux, Lynn Crawford, Gail Gauvreau, Judah Denburg, Linda Pedder, Paul K. Keith

A39 Evaluation of macrolide antibiotic hypersensitivity: the role of oral challenges in children

Bahar Torabi, Marie-Noel Primeau, Christine Lejtenyi, Elaine Medoff, Jennifer Mill, Moshe Ben-Shoshan

A40 Venom allergy testing: is a graded approach necessary?

Jaclyn A. Quirt, Xia Wen, Jonathan Kim, Angel Jimenez Herrero, Harold L. Kim

A41 The role of oral challenges in evaluating cephalosporin hypersensitivity reactions in children

Magdalena J. Grzyb, Marie-Noël Primeau, Christine Lejtenyi, Elaine Medoff, Jennifer Mill, Moshe Ben-Shoshan

A42 Breastfeeding and infant wheeze, atopy and atopic dermatitis: findings from the Canadian Healthy Infant Longitudinal Development Study

Meghan B. Azad, Zihang Lu, Allan B. Becker, Padmaja Subbarao, Piushkumar J. Mandhane, Stuart E. Turvey, Malcolm R. Sears, the CHILD Study Investigators

A43 *IL33* DNA methylation in bronchial epithelial cells is associated to asthma

Anne-Marie Boucher-Lafleur, Valérie Gagné-Ouellet, Éric Jacques, Sophie Plante, Jamila Chakir, Catherine Laprise

A44 NRF2 mediates the antioxidant response to organic dust-induced oxidative stress in bronchial epithelial cells

Michael Chen, Toby McGovern, Mikael Adner, James G. Martin

A45 The effects of perinatal distress, immune biomarkers and mother-infant interaction quality on childhood atopic dermatitis (rash) at 18 months

Nela Cosic, Henry Ntanda, Gerald Giesbrecht, Anita Kozyrskyj, Nicole Letourneau

A46 Examining the immunological mechanisms associated with cow’s milk allergy

Bassel Dawod, Jean Marshall

A47 Tryptase levels in children presenting with anaphylaxis to the Montréal Children’s Hospital

Sarah De Schryver, Michelle Halbrich, Ann Clarke, Sebastian La Vieille, Harley Eisman, Reza Alizadehfar, Lawrence Joseph, Judy Morris, Moshe Ben-Shoshan

A48 Secondhand tobacco smoke exposure in infancy and the development of food hypersensitivity from childhood to adolescence

Laura Y. Feldman, Jesse D. Thacher, Inger Kull, Erik Melén, Göran Pershagen, Magnus Wickman, Jennifer L. P. Protudjer, Anna Bergström

A49 Combined exposure to diesel exhaust and allergen enhances allergic inflammation in the bronchial submucosa of atopic subjects

Ali Hosseini, Tillie L. Hackett, Jeremy Hirota, Kelly McNagny, Susan Wilson, Chris Carlsten

A50 Comparison of skin-prick test measurements by an automated system against the manual method

Saiful Huq, Rishma Chooniedass, Brenda Gerwing, Henry Huang, Diana Lefebvre, Allan Becker

A51 The accurate identification and quantification of urinary biomarkers of asthma and COPD through the use of novel DIL- LC-MS/MS methods

Mona M. Khamis, Hanan Awad, Kevin Allen, Darryl J. Adamko, Anas El-Aneed

A52 Systemic immune pathways associated with the mechanism of Cat-Synthetic Peptide Immuno-Regulatory Epitopes, a novel immunotherapy, in whole blood of cat-allergic people

Young Woong Kim, Daniel R. Gliddon, Casey P. Shannon, Amrit Singh, Pascal L. C. Hickey, Anne K. Ellis, Helen Neighbour, Mark Larche, Scott J. Tebbutt

A53 Reducing the health disparities: online support for children with asthma and allergies from low-income families

Erika Ladouceur, Miriam Stewart, Josh Evans, Jeff Masuda, Nicole Letourneau, Teresa To, Malcolm King

A54 Epigenetic association of *PSORS1C1* and asthma in the Saguenay-Lac-Saint-Jean asthma study

Miriam Larouche, Liming Liang, Catherine Laprise

A55 IL-33 induces cytokine and chemokine production in human mast cells

Stephanie A. Legere, Ian D. Haidl, Jean-Francois Legaré, Jean S. Marshall

A56 Reference ranges for lung clearance index from infancy to adolescence for Canadian population

Zihang Lu, Malcolm Sears, Theo J. Moraes, Felix Ratjen, Per Gustafsson, Wendy Lou, Padmaja Subbarao

A57 Kingston Allergy Birth Cohort: cohort profile and mother/child characteristics to age 2

Michelle L. North, Elizabeth Lee, Vanessa Omana, Jenny Thiele, Jeff Brook, Anne K. Ellis

A58 Cow’s milk protein specific IgE, IgA and IgG4 as a predictor of outcome in oral immunotherapy

Tanvir Rahman, Duncan Lejtenyi, Sarah De Schryver, Ryan Fiter, Ciriaco Piccirillo, Moshe Ben-Shoshan, Bruce Mazer

A59 Age of peanut introduction and development of reactions and sensitization to peanut

Elinor Simons, Allan B. Becker, Rishma Chooniedass, Kyla Hildebrand, Edmond S. Chan, Stuart Turvey, Padmaja Subbarao, Malcolm Sears

A60 Multi-omic blood biomarker signatures of the late phase asthmatic response

Amrit Singh, Casey P. Shannon, Young Woong Kim, Mari DeMarco, Kim-Anh Le Cao, Gail M. Gauvreau, J. Mark FitzGerald, Louis-Philippe Boulet, Paul M. O’Byrne, Scott J. Tebbutt

A61 Early life gut microbial alterations in children diagnosed with asthma by three years of age

Leah T. Stiemsma, Marie-Claire Arrieta, Jasmine Cheng, Pedro A. Dimitriu, Lisa Thorson, Sophie Yurist, Boris Kuzeljevic, Diana L. Lefebvre, Padmaja Subbarao, Piush Mandhane, Allan Becker, Malcolm R. Sears, Kelly M. McNagny, Tobias Kollmann, the CHILD Study Investigators, William W. Mohn, B. Brett Finlay, Stuart E. Turvey

A62 The relationship between food sensitization and atopic dermatitis at age 1 year in a Canadian birth cohort

Maxwell M. Tran, Diana L. Lefebvre, Chinthanie F. Ramasundarahettige, Allan B. Becker, Wei Hao Dai, Padmaja Subbarao, Piush J. Mandhane, Stuart E. Turvey, Malcolm R. Sears

A63 Allergen inhalation enhances Toll-like receptor-induced thymic stromal lymphopoietin receptor expression by hematopoietic progenitor cells in mild asthmatics

Damian Tworek, Delia Heroux, Seamus N. O’Byrne, Paul M. O’Byrne, Judah A. Denburg

A64 The Allergic Rhinitis Clinical Investigator Collaborative – replicated eosinophilia on repeated cumulative allergen challenges in nasal lavage samples

Laura Walsh, Mena Soliman, Jenny Thiele, Lisa M. Steacy, Daniel E. Adams, Anne K. Ellis

A65 The CHILD Study: optimizing subject retention in pediatric longitudinal cohort research

Linda Warner, Mary Ann Mauro, Robby Mamonluk, Stuart E. Turvey

A66 Differential expression of C3a and C5a in allergic asthma

ChenXi Yang, Amrit Singh, Casey P. Shannon, Young Woong Kim, Ed M. Conway, Scott J. Tebbutt

## A1 Role of fibrocytes in allergic rhinitis

### Marie-Ève Côté^1^, Marie-Ève Boulay^1^, Sophie Plante^1^, Jamila Chakir^1^, Louis-Philippe Boulet^1^

#### ^1^Centre de Recherche de l’Institut Universitaire de Pneumologie et de Cardiologie de Québec, Québec, QC, G1V 4G5, Canada

##### **Correspondence:** Marie-Ève Côté - marie-eve.cote@criucpq.ulaval.ca

*Allergy, Asthma and Clinical Immunology* 2016, **12(Suppl 1)**:A1

**Background:** Allergic rhinitis is a major risk factor for asthma development. Lower airway inflammation and remodeling have been observed in allergic rhinitis subjects without asthma. Tissue repair processes involve the production of extracellular matrix, in which fibroblasts play a major role. These cells originate from bone marrow progenitors called fibrocytes. The number of fibrocytes is increased in asthmatics following allergen exposure. In allergic rhinitis, allergen-induced inflammation has been widely observed, but studies on allergen-induced lower airway remodeling are still limited. The aim of this study is to determine the effect of seasonal allergen exposure on the profile of fibrocytes isolated from blood of allergic rhinitis subjects without asthma.

**Methods:** Non asthmatic subjects with seasonal allergic rhinitis were recruited. At baseline (out of the pollen season), medical history, skin prick tests, spirometry, methacholine bronchoprovocation, blood sampling and sputum induction were performed. At the peak of rhinitis symptoms, the tests were repeated. Fibrocytes number and level of activation were determined in whole blood. Cells were stained for fibrocyte markers (CD34, CD45, CXCR4, collagen I) and analyzed by flow cytometry.

**Results:** Thirty subjects (18F:12M) aged 28 ± 8 years were recruited. Among the 12 subjects that completed the study yet, there were 12.2 ± 7.9 % of fibrocytes at baseline, whereas there were 7.1 ± 3.6 % of fibrocytes during the pollen season. Mean fluorescence of CXCR4 was 1614 ± 646 (arbitrary units) at baseline and 1805 ± 770 during the pollen season.

**Conclusion:** The observed decrease in fibrocytes during allergy season may indicate an active migration of these cells from the periphery to the airways. A change in the number and profile of fibrocytes during natural seasonal exposure in non-asthmatic rhinitic subjects may help direct further studies looking at future outcome of these patients to develop a predictor of asthma onset.

## A2 Patterns of aeroallergens sensitization in Northern Alberta

### Hanan Ahmed^1^, Maria-Beatriz Ospina^2^, Kyriaki Sideri^1^, Harissios Vliagoftis^1^

#### ^1^Department of Medicine, University of Alberta, Edmonton, AB, T6G 2B7, Canada, ^2^Alberta Health Services, Respiratory Health Strategic Clinical Network, Edmonton, AB, T6G 2B7, Canada

##### **Correspondence:** Hanan Ahmed - hanan2@ualberta.ca

*Allergy, Asthma and Clinical Immunology* 2016, **12(Suppl 1)**:A2

**Background:** Sensitization to common environmental aeroallergens plays a significant role in the pathogenesis and severity of respiratory allergic disorders [1, 2].Understanding sensitization patterns helps clinicians tailor care more effectively. This is the first study looking at sensitization patterns in Northern Alberta.

**Methods:** Retrospective chart review of skin prick test (SPT) results for 11 environmental aeroallergens performed between January 1st to June 30th 2014 at a University-based lab and clinic, where patients are referred for SPT by allergists, respirologists, general practitioners and ENT specialists. Data was analyzed descriptively and bivariate analyses by sex were done.

**Results:** A total of 623 patients (36.9 % males; 63.1 % females), aged 4–84 years (mean age 38.6 years) had SPT, of which 438 (70.3 %) had a positive test for at least one aeroallergen (atopy). There were no significant sex differences in the frequency of atopy (males: 71.3 % versus females: 69.7 %; p = 0.373). The frequency of sensitivity to particular allergens among atopic subjects was: cat (53.1 %), house dust mite (50.3 %), grass (39.2 %), birch (23.7 %), alternaria (23.7 %), dog (17.3 %), poplar (12.1 %), cedar (9.6 %), aspergillus (9.6 %), hormodendrum (8 %), and penicillium (6.2 %). Of 438 atopic patients, 110 (25.1 %) were mono sensitized, 199 (45.4 %) oligosensitized (2–3 allergens), and 129 (29.5 %) polysensitized (≥4 allergens). There were no significant sex differences in monosensitization (males: 22 % versus females: 27 %; p = 0.143) and oligosensitization (males: 40.9 % versus females: 48.2 %; p = 0.082) rates. Polysensitization was significantly more frequent in males (37.2 %) than in females (24.8 %; p = 0.004).

**Conclusion:** Cat is the most frequent perennial allergen and grass the most frequent seasonal allergen in Northern Alberta. There was no significant difference in the frequency of atopy between males and females. However, males were more likely to be polysensitized than females. Further studies are needed to investigate sensitization patterns among patients with specific allergic conditions.

**References**

1. Barnig C1, Casset A. Respiratory allergens and asthma exacerbation. Rev Mal Respir. 2012;29(6):810–19.

2. Wang J1, Visness CM, Calatroni A, Gergen PJ, Mitchell HE, Sampson HA. Effect of environmental allergen sensitization on asthma morbidity in inner-city asthmatic children. Clin Exp Allergy. 2009;39(9):1381–9.

## A3 Addressing acceptable risk for adolescents with Food-Induced Anaphylaxis (FIA)

### Sara F. Johnson^1^, Roberta L. Woodgate^1^

#### ^1^College of Nursing, University of Manitoba, Winnipeg, MB, R3T 2N2, Canada

##### **Correspondence:** Sara F. Johnson - umjohn94@myumanitoba.ca

*Allergy, Asthma and Clinical Immunology* 2016, **12(Suppl 1)**:A3

**Background:** Adolescents with FIA are more likely to experience fatal anaphylaxis [1] and must learn to independently manage living with FIA and its stigma [2, 3]. These adolescents must make health decisions regarding FIA and personal safety, often using information from Health Care Providers (HCPs). Many studies use risk objectively as a probability, however risk is primarily influenced subjectively by individual context and perception [4]. As a concept used in health education, risk is not well-defined. Using risk objectively when addressing health may cause marginalization and reduced health care participation for adolescents [5, 6]. Therefore discussing risk in terms of what is acceptable for individuals may improve management of FIA. The concept of acceptable risk must be defined for HCPs empowering adolescents with FIA to have the agency to live within their defined levels of wellness.

**Methods:** Walker and Avant’s [7] method for concept analysis was used to analyze and define the concept of acceptable risk in the context of adolescents with FIA.

**Results:** Acceptable risk involves active decision-making for modifiable possibilities where individuals perceive risk and are able to decide whether a situation is safe enough. Using acceptable risk may help to highlight perceived differences in acceptability of risk, and aid in modifying the acceptability of risk levels.

**Conclusions:** The unpredictability and severity of FIA means the ability to manage and cope with risk is important for improving quality of life and safety for adolescents living with FIA. HCPs should address acceptable risk based on individual definitions to engage in and modify perceptions of acceptable risk in daily life. Using acceptable risk to clarify discussions about health management in chronic illness may improve coping, facilitate conversations about safety, reduce shame when unable to comply with ideal management, and empower adolescents living with FIA to take active roles making health decisions.

**Acknowledgements:** SFJ is supported by a University of Manitoba Graduate Fellowship. RLW is supported by a Canadian Institutes of Health Research Applied Chair in Reproductive, Child and Youth Health Services and Policy Research.

**References**

1. Xu YS, Kastner M, Harada L, Xu A, Salter J, Waserman S. Anaphylaxis-related deaths in Ontario: a retrospective review of cases from 1986 to 2011. Allergy Asthma Clin Immunol. 2014;10:38.

2. DunnGalvin A, Gaffney A, Hourihane JO. Developmental pathways in food allergy: a new theoretical framework. Allergy. 2009;64:560–8.

3. Dean J, Fenton NE, Shannon S, Elliott SJ, Clarke A. Disclosing food allergy status in schools: health-related stigma among school children in Ontario. Health Soc Care Community. 2015, in press.

4. McNeill C. Risk: a multidisciplinary concept analysis. Nurs Forum. 2014;49:11–7.

5. Lupton D. Risk and emotion: towards an alternative theoretical perspective. Health Risk Soc. 2013;15:634–47.

6. Spencer G. The “healthy self” and “risky” young other: young people’s interpretations of health and health-related risks. Health Risk Soc. 2013;15:449–62.

7. Walker L, Avant K. Strategies for theory construction in nursing. 5th ed. Norwalk: Appleton & Lange; 2011.

## A4 Outcomes of matched related and unrelated bone marrow transplantation after reduced-toxicity conditioning for children suffering from chronic granulomatous disease

### Guilhem Cros^1^, Pierre Teira^2^, Sonia Cellot^2^, Henrique Bittencourt^2^, Helene Decaluwe^1^, Marie France Vachon^2^, Michel Duval^2^, Elie Haddad^1^

#### ^1^CHU Sainte Justine, Department of Pediatric Hematology–Oncology, University of Montreal, Montreal, QC, Canada, ^2^Department of Pediatric Immunology, University of Montreal, Montreal, QC, Canada

##### **Correspondence:** Guilhem Cros - guilhem.cros@yahoo.fr

*Allergy, Asthma and Clinical Immunology* 2016, **12(Suppl 1)**:A4

Chronic granulomatous disease (CGD) is a life threatening immune deficiency caracterized by severe bacterial or fungal infections, and granulomatous inflammation. The only curative treatment is a hematopoietic stem cell transplantation (HSCT). In 2010, seven children from Sainte Justine hospital were included in an international initiative of HSCT for CGD, based on a reduced toxicity conditioning (RTC), followed by seven others transplanted after the completion of the study.

Data were retrospectively extracted from medical charts for 14 consecutive children transplanted for CGD from 2010 to 2014. Donors were a matched sibling (3/14), or a matched unrelated donor (11/14). The RTC consisted of Fludarabine, targeted Busulfan and rabbit Antithymoglobulin or Alemtuzumab.

The median age at HSCT was 15 years (1–20). Previous history of severe infections included abscesses located in adenopathy (9/14), skin (8/14), liver (7/14) or lungs (10/14), with pulmonary aspergillosis proven for five patients. Septicemia (4/14) and osteomyelitis (2/14) were also common. Inflammation mainly presented as Crohn like colitis (5/14) and chronic lung inflammation (6/14). Three patients had received granulocytes transfusions previously. None patient had active infection or inflammation at the time of transplantation.

All 14 patients engrafted. Severe (grade 3–4) acute graft versus host disease (GVHD) and chronic extensive GVHD were not observed. Three patients experienced immune haemolytic anemia (3/14) and nephrotic syndrome (1/14).

The main complication was secondary graft failure for 3/14 patients occurring at 6 months. All three patients engrafted durably after a second HSCT. Including second grafts, post 6 months after HSCT, chimerism was higher than 90 % in 13/14 patients and 66 % in one patient. With a median follow-up of 43 months (7–60), all patients are alive, cured from CGD and free from immunosuppressive drugs.

HSCT with a RTC and a matched unrelated donor is safe and effective for children with severe forms of CGD.

## A5 Outcomes of patients with severe combined immunodeficiency (SCID) prior to and after initiation of newborn screening for SCID in Ontario

### Vy H.D. Kim^1^, Anne Pham-Huy^2^, Eyal Grunebaum^1^

#### ^1^Division of Immunology/Allergy, Department of Pediatrics, Hospital for Sick Children, Toronto, ON, Canada, ^2^Division of Pediatric Infectious Diseases, Children’s Hospital of Eastern Ontario, Ottawa, ON, Canada

##### **Correspondence:** Vy H.D. Kim - vy.kim@sickkids.ca

*Allergy, Asthma and Clinical Immunology* 2016, **12(Suppl 1)**:A5

**Background:** Severe combined immunodeficiency (SCID) is a life-threatening condition that can be treated by allogeneic hematopoietic stem cell transplantations (HSCT), gene therapy or adenosine deaminase (ADA) replacement. Early diagnosis of SCID might improve patients’ outcome. Newborn screening (NBS) for SCID was initiated in Ontario in August 2013, however the impact on patients is largely unknown.

**Methods:** We analyzed all patients diagnosed with SCID between January 2005 and July 2013 (pre-NBS group) or between August 2013 and June 2015 (post-NBS group) who were referred to the Hospital for Sick Children, Toronto. The SCID aetiology, age of diagnosis, number of infections, length of hospital admissions prior to definitive treatment, age of definitive treatment and survival were compared using Fisher exact test and student’s t tests, when appropriate.

**Results:** Table 1 lists the aetiology of SCID in 21 pre-NBS and 6 post-NBS patients. Pre-NBS, SCID was identified at a mean age of 5.6 months compared to 0.42 months post-NBS (*P* < 0.0001). Post-NBS patients had less infections (1.3/patient versus 3.9/patient, *P* < 0.01) and shorter admissions prior to definitive treatment (mean 27.3 versus 113.8 days, *P* < 0.001). Pre-NBS, three patients received ADA replacement followed by gene therapy in one patient, while 18 patients received HSCT. Post-NBS, ADA replacement was given to 4 patients followed by gene therapy in two patients, while two patients received HSCT. Definitive therapy post-NBS was given at a younger age compared to pre-NBS (mean 61.5 versus 248 days, *P* < 0.0001). All post-NBS patients survived (range 5–23 months) compared to 71 % survival in the pre-NBS group (range 28–121 months).

**Table 1:** Etiology of SCID in patients diagnosed prior to or after initiation of NBS

Type of SCID	Patients identified prior to initiation of NBS	Patients diagnosed after initiation of NBS
ADA deficiency	6	4
X-linked γc chain deficiency	3	1
RAG1 deficiency	2	0
JAK3 deficiency	2	0
CD3δ deficiency	1	0
Cartilage hair hypoplasia	1	0
IL7Rα deficiency	1	0
ZAP70 deficiency	1	0
CORONIN 1A deficiency	1	0
Unknown etiology	3	1
Total	21	6

**Conclusions:** The time to SCID diagnosis and treatment was significantly shorter post-NBS. Less infections and shorter hospital admission prior to treatment occurred post-NBS. Longer follow up and additional patients are needed to better appreciate the impact of NBS on the diagnosis, management and outcome of SCID.

## A6 Detection of regulatory B cells in the airways of subjects with asthma

### John-Paul Oliveria^1^, Stephanie Phan^1^, Mark W Tenn^1^, Damian Tworek^1^, Steven G. Smith^1^, Adrian J. Baatjes^1^, Caitlin D. Obminski^1^, Caroline E. Munoz^1^, Tara X. Scime^1^, Roma Sehmi^1^, Gail M. Gauvreau^1^

#### ^1^Department of Medicine, McMaster University, Hamilton, ON, Canada

##### **Correspondence:** Gail M. Gauvreau - gauvreau@mcmaster.ca

*Allergy, Asthma and Clinical Immunology* 2016, **12(Suppl 1)**:A6

**Background:** Regulatory B cells (B_regs_) have the capacity to regulate and suppress immune responses through production of IL-10 cytokine and IgG_4_ antibodies. The study of B_regs_ in asthma and allergy is a growing field, but few papers have reported on B_regs_ in allergic asthmatics. We previously reported that allergic asthmatics have lower levels of circulating B_regs_ compared to healthy controls. No studies have examined the level of B_regs_ in the airways.

**Methods:** Allergic asthmatics (AA), allergic non-asthmatics (ANA) and healthy controls (HC) donated expectorated sputum samples for B_reg_ analysis. Sputum was induced by inhalation of 3, 4 and 5 % nebulized saline at 7-minute intervals, and cells were isolated from selected mucous plugs and stained with CD5, CD19, CD45, IL-10 and FoxP3. Cells were acquired using a Becton–Dickinson LSRII flow cytometer, analyzed using FlowJo and data were expressed as mean ± SEM. Lymphocytes were characterized as SSC^low^CD45+ and B cells were characterized as SSC^low^CD45+CD19+. GraphPad Prism was used to perform one-way ANOVAs and Tukey post hoc analyses.

**Results:** We quantified the percentages and absolute numbers of B cells (CD19+), and FoxP3-, CD5- and IL-10-expressing B cells within the lymphocyte population of sputum samples (Table 2). We found higher percentages, but not absolute numbers, of B cells in AA compared to ANA and HC (p < 0.05, Table 2). Although we were able to detect B_regs_ in the airways, the percentages and absolute numbers were similar between groups.

**Table 2:** Comparing the percentages and absolute numbers of regulatory B cells in the airways of allergic asthmatics, allergic non-asthmatics and healthy controls

Subset of cells in sputum	Asthmatics (n = 12)	Allergic non-asthmatics (n = 8)	Healthy controls (n = 7)
SSC^low^CD45+/CD45+ (%)	1.5 ± 0.3*	2.8 ± 0.3	2.3 ± 0.5
CD19+/SSC^low^CD45+ (%)	13.4 ± 2.0*	6.6 ± 1.5	4.4 ± 1.0
CD19+/CD45 + (%)	0.19 ± 0.04	0.19 ± 0.05	0.08 ± 0.01
CD19+ (×10^6^ cells/g of sputum)	0.007 ± 0.002	0.011 ± 0.004	0.002 ± 0.0009
CD19+FoxP3+/SSC^low^CD45+ (%)	1.8 ± 0.5	0.6 ± 0.2	0.6 ± 0.3
CD19+FoxP3+/CD45+ (%)	0.02 ± 0.007	0.02 ± 0.004	0.01 ± 0.008
CD19+FoxP3+ (×10^6^ cells/g of sputum) (%)	0.0006 ± 0.0001	0.0007 ± 0.0001	0.0006 ± 0.0004
CD19+FoxP3+CD5+/SSC^low^CD45+ (%)	1.0 ± 0.3	0.4 ± 0.2	0.3 ± 0.1
CD19+FoxP3+CD5+/CD45+ (%)	0.016 ± 0.009	0.010 ± 0.004	0.005 ± 0.002
CD19+FoxP3+CD5+ (×10^6^ cells/g of sputum)	0.0004 ± 0.00018	0.0005 ± 0.00017	0.0002 ± 0.00009
CD19+FoxP3+IL-10+/SSC^low^CD45+ (%)	0.35 ± 0.10	0.16 ± 0.07	0.05 ± 0.02
CD19+FoxP3+IL-10+/CD45+ (%)	0.005 ± 0.0023	0.004 ± 0.0019	0.001 ± 0.0006
CD19+FoxP3+IL-10+ (×10^6^ cells/g of sputum)	0.00013 ± 0.00005	0.00018 ± 0.00007	0.00004 ± 0.00003

* p < 0.05 compared across subject groups

**Conclusions:** The proportion of CD19+ B cells is higher in the airways of AA compared to ANA and HC. B_regs_ are rare cells in the airways; although there were no distinct differences in the levels of B_regs_, it is important to study the function of these cells in order to learn the roles they play in suppressing inflammation in the airways of allergic asthmatics.

## A7 Characterization of IgE-expressing B cells in the airways and peripheral blood of allergic asthmatic subjects

### John-Paul Oliveria^1^, Stephanie Phan^1^, Mark W. Tenn^1^, Brittany M. Salter^1^, Steven G. Smith^1^, Caitlin D. Obminski^1^, Caroline E. Munoz^1^, Abbey Schlatman^1^, Tara X. Scime^1^, Rick Watson^1^, Roma Sehmi^1^, Gail M. Gauvreau^1^

#### ^1^Department Medicine, McMaster University, Hamilton, ON, Canada

##### **Correspondence:** Gail M Gauvreau - gauvreau@mcmaster.ca

*Allergy, Asthma and Clinical Immunology* 2016, **12(Suppl 1)**:A7

**Background:** IgE is critical for allergen-mediated inflammation. IgE is expressed by memory B cells (MBC) and plasmablast (PPC) intermediates, which differentiate into IgE-producing plasma cells (PC). Allergen exposure has been shown to increase IgE levels in the airways of allergic asthmatics; however, B cell subsets expressing and producing IgE in the airways has yet to be studied.

**Methods:** Allergic asthmatics (AA), allergic non-asthmatics (ANA) and healthy controls (HC) were recruited to donate blood and sputum samples. Peripheral blood mononuclear cells (PBMCs) were separated using density gradient centrifugation, and stained with CD19, CD27, CD38, CD45, CD138, IgG and IgE antibodies with appropriate isotype controls. Sputum was induced by inhalation of 3, 4 and 5 % hypertonic saline, and sputum cells were isolated and stained with CD19, CD45, IgE and IgG. Cells were acquired using a Becton–Dickinson LSRII flow cytometer, analyzed using FlowJo and data were expressed as mean ± SEM. Lymphocytes were defined as SSC^low^CD45+, and B cells were defined as SSC^low^CD45+CD19+. GraphPad Prism was used to perform one-way ANOVAs and Tukey post hoc analyses.

**Results:** Circulating levels of B cells and IgE-expressing B cell subsets, including MBCs (CD27+CD38−), PPCs (CD27+CD38+) and PCs (CD38+CD138+) were similar in percentages and absolute numbers between AA, ANA and NC. In contrast, the level of IgE-expressing B cells in sputum was higher in AA compared to ANA and HC, and this was evident when cells were expressed as percentages or as absolute numbers (p < 0.05, Table 3).

**Table 3:** Comparing the percentages and absolute numbers of IgE-expressing B cells in the airways and blood of allergic asthmatics, allergic non-asthmatics and healthy controls

Subset of cells	Asthmatics blood (n = 11)	Allergic non-asthmatics blood (n = 11)	Healthy controls blood (n = 7)
SSC^low^CD45+/CD45+ cells (%)	46.2 ± 3.9	38.3 ± 4.6	50.5 ± 5.1
CD19+/SSC^low^CD45+ (%)	11.4 ± 1.0	13.2 ± 0.9	8.6 ± 1.3
CD19+/CD45+ (%)	6.0 ± 0.5	4.4 ± 0.6	4.4 ± 0.8
CD19+ (×10^6^ cells/mL of blood)	0.38 ± 0.07	0.26 ± 0.04	0.31 ± 0.06
CD19+IgE+/SSC^low^CD45+ (%)	0.24 ± 0.05	0.13 ± 0.03	0.27 ± 0.15
CD19+IgE+/CD45+ (%)	0.15 ± 0.04	0.06 ± 0.02	0.12 ± 0.06
CD19+IgE+ (×10^6^ cells/mL of blood)	0.010 ± 0.004	0.004 ± 0.001	0.007 ± 0.003

Subset of cells	Asthmatics sputum (n = 10)	Allergic non-asthmatics sputum (n = 7)	Healthy controls sputum (n = 6)
SSC^low^CD45 +/CD45+ (%)	1.6 ± 0.4	2.0 ± 0.8	1.9 ± 0.4
CD19+/SSC^low^CD4+ (%)	11.8 ± 2.0*	5.7 ± 1.0	4.6 ± 0.6
CD19+/CD45+ (%)	0.18 ± 0.04	0.11 ± 0.03	0.08 ± 0.02
CD19+ (×10^6^ cells/g of sputum)	0.008 ± 0.002	0.005 ± 0.003	0.002 ± 0.0003
CD19+IgE+/SSC^low^CD45+ (%)	3.5 ± 1.0*	0.4 ± 0.1	0.2 ± 0.1
CD19+IgE+/CD45+ (%)	0.048 ± 0.011*	0.007 ± 0.002	0.004 ± 0.002
CD19+IgE+ (×10^6^ cells/g of sputum	0.0020 ± 0.00063*	0.0003 ± 0.00009	0.0001 ± 0.00004

* p < 0.05 compared across subject groups

**Conclusions:** Compared to allergic non-asthmatic subjects and healthy controls, subjects with allergic asthma demonstrated higher levels of IgE-expressing B cells in their airways, but not blood. This suggests localization of IgE-expressing B cells in the airways of allergic asthmatics, where local production of IgE in the airways may be important in the pathogenesis of allergic asthma.

## A8 Pregnancy: could it be a risk factor for primary immunodeficient patients

### Roya Sherkat^1^, Razieh Khoshnevisan^1^, Saba Sheikhbahaei^1^

#### ^1^Acquired Immunodeficiency Research Center, Isfahan University of Medical Sciences, Isfahan, Iran

##### **Correspondence**: Roya Sherkat - sherkat@med.mui.ac.ir

*Allergy, Asthma and Clinical Immunology* 2016, **12(Suppl 1)**:A8

**Background:** Primary immunodeficiency disorder (PID) refers to a heterogeneous group of disorders characterized by poor or absent function in one or more components of the immune system. More than 250 different disorders have been identified to date, with new disorders continually being recognized [1].Treatment in PID patients has reduced the mortality and morbidity. Thus, such patients often survive into adulthood. [2, 3]Although it is likely that more women with PID will wish to become pregnant in the future, only a few such cases have been reported to date. In this article PID pregnant cases and their management has been evaluated.

**Methods:** The PID cases that became pregnant have evaluated and followed in PID clinic, Isfahan University of Medical sciences between 1997 and 2014. Depend on variety of immunodeficiency, supportive and definitive treatment has been done for pregnant patients.

**Result:** Two patients had pregnancy and were followed and managed. A CGD patient with history of recurrent infections such as empyema, pneumonia, TB lymphadenitis, cutaneous abscess, mouth ulcer and gingivitis whose pregnancy course was uneventful. After normal vaginal delivery, she faced to severe low back pain, sacral fungal osteomyelitis and granulomatous lesion all over her uterus and liver who has been treated with antifungal and interferon gamma.

Another PID case with CVID and history of upper and lower respiratory tract infections and thrombocytopenia who has been managed during her pregnancy period and delivery for severe thrombocytopenia by higher dose of IVIG and even platelet infusion during her cesarean section.

**Conclusion:** The treatment of PIDs is complex and generally requires both supportive and definitive strategies. The pregnancy potentially is high risk and immune regulation switch may provide more complication and disorders during pregnancy. According to the type of PID, management and treatment of PID patients could be different and future researchers in this area have to be done.

**References**

1. Al-Herz W, Bousfiha A, Casanova JL, et al. Primary immunodeficiency diseases: an update on the classification from the international union of immunological societies expert committee for primary immunodeficiency. Front Immunol. 2014;5:162.

2. Hisano M, Kobayashi S, Arata N, Murashima A, Yamaguchi K. Successful completion of pregnancy in a woman with chronic granulomatous disease. Obstet Med. 2011;4:174–6.

3. Shaley E, Ben-Ami M, Peleg D. Common variable hypoglobulinemia and pregnancy. Br J Obstet Gynecol. 1993;100:1138–40.

## A9 Clinical experience with Octagam: a Canadian retrospective chart review

### Stephen Betschel^1^, Richard Warrington^2^, Robert Schellenberg^3^

#### ^1^Division of Allergy and Clinical Immunology, University of Toronto, Toronto, ON, Canada, ^2^Division of Allergy and Clinical Immunology, University of Manitoba, Winnipeg, MB, Canada, ^3^Division of Allergy and Immunology, University of British Columbia, Vancouver, BC, Canada

##### **Correspondence:** Stephen Betschel - BetschelS@smh.ca

*Allergy, Asthma and Clinical Immunology* 2016, **12(Suppl 1)**:A9

In Canada intravenous immune globulin (IVIg) products are licensed for eight disease indications. Although individual IVIg products are not approved for the same indications, Canadian hospitals stock a mix of IVIgs leading to their generic use. IVIG products are used for antibody replacement therapy and in many autoimmune diseases. First approved in Germany in 1995, Octagam^®^ products have been in clinical use for over 20 years in 80 countries. In Canada, Octagam^®^ 10 % has been available since 2013. Here we report the routine clinical use of Octagam^®^ 10 % across three Canadian institutions, and compare its use to other IVIg products licensed in Canada.

**Fig. 1:**
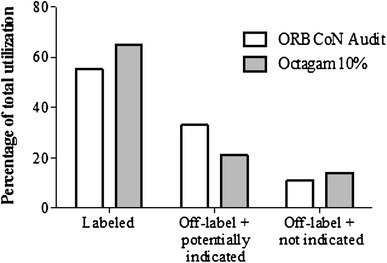
Comparison of the ORBCoN audit findings for IVIg utilization in Ontario to Octagam^®^ 10 % utilization across three Canadian Institutions highlighting labeled, off-label and potentially indicated, versus off-label and not indicated usage

A total of 135 patients were treated with Octagam^®^ 10 % for 28 different conditions represented by five distinct indication groups: immunology (43 %), hematology (28 %), neurology (17 %), rheumatology (5 %), and infectious disease (3 %). Ontario Regional Blood Coordinating Network (ORBCoN) recently published an audit of the routine clinical use of IVIg products across Ontario [1]. The audit included 2246 patients over a 3-month period representing 120 indications. The audit captured labeled versus unlabeled utilization. Labeled indications referred to those that were approved for use by Health Canada for at least one IVIg product. The off-label utilization was categorized as off-label and potentially indicated, or off-label and not indicated. Applying the same criteria set forth in the ORBCoN audit, each of the indications identified in this study were similarly classified (Fig. 1).

The results of this retrospective chart review indicate that Octagam^®^ 10 % is prescribed to Canadian patients similar to other IVIg products, with 85 % of the utilization deemed appropriate based on current IVIg guidelines [2–4]. Although adverse reactions and efficacy data was not reported, the acceptance of its use suggests Octagam^®^ 10 % has a comparable safety and efficacy profile to other IVIg products.

**References**

1. ORBCoN. Intravenous Immune Globulin (IVIG) 2012 Audit Report. Ontario Regional Blood Coordinating Network 2012. http://transfusionontario.org/en/cmdownloads/2012-ivig-audit-report/. Accessed 31 May 2015.

2. Nahirniak S, Hume HA. Guidelines for the use of immunoglobulin therapy for primary immune deficiency and solid organ transplantation. Transfus Med Rev. 2010;24(Suppl 1):S1–6.

3. Anderson D, Ali K, Blanchette V, Brouwers M, Couban S, Radmoor P et al. Guidelines on the use of intravenous immune globulin for hematologic conditions. Transfus Med Rev. 2007;21(2 Suppl 1):S9–56.

4. Feasby T, Banwell B, Benstead T, Bril V, Brouwers M, Freedman M et al. Guidelines on the use of intravenous immune globulin for neurologic conditions. Transfus Med Rev. 2007;21(2 Suppl 1):S57-107.

## A10 Kounis syndrome secondary to contrast media with inferior ST elevations and bilateral ischemic stroke

### Michael N. Fein^1^, Jean-Philippe Pelletier^2^

#### ^1^Department of Clinical Immunology and Allergy, McGill University, Montreal, QC, H3G 1A4, Canada, ^2^Department of Cardiology, McGill University, Montreal, QC, H3G 1A4, Canada

##### **Correspondence:** Michael N. Fein - Michael.fein@mail.mcgill.ca

*Allergy, Asthma and Clinical Immunology* 2016, **12(Suppl 1)**:A10

**Background:** Kounis syndrome is a rare but increasingly recognized clinical entity whereby anaphylactic or anaphylactoid reactions provoke coronary artery spasm that may lead to acute myocardial infarction. The pathogenesis is thought to be secondary to mast cell activation-degranulation [1]. A “Kounis-like” syndrome has been described in two patients with mast cell activation disorders presenting with neurologic symptoms yet no evidence of stroke on extensive imaging [2].

**Case presentation:** A 62-year-old male with a history of dyslipidemia and hypothyroidism presented with a three-day history of nausea, vomiting, fevers, and abdominal pain. There were no complaints of chest pain or shortness of breath. On exam he was vitally well but had significant left lower quadrant tenderness. His initial blood work revealed a leukocytosis of 12.3 × 10^9/L, a C-reactive protein of 78 mg/L, and a lactate of 0.9 mmol/L.

During an abdominal computerized tomography (CT) scan with contrast, a code blue was called when the patient became hypotensive, cyanotic, and lost consciousness. He was intubated and an electrocardiogram (ECG) revealed ST elevations in leads II, III, and aVF. A new urticarial rash was now present on his abdomen. He was treated with intravenous fluids, steroids, and anti-histamines with rapid recovery of his blood pressure. Twenty minutes later a repeat ECG showed resolution of the ST elevations; however the troponin level did rise to 4.43 μg/L. Off sedation, the patient had decreased level of consciousness. A head CT revealed multiple hypo-densities in both cerebral hemispheres consistent with cortical and subcortical infarctions in the middle cerebral artery territories, thought to be secondary to hypo-perfusion and cerebral vasospasm.

**Conclusion:** Kounis syndrome has been associated with a wide range of medical conditions, drugs, and environmental exposures. We describe the first case of Kounis syndrome secondary to contrast media with evidence of coronary vasospasm and bilateral ischemic stroke.

**Consent:** Written informed consent was obtained from the patient for publication of this case report and any accompanying images.

**References**

1. Kounis NG. Kounis syndrome (allergic angina and allergic myocardial infarction): a natural paradigm? Int J Cardiol. 2006;110:7–14.

2. González-de-Olano D, Alvarez-Twose I, Matito A, Sánchez-Muñoz L, Kounis NG, Escribano L. Mast cell activation disorders presenting with cerebral vasospasm-related symptoms: a “Kounis-like” syndrome? Int J Cardiol. 2011;150(2): 210–1.

## A11 Honey bee venom immunotherapy ineffective in bumble bee-induced anaphylaxis: case report and review of literature

### Manstein Kan, Robert Schellenberg^1^

#### ^1^Division of Allergy and Immunology, Department of Medicine St Paul’s Hospital, University of British Columbia, Vancouver, BC, V6Z 1Y6, Canada

##### **Correspondence:** Manstein Kan - mansteinkan@alumni.ubc.ca

*Allergy, Asthma and Clinical Immunology* 2016, **12(Suppl 1)**:A11

**Background:** Bumble bee allergy is an uncommon cause of Hymenoptera venom allergy. Studies have suggested a variable degree of immunological cross reactivity between bumble bee and honeybee venoms [1]. Bumble bee venom is not available for skin testing in Canada.Treating bumble bee allergic patients with honeybee venom immunotherapy has been described in the literature [2]. We present a case of unsuccessful honey bee immunotherapy in a patient with a previous anaphylactic reaction to eastern North American bumble bee, *Bombus impatiens*.

**Case presentation:** A 32 year old laboratory worker with bumble bees had a number of stings over years without reaction. In 2009 he had three stings within a few months and developed hypotension, generalized urticaria and wheezing immediately after the third sting. Serum IgE levels were markedly elevated for both honey bee and bumble bee and he was commenced on honey bee immunotherapy at another centre, being maintained on 100 mcg monthly injections for 5 years with total compliance. He was not stung over this period. On subsequent evaluation he was negative to intradermal testing (0.02 mL) with honey bee venom at 1 mcg/mL. A sting challenge to western bumble bee (*Bombus occidentalis)* was done and the patient developed immediate anaphylaxis symptoms and required treatment with epinephrine and antihistamine.

**Conclusions:** Without access to IgE testing to specific antigens contained in various Apidae species, cross-reactivity canntot be determined. Immunotheapy with honey bee venom did not protect our patient with bumble bee allergy. Bumble bee venom immunotherapy has been reported in Europe but European species vary significantly from North American species. Our experience suggest significant similarity in allergen content between eastern and western bumble bee venoms. With incresing use of bumble bees in agriculture, allergic reactions to stings may become more prevalent and access to venoms for testing and immunotherapy more important.

**Consent:** Written informed consent was obtained from the patient for publication of this case report and any accompanying images.

**References**

1. Stapel SO, Waanders-Lijster de Raadt J, van Toorenenbergen AW. Allergy to bumblebee venom. II. IgE cross-reactivity between bumblebee and honeybee venom. J Allergy.1998;53:769–77.

2. Kochuyt AM, Van Hoeyveld E, Stevens EA. Occupational allergy to bumble bee venom. Clin Exp Allergy. 1993;23:190–5.

## A12 Delayed immune reconstitution occurring after multiple immune complications of hematological stem cell transplantation for a leaky SCID

### Roxane Labrosse^1^, Guilhem Cros^1^, Pierre Teira^2^, Henrique Bittencourt^2^, Helene Decaluwe^1^, Michel Duval^2^, Elie Haddad^1^

#### ^1^CHU Sainte Justine, Department of Pediatric Immunology, University of Montreal, Montreal, QC, Canada, ^2^Department of Pediatric Hematology–Oncology, University of Montreal, Montreal, QC, Canada

##### **Correspondence:** Roxane Labrosse - roxane.labrosse@umontreal.ca

*Allergy, Asthma and Clinical Immunology* 2016, **12(Suppl 1)**:A12

**Case report:** We report a 7-year-old patient with persistant and refractory auto-immune complications post-hematological stem cell transplantation (HSCT). He was diagnosed at 3 years of age with a leaky SCID (homozygous mutations of RAG1) after several auto-immune complications and repeated infections. He underwent a mismatched umbilical cord blood transplantation (5/6) at 40 months with a conditioning regimen of Busulfan, Cyclophosphamide and rabbit ATG. His hematological reconstitution was good and he was platelet transfusion-free by day 59 post-HSCT. Chimerism stayed 100 % donor from day 100 post-HSCT.

He presented severe acute and chronic GvHD adequately controlled with heavy immunosuppression until 2 years post HSCT. Two and half years post-HSCT, he presented with profound peripheral pancytopenia. As this pancytopenia was resistant to Prednisone, immunoglobulins and rituximab, he received two injections of Alemtuzumab. While red and white blood cells recovered completely, he presented a persistant and refractory immune thrombopenia (ITP) despite treatment with prednisone, sirolimus, Rituximab, and two courses of bortezomib. A splenectomy was performed with transient efficacy, and romiplostim was added to his treatment shortly after.

Concurrently, our patient had very poor immune reconstitution. His CD4+T lymphocytes stayed inferior to 200/µl and the emigrant thymic naive T cells (CD45RA+CD31+/CD4 cells) were nonexistent. As the thymic function seemed to be absent, thymic transplantation was discussed. However, with the combination of splenectomy and romiplostim as platelet-enhancing therapies, we were able to progressively wean prednisone therapy down to a minimal dose of 0.3 mg/kg/day with subsequent restoration of thymic function, as shown by uptrending of CD4+ T lymphocytes and emigrant thymic naive T cells, with latest values of 561/µl and 38 % respectively.

**Consent:** Written informed consent was obtained from the patient for publication of this case report and any accompanying images.

## A13 Comparison of three case reports of acquired angioedema: presentation, management and outcome

### Raymond Mak^1^, James Loh^2^, Amin Kanani^2^

#### ^1^Internal Medicine, University of British Columbia, Vancouver, BC V5Z 1M9, Canada, ^2^Division of Clinical Immunology and Allergy, University of British Columbia, Vancouver, BC V5Z 1M9, Canada

##### **Correspondence:** Raymond Mak - raymondtmak@yahoo.com

*Allergy, Asthma and Clinical Immunology* 2016, **12(Suppl 1)**:A13

**Background:** Acquired angioedema is an acquired form of C1 deficiency, where inappropriate bradykinin release can result in skin swelling (face and limbs), severe abdominal pain from gastrointestinal mucosal edema and life-threatening upper airway swelling. It has been reported in patients with lymphoproliferative disorders. Autoantibodies to C1 inhibitor are present in many of these individuals.

**Clinical presentation:** Three cases of acquired angioedema are presented. All three individuals presented over several years with recurrent episodes of angioedema and eventually, required ICU admission and intubation for airway angioedema. Their lab work demonstrated low C4 and C1 inhibitor levels. Given later onset of symptoms and lack of family history, they were diagnosed with acquired angioedema. The C1q assay was not readily available to confirm the diagnoses.

Case 1, a 76 year old female, had an abnormal peripheral smear, SPEP and bone marrow aspirate suggestive her angioedema was secondary to a lymphoproliferative process. Case 2, a 77 year old male later developed colon cancer but his angioedema did not improve with cancer treatment, so the two were felt to be unrelated. Case 3, currently a 56 year old male presented in his early 50s. He underwent gene testing for hereditary angioedema which was negative. His laboratory tests revealed a small monoclonal band on serum protein electrophoresis.

**Management and outcomes:** The first two patients were treated with danazol prophylaxis. One case had fluctuating levels of C1inh and C4 that did not correlate with danazol dosage or symptoms. Danazol for both cases was titrated to symptoms and maintained at an average dose of 200 mg per day. The third case had limited clinical response to various doses of danazol and initially required scheduled C1 inhibitor replacement. Eventually, he was treated with tranexamic acid for prophylaxis. He has been well controlled with tranexamic acid 1500 mg TID.

**Consent:** Written informed consent was obtained from the patient for publication of this case report and any accompanying images.

## A14 Sitagliptin-associated angioedema not related to concurrent use of ARB or ACE inhibitor

### Dominik A. Nowak^1^, Paul K. Keith^1,2^

#### ^1^School of Medicine, McMaster University, Hamilton, ON, L8S 4L8, Canada, ^2^Division of Clinical Immunology and Allergy, Department of Medicine, McMaster University, Hamilton, ON, L8S 4L8, Canada

##### **Correspondence:** Dominik A. Nowak - dominik.nowak@medportal.ca

*Allergy, Asthma and Clinical Immunology* 2016, **12(Suppl 1)**:A14

**Background:** There are limited reports linking the dipeptidyl peptidase-IV (DPP-IV) inhibitor sitagliptin with upper airway angioedema. Most reports thus far suggest an interaction with the concurrent use of an angiotensin II receptor blocker (ARB) or an angiotensin-converting-enzyme (ACE) inhibitor. The proposed mechanism is shared inhibition of the bradykinin degradation pathway by ACE or DPP-IV inhibitor, or increased effect of bradykinin while on ARB.

**Case:** In March 2015, a 100 pack year former smoker was referred to our Adverse Reactions Clinic for recurrent episodes of lip, tongue, and throat swelling. These episodes occurred 3–4 times a month for several years, with numerous emergency department visits. Our patient had been on prednisone 5 mg daily for several years to manage these episodes, increasing to 40 mg during an exacerbation. They previously had angioedema while taking an ACE inhibitor and possibly following aspirin. They also had seasonal allergies, a rash with amoxicillin, and GI upset with glyburide. They managed their diabetes with insulin. Their other medications included sitagliptin/metformin (Janumet^®^), atorvastatin, hydrochlorothiazide, furosemide, diltiazem, cetirizine, domperidone, ezetimibe, levothyroxine, pantoprazole, ranitidine, vitamin D, ferrous fumarate, B12 injections, docusate, and the following as needed: epinephrine autoinjector, nitroglycerin, senekot.

**Investigations:** Previous investigations for hereditary angioedema and systemic mastocytosis negative. CRP 10.2 mg/mL (reference range <5.0 mg/L), IgE 15 (<165 KIU/L), and Vitamin D 62 nmol/L (>75 optimal). Routine bloodwork, immunoglobulins, autoimmune screen, C3, C4, C1 esterase inhibitor (q/f), and tryptase otherwise normal each time repeated between 2011–2015.

**Course:** We advised our patient to discontinue sitagliptin and take metformin alone. They have had no further emergency department visits for angioedema.

**Discussion:** Sitagliptin, like ACE inhibitors but not to the same extent, may prevent the breakdown of bradykinin. ACE inhibitors are also much more likely to cause swelling than ARBs. Although ARBs do not inhibit the breakdown of bradykinin, they block bradykinin two receptors and make more available to cause swelling. It is thus possible that sitagliptin played a role in this patient’s recurrent episodes of angioedema, especially given the onset of the symptoms after the initiation of the drug ten years ago, the disappearance of symptoms with its discontinuation, and the similar previous problems with ACE inhibitors. Our patient had several risk factors that would increase her risk of angioedema while on ACE inhibitors (and potentially DPP-IV inhibitors). Generally, risk factors associated with ACE inhibitor-induced angioedema include previous or current smoking, female gender, seasonal allergies, and “African American race”, while having diabetes is protective (as reported in the Omapatrilat Cardiovascular Treatment versus Enalapril [OCTAVE] study). Her presentation was typical of bradykinin-dependent angioedema, namely, swelling lasting several days, without pruritus or urticaria, and being somewhat refractory to corticosteroids.

## A15 Sneddon–Wilkinson subcorneal pustular dermatosis associated with an IgA monoclonal gammopathy

### Daniel Pannozzo^1^, Dominik A. Nowak^1^, Hermenio C. Lima^1,2^

#### ^1^School of Medicine, McMaster University, Hamilton, ON, L8S 4L8, Canada, ^2^Division of Dermatology, Department of Medicine, McMaster University, Hamilton, ON, L8S 4L8, Canada

##### **Correspondence:** Daniel Pannozzo - daniel.pannozzo@medportal.ca

*Allergy, Asthma and Clinical Immunology* 2016, **12(Suppl 1)**:A15

**Background:** Subcorneal pustular dermatosis (SCPD) is a rare benign chronic inflammatory skin disorder of unknown etiology. First described by Sneddon and Wilkinson in 1956, it is characterized by a relapsing course of symmetric subcorneal sterile pustules involving the flexures, proximal limbs, and trunk. SCPD is often associated with a benign monoclonal IgA gammopathy, which can either precede or follow diagnosis. [1].

**Fig. 2:**
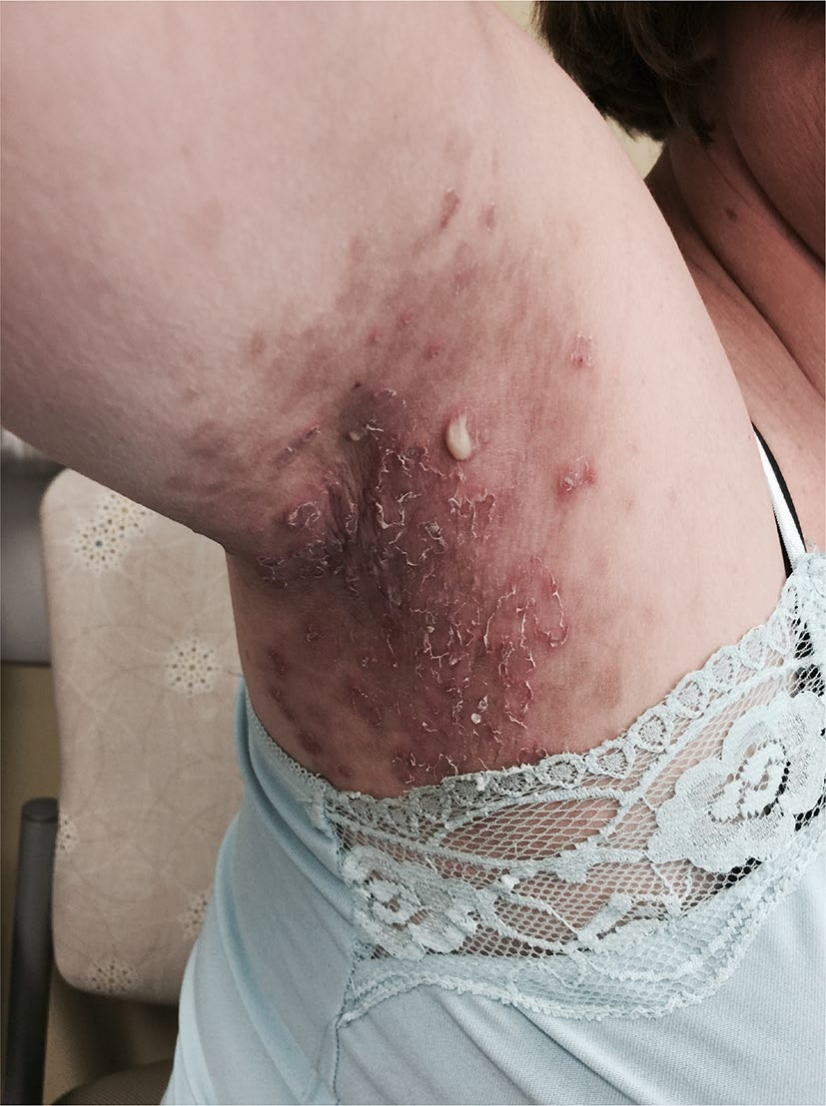
Flaccid pustules measuring several millimetres in diameter on mildly erythematous skin. The *image* shows a classic half-and-half blister in which purulent fluid accumulates in the lower half of the blister

**Case:** A 56-year old Caucasian female presented to our outpatient immunology-dermatology clinic with a seven-year relapsing history of a mildly pruritic and irritating pustular skin eruption under the arms, breasts, and around the groin. Physical examination showed several pea-sized flaccid pustules on an erythematous base in the axillae, groin, and submammary regions (Fig. 2). The patient was otherwise well with no signs of systemic disease.

**Investigations:** Routine blood work including a complete blood count and liver function tests were normal. Serum protein electrophoresis showed an abnormal IgA monoclonal gammopathy lambda type with a value of 4.63 g/l (normal 0.7–3.52 g/l). Histopathology showed a sterile subcorneal pustule with inflammatory infiltrate of lymphocytes and neutrophils with the absence of acantholysis. Her previous treatments included topical corticosteroids and antibiotics as well as oral cephalexin and itraconazole, all with poor response.

**Discussion:** The main differential diagnosis of SCPD includes pustular psoriasis, pemphigus foliaceus, IgA pemphigus, impetigo, dermatitis herpetiformis, and acute generalized exanthematous pustulosis. Our patient had been previously treated with antibiotic and antifungal agents with poor response for an infectious cause of the disease. Clinicians may consider an inflammatory disease in their differential when presented with a pustular eruption in the interigenous areas.

**Consent:** Written informed consent was obtained from the patient for publication of this case report and any accompanying images.

**Reference**

1. Cheng S, Edmonds E, Ben-Gashir M, Yu RC. Subcorneal pustular dermatosis: 50 years on. Clin Exp Dermatol. 2008;33(3):229–33. doi:10.1111/j.1365-2230.2008.02706.x.

## A16 Omalizumab can be effective in patients with allergic bronchopulmonary aspergillosis

### Diana Pham^1^, Hoang Pham^3^, Gonzalo G. Alvarez^2,3^, Istvan T. Bencze^2,3^, Krishna B. Sharma^2,3^, Mark Smith^3^, Shawn Aaron^2,3^, Jennifer Block^3^, Tara Keays^3,4^, Judith Leech^2,3^, David Schneidermen^3,4^, Jodi Cameron^1^, Jennifer Forgie^1^, Alicia Ring^1^, John W. O’Quinn^1^, Stephanie Santucci^1^, William H. Yang^1,3^

#### ^1^Ottawa Allergy Research Corporation, Ottawa, ON, Canada, ^2^Division of Respiratory Medicine, The Ottawa Hospital, Ottawa, ON, Canada, ^3^University of Ottawa Medical School, Faculty of Medicine, Ottawa, ON, Canada, ^4^Division of Internal Medicine, Montfort Hospital, Ottawa, ON, Canada

##### **Correspondence:** Diana Pham - dpham@yangmedicine.com

*Allergy, Asthma and Clinical Immunology* 2016, **12(Suppl 1)**:A16

**Background:** Allergic bronchopulmonary aspergillosis (ABPA) is a challenging respiratory disease with significant morbidity and mortality. Among patients with severe asthma, 10 % have ABPA. Typically they have a decline in lung function, positive skin prick reaction to *Aspergillus*, elevated IgE levels, increased *Aspergillus fumigatus* specific IgE antibody and central bronchiectasis. Patients often exhibit refractory symptoms despite conventional asthma therapy. Long-term use of high dose inhaled and oral corticosteroids may lead to serious adverse effects such as immunosuppression, adrenal insufficiency, metabolic syndrome, hypokalemia, glaucoma, cataracts, peptic ulcers, osteoporosis, avascular necrosis, and psychiatric disturbances. Antifungals have many drug interactions and may require therapeutic monitoring. Some studies seem to suggest that omalizumab may be a better therapeutic option as there is some evidence demonstrating efficacy in ABPA and fewer adverse effects than long-term corticosteroids.

**Methods:** A retrospective chart review was performed for ABPA patients receiving omalizumab between 2007 and 2015 at our tertiary care Allergy and Asthma Clinic and The Ottawa Hospital Chest Clinic. Data was collected on demographics, total IgE, aspergillus specific skin testing, the use of inhaled and oral corticosteroids, quality of life (QoL) questionnaires, and number of asthma exacerbations/hospitalizations.

**Results:** A total of ten patients were evaluated (59.7 ± 13.9 years, 7 females and 3 males). Compared to their baseline, the average dose of inhaled corticosteroids dropped by 24 % at month 16 and out of the three patients who were dependent on oral corticosteroids, there was an 89 % decrease in prednisone use by month 16. QoL scores improved in relation to baseline. Nine of the subjects that reported exacerbations 12 months prior to treatment and none reported exacerbations once they started omalizumab.

**Conclusion:** Our retrospective study provides evidence to support the fact that omalizumab may be an effective low-risk treatment for patients with ABPA. Further prospective studies are required to confirm this finding.

**Consent:** Written informed consent was obtained from the patient for publication of this case report and any accompanying images.

## A17 Efficacious use of omalizumab in the treatment of cystic fibrosis

### Diana Pham^1^, Hoang Pham^3^, Ena Gaudet^2^, Shawn Aaron^2,3^, Stephanie Santucci^1^, William H. Yang^1,3^

#### ^1^Ottawa Allergy Research Corporation, Ottawa, ON, Canada, ^2^Division of Respirology, The Ottawa Hospital, Ottawa, ON, Canada, ^3^University of Ottawa, Faculty of Medicine, Ottawa, ON, Canada

##### **Correspondence:** Diana Pham - dpham@yangmedicine.com

*Allergy, Asthma and Clinical Immunology* 2016, **12(Suppl 1)**:A17

**Background:** Cystic fibrosis (CF) is an autosomal recessive disease associated with airway obstruction and chronic lung infections. Allergic bronchopulmonary aspergillosis (ABPA) complicates 7–9 % of CF cases. The first-line therapies for treating ABPA are corticosteroids and antifungal drugs. Long-term use of corticosteroids may result in very serious adverse effects such as immunosuppression, adrenal insufficiency, and diabetes. Antifungals, mucolytics, pancreatic enzymes and bronchodilators are also other treatment modalities. A few studies suggest omalizumab may be effective in CF. Here we report a 25-year old male CF patient with concomitant ABPA complicated with numerous hospitalizations and steroid side effects who responded well to omalizumab.

**Methods:** A retrospective chart review was performed. Evaluation jointly occurred at The Ottawa Hospital’s CF Clinic and a tertiary care Allergy and Asthma Clinic in Ottawa.

**Results:** The patient was diagnosed with CF at 5 months old. This patient had a positive skin prick reaction to *Aspergillus fumigatus* and a total serum IgE of 111.7 IU/mL. He developed corticosteroid-induced diabetes at age 19. Given his poor health and high IgE levels, a decision was made to attempt a trial of omalizumab. After 8 months of omalizumab, he successfully weaned off of prednisone. After 18 months, the patient no longer required insulin to treat his prednisone-induced diabetes. After 20 months, his quality of life improved. During the 12 months prior to treatment he was hospitalized 8 times. He had only one hospitalization since month seven. He was considered for a double lung transplant, but after 1 year of treatment, transplantation was no longer for consideration because he stopped needing daily home oxygen.

**Conclusion:** Omalizumab had a dramatic steroid sparing effect, reduced hospitalizations and O_2_ requirements in this CF patient with ABPA and prednisone-induced diabetes. Further prospective studies using a larger cohort are necessary to make any clinical recommendations.

**Consent:** Written informed consent was obtained from the patient for publication of this case report and any accompanying images.

## A18 HAE with normal C1-INH with inconsistent response to C1 esterase inhibitor infusion but reliably responsive to icatibant

### Hoang Pham^1^, Stephanie Santucci^2^, William H. Yang^1,2^

#### ^1^University of Ottawa, Faculty of Medicine, Ottawa, ON, Canada, ^2^Ottawa Allergy Research Corporation, Ottawa, ON, Canada

##### **Correspondence:** Hoang Pham - tpham077@uottawa.ca

*Allergy, Asthma and Clinical Immunology* 2016, **12(Suppl 1)**:A18

**Background:** Hereditary angioedema (HAE) with normal C1-esterase inhibitor (C1-INH) is subdivided into factor XII mutation or of unknown origin (U-HAE). The diagnosis is based on clinical symptoms of recurrent edema (commonly skin swellings, tongue swelling), family history, and normal C1-INH quantity and function. U-HAE is presumed when factor XII mutation testing is negative. Here we present a 65 year old male with suspected U-HAE, who has a 30-year personal history of recurrent upper airway swelling, family history, and normal C1-INH quantity and function. He initially responds to empiric C1-INH. However, he seems to have developed resistance to C1-INH, requiring rescue therapy with icatibant.

**Methods:** For each treatment, a log method was used to collect information on: attack intensity, anatomical location, number of doses, onset of relief, time elapsed until complete resolution. Hospital records and patient-reports were collected for each treatment received through the emergency department.

**Results:** This patient was first seen September 2013 for suspected HAE. C4 and C1 INH quantities and function were repeatedly normal. In October 2013, the patient began IV treatment with CI-INH for the first time with good response. In June and October 2014, treatment with multiple doses of C1-INH was required to achieve resolution of symptoms. In February 2015, despite multiple C1-INH doses, the patient was intubated and admitted to ICU for tongue swelling with throat involvement, which resolved slowly over 4 days. In April 2015, icatibant was used for treatment in the ED when on tongue swelling occurred and was unresponsive to C1-INH. Swelling began to subside within 1 h of the icatibant administration. Since then, the patient has had many documented swellings that have not responded to C1 but have responded to icatibant.

**Conclusion:** Icatibant can be an effective treatment for suspected U-HAE when treatment response with intravenous C1 esterase inhibitor is inadequate.

**Consent:** Written informed consent was obtained from the patient for publication of this case report and any accompanying images.

## A19 Anaphylaxis reaction to lactase enzyme

### Mathew R. Voisin^1^, Rozita Borici-Mazi^2^

#### ^1^School of Medicine, Faculty of Health Sciences, Queen’s University, Kingston, ON, K7L 3N6, Canada, ^2^Division of Allergy and Immunology, Department of Medicine, Queen’s University, 166 Brock Street, Kingston, ON, K7L 5G2, Canada

##### **Correspondence:** Mathew R. Voisin - mvoisin@qmed.ca

*Allergy, Asthma and Clinical Immunology* 2016, **12(Suppl 1)**:A19

**Background:** Lactose intolerance affects approximately 20 % of Canadians and roughly 70 % of the world’s population, leading to symptoms of flatulence and bloating after the ingestion of lactose-containing foods. Lactose intolerance develops primarily due to the absence of the enzyme lactase and can occur in childhood or adulthood. Treatment involves ingestion of commercially-available lactase enzyme preparations often produced by *Aspergillus* bacteria [1, 2]. Although anaphylactic reactions involving IgE mediated hypersensitivity have been reported for a number of foods, this case report represents the first documented evidence of anaphylaxis after exposure to supplemental lactase enzyme preparation.

**Fig. 3:**
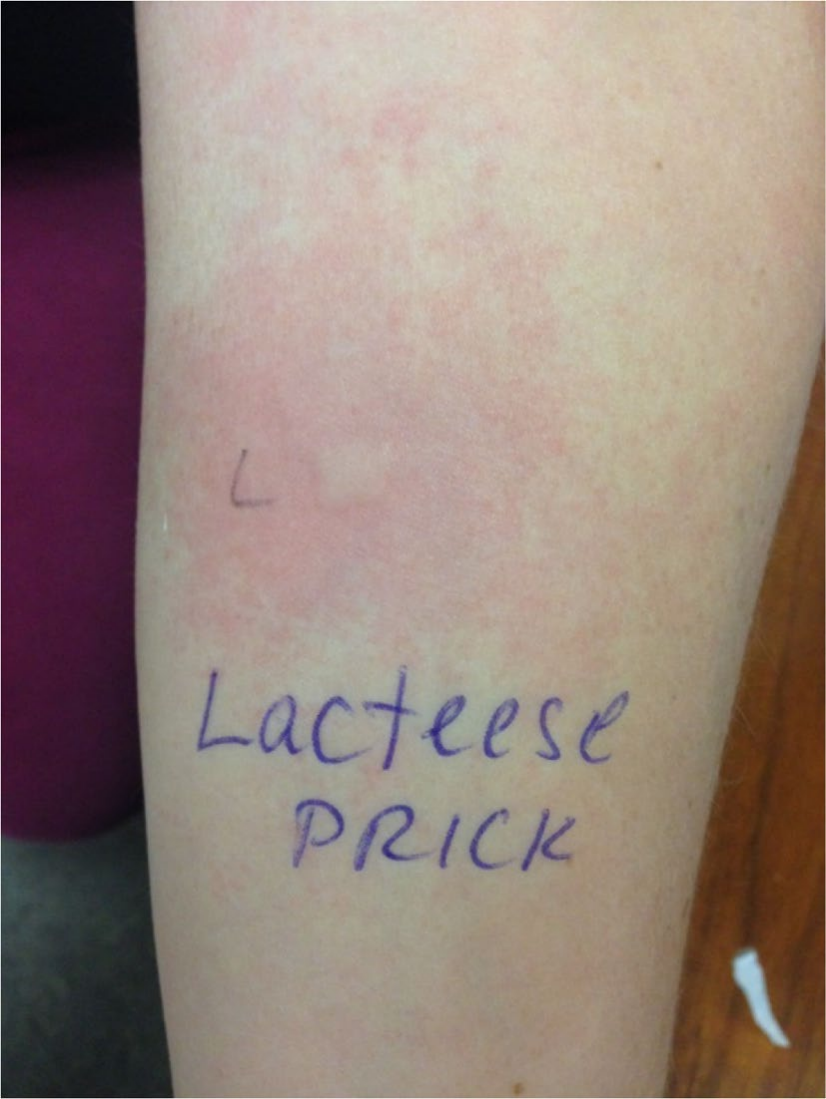
Skin test response to lactase tablet allergen (brand name Lacteese) applied to patient’s forearm

**Case report:** A 38 year old white female teacher presented with a history of adult-onset lactose intolerance and a suspected allergy to lactase tablets after an episode of bilateral orbital swelling, shortness of breath, and throat constriction that responded to diphenhydramine. The patient’s past medical history included oral allergy syndrome and medication-controlled asthma. She handled lactase tablets for years due to her children being lactose intolerant but only recently began using the tablets herself. Physical examination was benign, and skin prick testing to a slurry of the lactase tablet revealed a strongly positive reaction wheal size of 10 mm and flare of 60 mm with normal controls (Fig. 3). The patient required cetirizine treatment in clinic. Skin testing was performed with individual ingredients of the lactase tablet provided by the manufacturer and *Aspergillus niger*, a common bacteria used in lactase preparations. Only concentrated lactase enzyme elicited a positive response. Avoidance of lactase tablets was advised and the patient was educated in the use of injectable epinephrine.

**Conclusion:** This is the first documented case report of an anaphylactic reaction to lactase enzyme. The patient experienced systemic symptoms including shortness of breath and orbital swelling, reinforcing the importance of education and avoidance of triggers in these rare circumstances.

**Disclosures:** None.

**Consent:** Written informed consent was obtained from the patient for publication of this case report and any accompanying images.

**References**

1. Heyman M. Lactose intolerance in infants, children, and adolescents. Am Acad Pediatr. 2006;118(3):1279–86.

2. Swagerty D et al. Lactose intolerance. Am Fam Physician. 2002;65:(9):1845–50.

## A20 Risk of solid tumor malignancies in patients with primary immune deficiency

### Kateryna Vostretsova^1^, Donald F Stark^2^

#### ^1^Internal Medicine, University of British Columbia, Vancouver, BC, V5R 3V8, Canada, ^2^Allergy and Immunology, University of British Columbia, Vancouver, BC, V5Z 1M9, Canada

##### **Correspondence:** Kateryna Vostretsova - katerynavostretsova@gmail.com

*Allergy, Asthma and Clinical Immunology* 2016, **12(Suppl 1)**:A20

**Background:** Primary immune deficiency (PID) is a major risk factor for the development of malignancy in both the pediatric and adult patient population. It is a major cause of morbidity and mortality, coming second only after infection as the leading cause of death. To date, lymphoreticular malignancies are cited as the most common type of malignancy known to affect people with PID. Little is known about the risk of solid tumor malignancies in this population.

**Methods:** A literature review and a retrospective chart review of 400 adult patients at a local private practice was undertaken. Patients were selected if they both met a diagnosis of primary immune deficiency and solid tumor malignancy. Solid tumor malignancy was defined as a malignancy that was neither lymphoma nor leukemia.

**Results:** Two patients met the criteria of PID and solid tumor malignancy. The first was a male with X-linked agammaglobulinemia (XLA) who developed colorectal cancer and the other patient was a female with Common Variable Immune Deficiency (CVID) who was diagnosed with pancreatic cancer.

**Conclusion:** There are only a few studies in the literature regarding the incidence of solid tumor malignancies and PID. These two cases add additional evidence that patients with PID may not only be at risk for lymphoproliferative tumors but perhaps of solid tumors as well. The diagnosis of PID should alert the clinician to this potential relationship and ensure patients with PID undergo age appropriate primary prevention strategies. Additional studies are required to validate these conclusions.

**References**

1. Aghamohammadi A, Rezaei N, Gharagozlou M. Hodgkin lymphoma in two siblings with common variable immunodeficiency. Pediatr Hematol Oncol. 2007;24:337–42.

2. Leechawengwongs E, Shearer W. Lymphoma complicating primary immunodeficiency síndromes. Curr Opin Hematol. 2012;19:305–12.

3. Salavoura K, Kolialexi A, Tsangaris G, Mavrou A. Development of cáncer in patients with primary immunodeficiencies. Anticancer Res. 2008;28:1263–9.

4. Van Der Meer J, Weening R, Schellekens P, Munstar I, Nagengast F. Colorectal cáncer in patients with X-linked agammaglobulinaemia. Lancet. 1993;341:1439–40.

## A21 Is it time to adopt the chromogenic assay for measuring C1 esterase inhibitor function in patients with HAE type 2?

### Elizabeth Yeboah^1^, Paul K. Keith^1^

#### ^1^Division of Clinical Immunology and Allergy, Department of Medicine, McMaster University, Hamilton, ON, L8S 4K1, Canada

##### **Correspondence:** Paul K. Keith - keithp@mcmaster.ca

*Allergy, Asthma and Clinical Immunology* 2016, **12(Suppl 1)**:A21

**Background:** Type II HAE is characterized by functional deficit in C1 inhibitor (C1 INH). C1 INH function can be assessed by radial immunodiffusion (RID), ELISA or a chromogenic assay [1]. We present our institution’s experience with a type II HAE patient, whose disease was initially undetected by radial immunodiffusion.

**Case presentation:** A 62-year-old female was referred to the Clinical Immunology Clinic, McMaster University for assessment of type II HAE. This diagnosis was made two decades prior when she had recurrent abdominal pain and a functional C1 INH deficit detected at a US laboratory. She had no history of facial, extremity or airway angioedema. She was previously managed with danazol prophylaxis, which was discontinued 15 years prior, and had never required C1 INH infusions. There was no family history of HAE. Co-morbidities included allergic rhinitis.

Seven months after consultation, while travelling abroad, she experienced a typical HAE flare with facial and airway swelling. She was managed effectively with repeated frozen plasma transfusions. Danazol prophylaxis was restarted. Upon return to Canada, she had three additional flares, managed with C1 INH infusions or icatibant.

Her HAE diagnosis was re-evaluated repeatedly (Table 4). C4 levels were consistently low while C1 INH levels were high. Functional assay was normal twice, abnormal once and then normal again, as measured by radial immunodiffusion at the Clinical Chemistry and Immunology Laboratory, Hamilton Health Sciences. For her most recent evaluation, C1 INH function was concurrently measured by the chromogenic assay at CHU Sainte-Justine, Montreal, QC and found to be low.

**Table 4:** Patient results

Date	C4 complement (g/L)	C1 INH level (g/L)	C1 INH function (RID)	C1 INH function (chromogenic assay) (U/mL)
May 2014	0.11 (low)	0.67 (high)	Normal	
June 2014	0.11 (low)	0.81 (high)	Normal	
March 2015	0.11 (low)	0.61 (high)	Abnormal	
April 2015	0.11 (low)	0.56 (high)	Normal	0.08 (low)

**Conclusions:** A recent paper [1] suggests the chromogenic assay is more sensitive for diagnosing C1 INH dysfunction. This case demonstrates radial immunodiffusion’s inaccuracy in diagnosis of a patient with type II HAE. We suggest this method be replaced by the chromogenic assay which detected the diagnosis in our patient.

**Consent:** Written informed consent was obtained from the patient for publication of this case report and any accompanying images.

**Reference**

1. Li H, Busse P, Lumry W. Comparison of chromogenic and ELISA functional C1 inhibitor tests in diagnosing hereditary angioedema. J Allergy Clin Immunol Pract. 2015;3:200–5.

## A22 Emergency department visits for anaphylaxis and allergic reactions

### Michelle Martin-Rhee^1^, Cheryl Gula^1^, Clare Cheng^1^ and Geoff Paltser^1^

#### ^1^Canadian Institute for Health Information, Toronto, Ontario, M2P 2B7, Canada

##### **Correspondence:** Michelle Martin-Rhee - mmartin-rhee@cihi.ca

*Allergy, Asthma and Clinical Immunology* 2016, **12(Suppl 1)**:A22

**Background:** An estimated 2.5 million Canadians (approximately 7 %) report living with at least one food allergy. Some experts suggest this number is increasing. Epinephrine use and emergency medical services are recommended at the first sign of allergic reactions or anaphylaxis, yet little is known about the utilization of these resources for allergies in Canada. This study examines emergency department (ED) visits for anaphylaxis and allergic reactions and epinephrine auto-injector prescriptions.

**Methods:** Using data from CIHI’s National Ambulatory Care Reporting System, this analysis examines unscheduled ED visits that occurred between 2006/07 and 2013/14 with a diagnosis of anaphylaxis or allergic reaction. In addition, claims for epinephrine auto-injectors were estimated between 2006/07 and 2013/14 using data from the National Drug Utilization Information System. The analysis describes the patients who use the ED for anaphylaxis or allergic reaction and looks at trends over time in ED use as well as in prescriptions for epinephrine auto-injectors.

**Results:** About 1 % of total ED visits were for anaphylaxis or allergic reaction. Children and residents of rural areas were over-represented. Most visits occurred in the summer months. Since 2006–07, the rate of all allergy-related ED visits increased by 6 %, with increases particularly high for children under 18 (29 %) and for anaphylaxis-specific visits (80 %). At the same time, epinephrine auto-injector prescriptions increased by 64 %, again particularly among children.

**Conclusions:** While overall anaphylaxis and allergy rates remained stable at 1 %, the proportion of visits specifically for anaphylaxis increased significantly over time. In addition, an increased number of children are receiving prescriptions for epinephrine auto-injectors. Given the small increase in overall allergy-related visits, this may reflect a growing awareness of allergies in Canada and changes in coding practices. ED visits remain uncommon and rates of epinephrine auto-injector prescriptions are well below the estimated 7 % of Canadians with allergies.

## A23 START: Susceptibility to food allergies in a registry of twins

### Alizée Dery^1^, Ann Clarke^2^, Kari Nadeau^3^, Laurie Harada^4^, Kimberley Weatherall^5^, Celia Greenwood^9,10^, Denise Daley^6^, Yuka Asai^7^, Moshe Ben-Shoshan^8^

#### ^1^Division of Clinical Epidemiology, Department of Medicine, McGill University Health Center, Montreal, QC, Canada, ^2^Division of Rheumatology, Department of Medicine, University of Calgary, Calgary, AB, Canada, ^3^Department of Pediatrics, Division of Allergy, Immunology, and Rheumatology, Stanford University School of Medicine, Stanford, CA, USA, ^4^Anaphylaxis Canada, North York, ON, Canada, ^5^Multiple Births Canada, Orléans, ON, Canada, ^6^Department of Medicine, University of British Columbia, Vancouver, BC, Canada, ^7^Division of Dermatology, Department of Medicine, Queen’s University, Kingston, ON, Canada, ^8^Division of Allergy and Clinical Immunology, Department of Pediatrics, McGill University, Montreal, QC, Canada, ^9^Lady Davis Institute, Jewish General Hospital, Montreal, QC, Canada, ^10^Departments of Oncology, Epidemiology, Biostatistics and Occupational Health, and Human Genetics, McGill University, Montreal, QC, Canada

##### **Correspondence:** Alizée Dery - alizee.dery@mail.mcgill.ca

*Allergy, Asthma and Clinical Immunology* 2016, **12(Suppl 1)**:A23

**Background:** The role of gene-environment interactions in the pathogenesis of food allergy is currently unknown.

Twins provide an invaluable model for genetic studies, allowing the separation of genetic from environmental factors. This study aims to compare the proband concordance for food allergy in monozygotic (MZ) and dizygotic (DZ) twins.

**Methods:** Twins, where at least one member of the pair had a food allergy, were recruited from: Anaphylaxis Canada, Multiple Births Canada and the allergy clinic at the Montreal Children’s Hospital. Food allergy was diagnosed based on a convincing history of food- induced allergy in the presence of a positive skin prick test, or an IgE level of at least 0.35 kU/L as well as physician’s report of food allergy. The proband concordance [(twice number concordant pairs)/(twice number of concordant pairs + number of non-concordant pairs)] was compared in MZ and DZ twins for peanut and tree-nut allergies.

**Results:** Among four pairs of MZ twins and seven pairs of DZ twins for peanut allergy, the concordance rate was 0.0 (95 % CI 0.00, 0.60) and 0.60 (95 % CI 0.27, 0.86) respectively [difference = 0.6 (95 % CI 0.12, 1.00)].

Among four pairs of both MZ and DZ twins for cashew/pistachio allergy, the concordance rate was 0.40 (95 % CI 0.07, 0.82) and 0.66 (95 % CI 0.24, 0.94) respectively [difference = 0.26 (95 % CI −0.488, 1.00].

Among one pair of MZ twins and four pairs of DZ twins for pecan/walnut allergy, the concordance rate was 0.0 (95 % CI 0.0, 0.94) and 0.66 (95 % CI 0.24, 0.94) respectively [difference = 0.66 (95 % CI −0.29, 1.00].

**Conclusion:** The higher concordance rate in DZ twins for peanut allergy suggests that non-genetic factors play a major role in the pathogenesis of peanut allergy. The small sample size renders our comparisons for tree nuts inconclusive. Comparisons for egg, milk, shellfish, and fish were not performed due to an insufficient sample.

## A24 Qualifying the diagnostic approach employed by allergists when managing patients with self-diagnosed non-celiac gluten sensitivity (NCGS)

### Lee Horgan^1^, Teresa Pun^2^

#### ^1^Department of Postgraduate Medicine, Memorial University of Newfoundland, St. John’s, Newfoundland and Labrador, Canada, ^2^Uptown Health Centre, Toronto, Ontario, Canada

##### **Correspondence:** Lee Horgan - leehorgan@gmail.com

*Allergy, Asthma and Clinical Immunology* 2016, **12(Suppl 1)**:A24

**Background:** Non-celiac gluten/wheat sensitivity (NCGS) is an emerging entity in the medical literature and lay press. Characterized by nonspecific gastrointestinal symptoms that improve with gluten restriction, it often remains a self-reported illness [1]. This population represents a common and often challenging clinical encounter for practicing allergists.

**Method:** Canadian allergists were identified using publically available physician registries maintained by each province’s College of Physicians and Surgeons. A letter of information was faxed to eligible participants and interested parties were invited to complete a brief, semi-structured telephone interview discussing their clinical approach to NCGS.

**Preliminary Results:** A letter of information was faxed to the office of 55 Canadian allergists. At the time of abstract submission, two interviews were completed and three were scheduled for a later date. One respondent declined participation. Of the completed interviews, 50 % of participants were female with an average participant age of 36 years and an average time in practice of 27 months. Both respondents endorsed routine use of wheat allergy skin testing but varied in their use of Celiac Disease serology. One endorsed routine referral to gastroenterology for consideration of endoscopy. Follow-up practices also varied with one participant endorsing routine follow up at six months and the other offering follow-up quite infrequently.

**Conclusions:** Our desired sample size of 15-20 participants will allow us to document the Canadian allergist approach to NCGS. We intend to use this data to develop clinical care guidelines for NCGS in collaboration with our Canadian allergist colleagues.

**References**

1. Fasano A, Sapone A, Zevallos S, Schuppan D: Nonceliac gluten and wheat sensitivity. Gastroenterol. 2015;148:1195–1204.

## A25 Retrospective analysis on the agreement between skin prick test and serum food specific IgE antibody in adults with suspected food allergy

### Ling Ling^1^, Maria B. Ospina^2^, Kyriaki Sideri^1^, Harissios Vliagoftis^1^

#### ^1^Department of Medicine, University of Alberta, Edmonton, AB, T6G 2S2, Canada, ^2^Alberta Health Services, Respiratory Health Strategic Clinical Network, Edmonton, AB, T6G 2G3, Canada

##### **Correspondence:** Ling Ling - lling@ualberta.ca

*Allergy, Asthma and Clinical Immunology* 2016, **12(Suppl 1)**:A25

**Background:** Food allergy is increasingly common in adults [1]. Barriers to performing food challenges, the gold standard diagnostic test, have increased reliance on skin prick test (SPT) and food specific IgE to adjunct clinical history in diagnosing food allergy [2]. In adults, literature is scarce on the rate of agreement between these two tests. The objective of this study was to investigate the degree of concordance between SPT and specific IgE in patients with suspected food allergy.

**Methods:** A retrospective chart review was performed on adult patients seen in the University of Alberta Allergy Clinic between October 2013 and April 2015. Demographic and historical data were extracted, along with SPT results and food specific IgE titre. SPT wheal size >3 mm and specific IgE titre >0.35 kU/L were considered positive.

The agreement between SPT and specific IgE for individual food allergens was analyzed using Kappa coefficients [3].

**Results:** In the study period, 260 patients presented with a complaint of food reaction. There were 29.6 % males and 70.4 % females with a mean age of 38.8 years. Peanuts and tree nuts, followed by shellfish and fish were the most commonly suspected allergens. Most frequently tested allergens were peanut, hazelnut and almond. Agreement between SPT and specific IgE result was moderate for peanut (κ = 0.53, N = 87) and poor for both hazelnut (κ = 0.14, N = 60) and almond (κ = 0.11, N = 60). Kappa analysis for other food allergens is ongoing.

**Conclusion:** Initial analysis suggests that agreement between SPT and specific IgE for commonly suspected food allergens is not strong. This study therefore highlights the central role that clinical history must continue to play in the diagnosis of food allergy, and suggests a greater role for food challenges in the diagnostic algorithm.

**References**

1. Sicherer SH, Sampson HA. Food allergy: epidemiology, pathogenesis, diagnosis, and treatment. J Allergy Clin Immunol. 2014;133: 291–307.

2. National Institutes of Health, Department of Health and Human Services of the United States, National Institute of Allergy and Infectious Diseases: Guidelines for the Diagnosis and Management of Food Allergy in the United States, Summary of the NIAID Sponsored Expert Panel Report. 2010.

3. Landis JR, Koch GG. The measurement of observer agreement for categorical data. Biometrics. 1977;33:159–74.

## A26 Staple food hypersensitivity from infancy to adolescence: a report from the BAMSE cohort

### Jennifer L. P. Protudjer^1,2^, Mirja Vetander^1,2,3^, Marianne van Hage^1,4^, Ola Olén^3,5^, Magnus Wickman^1,2,3^, Anna Bergström^1,2^

#### ^1^Centre for Allergy Research, Karolinska Institutet, 171 77, Stockholm, Sweden, ^2^Institute for Environmental Medicine, Karolinska Institutet, 171 77, Stockholm, Sweden, ^3^Sachs’ Children and Youth Hospital, Södersjukhuset, 118 83, Stockholm, Sweden, ^4^Clinical Immunology and Allergy Unit, Department of Medicine Solna, Karolinska Institutet and University Hospital, 171 77, Stockholm, Sweden, ^5^Clinical Epidemiology, Karolinska Institutet, 171 76, Stockholm, Sweden

##### **Correspondence:** Jennifer L. P. Protudjer - jennifer.protudjer@ki.se

*Allergy, Asthma and Clinical Immunology* 2016, **12(Suppl 1)**:A26

**Background:** Hypersensitivity to the staple foods, milk, egg and wheat, has been described through childhood. We aimed to describe staple food hypersensitivity (sFHS), including symptoms and sensitisation by allergen-specific Immunoglobulin E (IgE) in a population-based birth cohort of Swedish children born in 1994–96 and followed to 16 years.

**Methods:** Using questionnaire and clinical data, we defined sFHS by 2 years of age, between 2–4 years of age and at 16 years of age. Symptoms included vomiting, diarrhoea, eczema/rash, itch, angioedema, rhinitis and/or asthma after staple food consumption. At 1, 2, 4, and 16 years, parent-reported doctor’s diagnosis of food allergy was ascertained. In a subgroup, sera obtained at 4 and 16 years were analysed for IgE to common food allergens (including staple foods) via fx5^®^. IgE reactivity to any or all staple foods was defined as IgE ≥ 0.35 kU_A_/l. From these data, we created three definitions: *A:* Symptoms only (N = 2386); *B:* Symptoms + doctor-diagnosed food allergy (N = 2386), and; *C:* Symptoms + IgE reactivity (N = 1493). Persistent sFHS was defined as sFHS by 2 years of age and/or between 2–4 years, and at 16 years. Descriptive statistics were used to establish prevalences and to test for differences.

**Results:** For *definitions A* and *B*, the prevalence of sFHS remained stable from 2 years, to 2–4 years, but increased significantly to 16 years (*A*: 6.3, 4.9, 7.2 %, respectively, p < 0.001; *B*: 3.7, 2.4, 6.3 %, respectively, p < 0.001). For *definition C*, the prevalence of sFHS at 4 and 16 years was 4.2 and 2.7 %, respectively (p < 0.04).

All children with persistent sFHS, regardless of definition, had symptoms, including more severe symptoms, compared to significantly fewer children with the same definition of sFHS at 16 years only (e.g. *definition A*: 83.3 %).

**Conclusion:** There is a discrepancy in the prevalence and symptoms of sFHS by definition. Persistent sFHS is associated with more severe symptoms.

**Funding:** JP is a postdoctoral researcher funded by the Karin and Sten Mörtstedt Initiative on Anaphylaxis.

## A27 Evaluating the impact of supervised epinephrine autoinjector administration during food challenges on perceived parent confidence

### Timothy Teoh^1^, Christopher Mill^2^, Tiffany Wong^2^, Ingrid Baerg^2^, Angela Alexander^2^, Kyla J. Hildebrand^2^, John Dean^2^, Boris Kuzeljevic^3^, Edmond S. Chan^2^

#### ^1^Faculty of Medicine, University of British Columbia, Vancouver, BC, V6T 1Z3, Canada, ^2^Division of Allergy and Immunology, Department of Pediatrics, BC Children’s Hospital, University of British Columbia, Vancouver, BC, V6H 3V4, Canada, ^3^Child and Family Research Institute, Vancouver, BC, V5Z 4H4, Canada

##### **Correspondence:** Timothy Teoh - tteoh@alumni.ubc.ca

*Allergy, Asthma and Clinical Immunology* 2016, **12(Suppl 1)**:A27

**Background:** Our previous work identified gaps in confidence for administering epinephrine autoinjectors among parents and children. The aim of this study is to further describe the impact of supervised autoinjector administration by parents/patients on confidence, knowledge and skill for future treatment of severe allergic reactions.

**Methods:** Patients aged 2–17 years with confirmed IgE-mediated food allergy at BC Children’s Hospital (2013–15) undergoing a physician supervised oral food challenge were recruited. A pre-challenge questionnaire validated by expert panel on patient and caregiver background information and self-assessed confidence (five point scale) in the ability to recognize and treat a severe allergic reaction was completed by each participant. If an autoinjector was deployed during the challenge, a similar questionnaire was administered. Since data was not normally distributed, Mann–Whitney U and Wilcoxon signed rank tests were used to test differences between groups.

**Results:** Among caregivers who never previously deployed an epinephrine autoinjector, 67/104 (66 %) experienced at least one severe allergic reaction within the family, and 25/104 (24 %) had experienced three or more severe reactions. Pre-challenge median scores for recognition of a severe reaction, confidence in autoinjector administration, knowledge of technique, and autoinjector skill were 4(IQR = 1), 3(IQR = 1), 4(IQR = 2), 3(IQR = 2). Further descriptive statistics are in Table 5. Parents who were health professionals perceived themselves to be more confident in all domains (median difference of 1 for each, p < 0.05) compared to non-health professionals. Post-challenge confidence in autoinjector administration and skill among those receiving epinephrine increased by a median of 1 and 2 respectively (p < 0.05).

**Table 5:** Descriptive statistics for pre-questionnaire variables (n = 128)

	%
Health professionals	21 %
English as a first language	70 %
Previous experience using autoinjector—at least once	18 % (23)
Most common foods challenged	Peanut (30 %), egg (28 %), tree nut (12 %)
Failed challenges	28 % (35) (9 patients required epinephrine)

**Conclusions:** Although participants described reasonable knowledge of recognition and technique, confidence and skill for autoinjector use were suboptimal. In those who used the autoinjector during an oral challenge, perceived confidence in administration and skill levels increased. This suggests potential educational benefit of supervised epinephrine self-treatment, since many who have never used an epinephrine autoinjector have experienced severe allergic reactions.

## A28 Local immunoglobulin production to *Aspergillus fumigatus* cystic fibrosis

### Jonathan Argeny^1^, Mia Gona-Hoepler^1^, Petra Fucik^1^, Edith Nachbaur^1^, Saskia Gruber^1^, Reto Crameri^2^, Andreas Glaser^2^, Zsolt Szépfalusi^1^, Claudio Rhyner^2^, Thomas Eiwegger^1^

#### ^1^Department of Paediatrics and Adolescent Medicine, Medical University of Vienna, Vienna, Austria, ^2^Swiss Institute of Asthma and Allergy Research, University of Zurich, Davos, Switzerland

##### **Correspondence:** Thomas Eiwegger - Thomas.eiwegger@meduniwien.ac.at

*Allergy, Asthma and Clinical Immunology* 2016, **12(Suppl 1)**:A28

**Background:** Cystic fibroses (CF) is an autosomal recessive multisystemical disorder that is characterized by a progressive neutrophil-dominated inflammation of the lungs and their colonization by typical bacteria. Patients suffering from CF are very often sensitized to *Aspergillus fumigatus* (*Asp fum*), an ubiquitously occuring fungus. In some cases, this can lead to the disease so called, allergic bronchopulmonary aspergillosis (ABPA).

We aimed to investigate whether local antibodies against *Asp fum* (IgG, IgA, IgE) in the lungs of children and adolescents with CF are detectable and if there is a correlation with reduced lung function.

**Methods:** BALF samples of children with CF (n = 49) and a matched control group (n = 12) were obtained by bronchoscopy and clinically relevant parameters [lung function, BMI, age, *Pseudomonas aeruginosa* (PA) colonization] were assessed. *Asp fum* -specific IgE was measured in serum using ImmunoCAP and *Aspergillus*-specific antibodies of different Immunoglobulin subclasses (IgG, IgA, IgE) were quantified in BALF using an “in house” ELISA.

**Results:** Local immunoglobulin production against *Asp fum* was observed in more than 80 % of CF patients compared to the control group. Specific AF-BAL IgE and systemic specific IgE did not correlate with each other in patients with CF (r = 0.26; p = 0.075). CF-patients with *Asp fum* in BALF had significantly increased *Asp fum*-IgG and *Asp fum*-IgA as compared to patients with no *Asp fum* detectable in BALF. Patients with *Asp fum*-IgE in BALF displayed a higher forced vital capacity as compared to patients with systemic and local IgE.

**Conclusions:** We provide evidence for local IgE production to AF in patients with CF. Local IgE may precede systemic *Asp fum*-Ige production or even have protective effects.

## A29 Extract consumption with skin prick test (SPT) devices

### Greg. Plunkett^1^, Brad Mire^1^

#### ^1^ALK, Round Rock, TX, 78664, USA

##### **Correspondence:** Greg. Plunkett - greg.plunkett@alk.net

*Allergy, Asthma and Clinical Immunology* 2016, **12(Suppl 1)**:A29

**Background**: Allergy skin prick testing involves introducing a small amount of allergen extract percutaneously using pointed devices. The current standard of care involves placing a drop of extract (40 μL) on the skin and then puncturing the skin through the drop via a prick lancet. Alternative methods include dipping sterile devices into extract and then pressing the device onto the skin. The objective of this study was to determine the amount of extract used for four devices at different tube/well fill volumes.

**Methods**: Duotip-test II^®^, Unitest PC^®^, Multi-test II^®^ and Multi-test PC^®^, and Prick Lancetter™ devices were studied. For Duotip-test II^®^, gravimetric volume by weight was determined by weighing one device before and after placing into a Dipwell^®^ tray/well containing 50 % glycerin. Twenty-five volumetric measurements were made for each device by dipping 5 devices 5 times and then measuring the remaining volume with a pipette. Low (0.3 mL), medium (0.6 mL) and high (1.0 mL) fill volumes were studied.

**Results**: Results are shown in Table 6. Volume depletion depended on how much extract was in the tube/wells.

**Conclusion**: Use of single and multiple test devices, tray and wells resulted in significantly more tests per vial (486–1923 tests) than the “prick and wipe” device (Prick Lancetter™, 143 tests) which may significantly reduce extract costs. A comparison of device sterility, reproducibility, speed of application and patient pain requires further investigation.

**Table 6:** Volume depletion per test for allergy skin test devices

	0.3 mL			1.0 mL		
	µL/Test	% CV	Tests/5 mL	µL/Test	% CV	Tests/5 mL
Duotip-test II^®^-gravimetric	2.89	15.6	1730	9.09	8.3	550
Duotip-test II^®^-volumetric	2.60	3.8	1923	9.15	0.9	546
Unitest PC^®^-gravimetric	10.22	8.4	489	20.10	4.9	249
Unitest PC^®^-volumetric	10.28	3.2	486	21.20	0.8	236
Multi-test II^®^-volumetric	7.15	3.3	699	9.92	6.9	504
Multi-test PC^®^-volumetric	8.02	1.5	623	13.82	3.2	362
Prick Lancetter™-drop	35	9.4	143	35		143

## A30 Evaluation of our cases with nonsteroidal anti-inflammatory drug reactions

### Mehtap Yazicioglu^1^, Ceren Can^1^, Gokce Ciplak^2^

#### ^1^Department of Pediatric Allergy, Trakya University, 22030, Edirne, Turkey, ^2^Department of Pediatrics, Trakya University, 22030, Edirne, Turkey

##### **Correspondence:** Mehtap Yazicioglu - yazicioglu@superonline.com

*Allergy, Asthma and Clinical Immunology* 2016, **12(Suppl 1)**:A30

**Background:** Nonsteroidal anti-inflammatory drugs (NSAIDs) are very commonly used drugs worldwide, constituting the second major cause of hypersensitivity reactions after beta lactam antibiotics. The study’s aim was to evaluate clinical and demographic data of patients referred to our hospital with suspected hypersensitivity to NSAIDs.

**Method:** All cases with NSAID reaction history admitted to our pediatric allergy department between February 2013–2015 were included in our study. Case data was compiled by ENDA questionnaires and face-to-face interviews with parents. Skin tests and/or oral provocation tests (OPT) were performed with NSAIDs according to guidelines, after obtaining written informed parental consent.

**Results:** 50 cases (40 % male, 60 % female; mean age 7.36 ± 4.72 years) were included. Two patients (4 %) had asthma, rhinosinusitis, and nasal polyps, while six (12 %) had allergic underlying diseases—atopic dermatitis/asthma/allergic rhinitis.

The drugs considered to have caused the symptoms were acetaminophen in 18 (36 %) cases, ibuprofen in 17 (34 %), acetaminophen and ibuprofen in 11 (22 %), acetylsalicylic acid (ASA) in 2 (4 %), naproxen in 1 (2 %), and acetaminophen, naproxen and ASA in one (2 %).

Presenting complaints were urticaria in 12 (24 %) cases, angioedema in 5 (10 %), urticaria and angioedema in 13 (26 %), maculopapular/morbilliform rash in 18 (36 %), and anaphylaxis in 2 (4 %). Depending on the latency between administration of the NSAID and clinical symptoms, reactions were immediate in 31 (62 %) patients, delayed in 19 (38 %).

OPT were performed in 17 cases, reporting immediate reactions with NSAIDs, resulting positive in three cases with acetaminophen and two cases with ibuprofen. In two delayed reaction to NSAID cases, ibuprofen patch testing was performed, followed by OPT, both resulting negative.

**Conclusions:** Skin and/or challenge tests were performed in 38 % of cases reporting reactions to NSAIDs. 26 % confirmed as NSAID hypersensitivity. In other cases, infections, food, and additives might cause symptoms.

## A31 Reasons for referral and final diagnoses in a tertiary care pediatric allergy clinic

### Victoria E. Cook^1^, Kyla J. Hildebrand^1^, Elodie Portales-Casamar^1^, Christopher Mill^1^, Edmond S. Chan^1^

#### ^1^Department of Pediatrics, Division of Allergy and Immunology, University of British Columbia, Vancouver, Canada

##### **Correspondence:** Victoria E. Cook - Victoria.Cook@cw.bc.ca

*Allergy, Asthma and Clinical Immunology* 2016, **12(Suppl 1)**:A31

**Background:** With the burden of allergic disease increasing worldwide [1], prolonged wait times are a significant concern. Published reviews of referral patterns have focused on adult populations and do not report final diagnoses [2, 3]. This is the first study that we are aware of to compare reasons for referral with final diagnoses from a pediatric allergy clinic.

**Methods:** A review of first presentations to the BC Children’s Hospital Allergy clinic is currently underway. Data on age, gender, referral source, reason for referral, and final diagnoses were recorded. Descriptive statistics were used to characterize reasons for referral and final diagnoses.

**Results:** Among 68 referrals reviewed to date, 78 % were from general practitioners, while 22 % came from specialists. Median patient age was 4 years (IQR4), and 51 % were female. Most had a single reason for referral (84 %). Table 7 shows referral indications and final diagnoses. Food allergy was the most common reason for referral (71 %), followed by allergic rhinoconjunctivitis (22 %), asthma (10 %), drug allergy (7 %), parent request (1 %), and laboratory result (1 %). Multiple diagnoses were assigned in 10 %. Food allergy was the most common final diagnosis (35 %), followed by allergic rhinoconjunctivitis (24 %), asthma (4 %), drug allergy (1 %) and food protein induced enterocolitis (1 %). Among those referred for suspected food allergy, only 50 % had a final diagnosis of food allergy. Nearly half (44 %) of all referrals received a non-allergic diagnosis.

**Table 7:** Reasons for referral and final diagnoses in a pediatric allergy clinic

Reason for referral (N)	Food allergy diagnosis N (%)	Asthma diagnosis N (%)	Allergic rhinoconjunctivitis diagnosis N (%)	Drug allergy diagnosis N (%)	Food protein induced enterocolitis diagnosis N (%)	No allergic diagnoses N (%)
Food allergy (48)	24 (50)	0 (0)	8 (13)	0 (0)	1 (2)	24 (50)
Asthma (8)	1 (13)	3 (38)	1 (13)	0 (0)	0 (0)	4 (50)
Allergic rhinoconjunctivitis (16)	2 (13)	1 (6)	8 (50)	0 (0)	0 (0)	8 (50)
Drug allergy (5)	0 (0)	1 (20)	1 (20)	1 (20)	0 (0)	3 (60)
Parent request (3)	0 (0)	1 (33)	1 (33)	0 (0)	0 (0)	2 (66)
Laboratory result (1)	0 (0)	0 (0)	1 (100)	0 (0)	0 (0)	0 (0)

**Conclusions:** We report a higher proportion of referrals for food allergy (71 %) than seen in prior reports from mixed adult/pediatric practices (19–24 %) [2, 3]. Only half of the patients referred for food allergy received a confirmed diagnosis, suggesting many may not have required referral. Greater efforts to educate parents and referring physicians about when to truly suspect a food allergy and the pitfalls of food allergy testing are warranted.

**References**

1. Pawankar R. Allergic diseases and asthma: a global public health concern and a call to action. World Allergy Organ J. 2014;7:1–3.

2. Levy ML, Walker S, Woods A, Sheikh A. Service evaluation of a UK primary care-based allergy clinic: quality improvement report. Nat Publ Group. 2009;18:313–9.

3. Ferre-Ybarz L, Salinas Argente R, Gomez Galan C, Nevot Falco S, Franquesca Rabat J, Trape Pujol F, Oliveras Alsina P, Pons Serra M. Analysis of changes in first allergy consultations over a period of 5 years. J Investig Allergol Clin Immunol. 2014;24:352–70.

## A32 Internist referral practices for inpatients with self-reported penicillin allergies at a tertiary care teaching hospital

### Michael N. Fein^1^, Emil P. Nashi^1^

#### ^1^Department of Clinical Immunology and Allergy, McGill University, Montreal, QC, H3G 1A4, Canada

##### **Correspondence:** Michael N. Fein - Michael.fein@mail.mcgill.ca

*Allergy, Asthma and Clinical Immunology* 2016, **12(Suppl 1)**:A32

**Background:** Unverified, self-reported penicillin allergy is a common clinical problem among hospitalized patients. These patients often receive broader spectrum, more expensive, and often inferior antibiotics secondary to their unverified penicillin allergy. The large majority of patients with self-reported allergy to penicillin do not have a true type-I mediated penicillin allergy and could benefit from de-labelling.

**Methods:** We conducted a retrospective cohort study of 575 consecutive patients admitted to the internal medicine ward at the Montreal General Hospital between April 1st, 2014 and September 9th, 2014. Each patient’s electronic chart was reviewed for documentation of reported penicillin allergy. For patients with reported penicillin allergy, their full chart was reviewed to determine what their documented drug reaction was, if they had received antibiotics during their admission, the indication for antibiotic use, antibiotics received, and presence or absence of inpatient or outpatient allergy consultation for evaluation.

**Results:** A total of 74 patients out of the 575 total admissions during the study period reported a penicillin allergy, with a prevalence of 12.9 %. Forty patients (54 %) with an unverified penicillin allergy received antibiotics during their hospitalization. The most common antibiotics prescribed to these patients were moxifloxacin, ciprofloxacin, ceftriaxone, and vancomycin. The most common allergic reaction listed in the electronic medical record was *unknown* (46 %). Only four patients (5.4 %) received inpatient consultation with an allergist and all of these patients were thought to have suffered an allergic reaction during their hospitalization. There were zero outpatient referrals made for hospitalized patients with a history of unverified penicillin allergy.

**Conclusion:** Referral rates for penicillin allergy evaluation continue to be extremely low—5.4 % in our study. Given the potential impact de-labelling programs may have on antibiotic utilization and stewardship, educational programs and de-labelling projects are currently being explored.

**References**

1. Harris AD, Sauberman L, Kabbash L, Greineder DK, Samore MH. Penicillin skin testing: a way to optimize antibiotic utilization. Am J Med. 1999;107(2):166–8.

2. CDC get smart for healthcare. http://www.cdc.gov/getsmart/healthcare/resources/slides/getsmart-healthcare.pdf.

3. Choosing wisely: ten things physicians and patients should question. http://www.choosingwisely.org/doctor-patient-lists/american-academy-of-allergy-asthma-immunology/.

4. Macy E. Penicillin and beta-lactam allergy: epidemiology and diagnosis. Curr Allergy Asthma Rep. 2014;14(11):476.

5. Sade K, Holtzer I, Levo Y, Kivity S. The economic burden of antibiotic treatment of penicillin-allergic patients in internal medicine wards of a general tertiary care hospital. Clin Exp Allergy. 2003;33(4):501–6.

6. Macy E, Contreras R. Health care use and serious infection prevalence associated with penicillin “allergy” in hospitalized patients: A cohort study. J Allergy Clin Immunol. 2014, 133(3):790–6.

7. Adkinson NF Jr, Thompson WL, Maddrey WC, Lichtenstein LM. Routine use of penicillin skin testing on an inpatient service. N Engl J Med. 1971;285(1):22–4.

8. Rimawi RH, Cook PP, Gooch M, Kabchi B, Ashraf MS, Rimawi BH, Gebregziabher M, Siraj DS. The impact of penicillin skin testing on clinical practice and antimicrobial stewardship. J Hosp Med. 2013;8(6):341–5.

9. Raja AS, Lindsell CJ, Bernstein JA, Codispoti CD, Moellman JJ. The use of penicillin skin testing to assess the prevalence of penicillin allergy in an emergency department setting. Ann Emerg Med. 2009;54(1):72–7.

10. Li M, Krishna MT, Razaq S, Pillay D. A real-time prospective evaluation of clinical pharmaco-economic impact of diagnostic label of ‘penicillin allergy’ in a UK teaching hospital. J Clin Pathol. 2014;67(12):1088–92.

## A33 Assessing the risk of reactions in children with a negative oral challenge after a subsequent use of amoxicillin

### Sofianne Gabrielli^1^, Christopher Mill^2^, Marie-Noel Primeau^1^, Christine Lejtenyi^1^, Elena Netchiporouk^1^, Alizee Dery^1^, Greg Shand^2^, Moshe Ben-Shoshan^1^

#### ^1^Division of Pediatric Allergy and Clinical Immunology, Department of Pediatrics, McGill University Health Center, Montreal, QC, Canada, ^2^Division of Clinical Epidemiology, Department of Medicine, McGill University Healthy Center, Montreal, QC, Canada

##### **Correspondence:** Sofianne Gabrielli - sofiannegabrielli@gmail.com

*Allergy, Asthma and Clinical Immunology* 2016, **12(Suppl 1)**:A33

**Background:** Graded drug challenge is used to determine amoxicillin allergy. However, the risk of future reaction upon subsequent treatment in those with a negative challenge is currently unknown. We aimed to assess the risk of amoxicillin hypersensitivity upon subsequent use in children referred due to suspected amoxicillin allergy who had a negative graded oral challenge.

**Methods:** All children referred to the Montreal Children’s Hospital for potential antibiotic allergy were recruited for the study between March 2013 and 2015. A standardized survey with questions on treatment, symptoms, and possible associated factors was filled out and an oral challenge (10 and 90 % of the oral dose) was conducted at the clinic visit. Six months after the challenge and every 6 months thereafter, the patients were contacted by phone to be asked questions on subsequent antibiotic use and associated reactions.

**Results:** Among 366 patients with a negative oral challenge eligible for a follow-up, 250 (68.3 %) patients responded. Of the contacted patients, 60 (24.0 %) reported amoxicillin use and 7 [11.7 % (95 % CI 5.2, 23.2 %)] patients reacted to a subsequent treatment. Amoxicillin was primarily used to treat acute otitis media (AOM).

Of those seven patients that did not tolerate the amoxicillin, 57.1 % were male with a median age of 0.9 years (IQR 0.5, 3.9) at the time of reaction and 2.1 years (IQR 1.8, 5.3) at the time of challenge. Sociodemographic characteristics and co-morbidities as well as personal and familial history of atopy were similar between respondents and non-respondents to follow-up and also between those that reacted and those that tolerated amoxicillin treatment (Table 8). All subsequent reactions presented as hives and 85.7 % (95 % CI 42.0, 99.2 %) developed in the first 3 days of treatment.

**Table 8:** Data collected at initial clinic visit and during follow-up

Variable (%, 95 % CI)	Used amoxicillin (N = 60)	Responders not using amoxicillin at follow-up (N = 190)	Non responders (N = 116)	Reacted to subsequent dose (N = 7)
Sex (males %)	(29 of 60) 48.3 (35.4, 61.5)	(101 of 190) 53.2 (45.8, 60.4)	(67 of 116) 57.8 (48.2, 66.8)	(4 of 7) 57.1 (20.2, 88.2)
Age at reaction (median, IQR = Interquartile range)	1.0 (0.4, 2.0)	1.2 (0.5, 3.0)	1.0 (0.5, 2)	0.9 (0.5, 3.9)
Age at challenge (IQR)	2.6 (1.6, 4.6)	4.2 (2.0, 7.9)	3.5 (2.1, 5.5)	2.1 (1.8, 5.3)
Time interval years between reaction and challenge (median, IQR)	1.1 (0.5, 2.1)	1.8 (1.0, 4.9)	1.7 (0.9, 3.3)	1.3 (0.8, 1.6)
Amoxicillin given for AOM (acute otitis media)	73.3 (60.1, 83.5)	66.3 (59.0, 72.9)	64.3 (54.8, 72.9)	42.9 (11.8, 79.8)
Characteristics of initial reaction				
Hives	75.0 (61.9, 84.9)	56.8 (49.5, 63.9)	62.9 (53.4, 71.6)	100 % (56.1, 100.0 %)
Macular/papular rash	16.7 (8.7, 29.0)	34.2 (27.6, 41.5)	31.0 (23.0, 40.4)	
Between 1–3 days	51.7 (38.5, 64.6)	52.1 (44.8, 59.4)	44.7 (35.5, 54.3)	28.6 (5.1, 69.7)
Between 4–7 days	20.0 (11.2, 32.7)	26.1 (20.1, 33.1)	33.3 (25.0, 42.9)	28.6 (5.1, 69.7)
>7 days	21.6 (12.5, 34.5)	14.7 (9.8, 20.4)	15.8 (9.9, 24.1)	28.6 (5.1, 69.7)
After treatment ended	3.3 (0.6, 12.5)	4.8 (2.4, 9.2)	2.6 (0.6, 8.1)	14.3 (0.7, 0.6)
Known asthma	8.5 (3.2, 19.4)	15.9 (11.1, 22.0)	16.8 (10.7, 25.3)	14.3 (0.7, 0.6)
Known eczema	23.7 (14.0, 36.9)	21.2 (15.7, 27.8)	23.0 (15.8, 32.1)	28.6 (5.1, 69.7)
Parental history of drug allergy	43.2 (27.5, 60.4)	26.5 (19.4, 35.0)	29.3 (20.0, 40.5)	50.0 (18.8, 81.2)
Parental history of asthma	29.3 (12.4, 41.6)	15.9 (10.3, 23.5)	9.8 (4.6, 18.8)	0

**Conclusion:** The risk of a subsequent reaction among those with a negative challenge is almost 10 %. All reactions were limited to the skin, but some of the reported skin reactions may have been triggered by viral infections and hence the risk may be an overestimate. Clinicians can rely on oral challenges to safely assess true allergy among children with suspected skin-related reactions to amoxicillin.

## A34 Validity of self-reported penicillin allergies

### Erica Hoe^1^, Joel Liem^2^

#### ^1^Schulich School of Medicine and Dentistry, Western University, Windsor, ON, N9B 3P4, Canada, ^2^Windsor Allergy Asthma Education Centre, Windsor, ON, N8X 2G1, Canada

##### **Correspondence:** Erica Hoe - ehoe2016@meds.uwo.ca

*Allergy, Asthma and Clinical Immunology* 2016, **12(Suppl 1)**:A34

**Background:** 10 % of people claim to have a penicillin allergy. Verifying a penicillin allergy is important because it reduces patient exposure to other antibiotics that can be associated with increased patient morbidity and healthcare costs. The aim of this study is to report the validity of self-reported penicillin allergies in patients presenting to an allergy clinic.

**Methods:** Retrospective chart review of 284 patients who presented to an allergy clinic between April 2011 to December 2014 for penicillin allergy assessment. Patients had undergone skin, intradermal testing of penicillin and a 5-day oral dose challenge of amoxicillin. Patients whose reactions were negative to skin and intradermal testing went on to receive a 5-day oral dose challenge of amoxicillin. Patients with history of serum sickness or Steven-Johnson syndrome/TENS were excluded from this clinic.

**Results:** 8.1 % (23 of 284) of self-reported penicillin allergies were found to be truly allergic as determined by either skin test, intradermal test or 5-day oral challenge. There were 6 reactions to skin and intradermal testing, and these patients did not go on to receive oral doses of amoxicillin. Of those who received the oral challenge, a small number of patients (5 of 284) reacted within 24 h of ingestion of the first dose of amoxicillin. A majority of patients (12 of 284) had a delayed reaction with a rash occurring 24 h after initial ingestion penicillin. Of the oral challenges, the reactions included 6 with hives and 11 with a rash. No oral challenge resulted in cardiopulmonary compromise.

**Conclusions:** Of those reporting a penicillin allergy, only 8.1 % were proven to have a reaction. Of these, the majority had a delayed reaction to penicillin, which typically only involved a rash. There were no life-threatening reactions. Penicillin allergy testing can determine whether patients should actually be avoiding penicillin.

## A35 Effectiveness of allergy-test directed elimination diets in eosinophilic esophagitis

### Jason K Ko^1^, David JT Huang^2^, Jorge A Mazza^2^

#### ^1^Schulich School of Medicine and Dentistry, University of Western Ontario, London, ON, N6A 5C1, Canada, ^2^Division of Allergy and Clinical Immunology, Department of Medicine, University of Western Ontario, London, ON, N6A 3K6, Canada

##### **Correspondence:** Jason K Ko - kko2017@meds.uwo.ca

*Allergy, Asthma and Clinical Immunology* 2016, **12(Suppl 1)**:A35

**Background:** Currently there is no consensus on the optimal treatment of eosinophilic esophagitis (EoE). One of the options, an elimination diet guided by allergy test results, has been shown to be effective to varying degrees with greater efficacy in pediatric cohorts [1, 2]. However, results in adults have been less promising as higher rates of negative allergy tests hinder efforts to guide the diet [3]. This study explores the results of allergy-test directed elimination diets in a group of patients (n = 16) treated at the Allergy and Immunology Clinic in London, Ontario.

**Methods:** A retrospective chart review was performed using a database of patients diagnosed with EoE (histology ≥ 15 eo/hpf in esophageal biopsy) and treated at the Allergy and Immunology Clinic at St. Joseph’s Hospital, London, ON. Patients were included in the review if they had been prescribed an elimination diet guided by allergy testing [skin-prick test (SPT) and/or atopy patch test (APT)], and had esophageal biopsies taken ≥ 8 weeks after initiation of the diet.

Pre-treatment eosinophil counts were recorded from peak eosinophil counts of the most recent biopsy prior to initiation of the elimination diet.

**Fig. 4:**
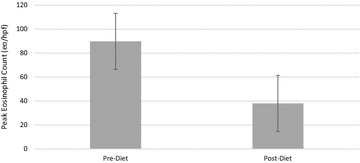
Average esophageal eosinophil level is decreased after initiation of elimination diet. *Error bars* represent standard error used in paired t test to compare the pre-diet and post-diet averages (p = 0.043)

**Results:** The average age of subjects at diet initiation was 43.75 years (see Table 9 for subject demographics). On average, each subject eliminated 1.7 foods from their diet. Post-diet biopsies displayed a significantly lower level of peak eosinophils/hpf compared to pre-diet biopsies on average (37.9 vs. 89.8, p = 0.043; Fig. 4). Three patients had post-diet biopsies below the histological threshold of diagnosis for EoE (<15 eo/hpf).

**Table 9:** Demographics and confounding treatments

Parameter	Value
N	16
# Male	8/16
Average age at diet initiation, years (range)	43.75 (2–65)
Average # foods eliminated (range)	1.7 (1–3)
Average # days to post-diet biopsy	192
Positive APT	15/16
Positive SPT	5/16
Corticosteroid use^a, b^	8/16
PPI use^a^	12/16

^a^ Considered to use if any prescriptions after EoE diagnosis

^b^ Only 1/4 of patients prescribed swallowed corticosteroids had used them in the month before the post-diet biopsy

**Conclusions:** This chart review shows that patients undergoing elimination diets directed by allergy tests have significant histological improvement after their diets. It must be noted that this review did not control for the effects of confounding treatments such as PPIs and corticosteroids, with the latter being of greater concern to histological results. Half of the subjects examined (8 out of 16) had been, at some time before the post-diet biopsy, prescribed swallowed corticosteroids for EoE. However, only 1/4 of steroid-prescribed subjects reported using the steroids during the 30-day period immediately prior to their post-diet biopsy.

**References**

1. Syrigou E, Angelakopoulou A, Zande M, Panagiotou I, Roma E, Pitsios C. Allergy-test-driven elimination diet is useful in children with eosinophilic esophagitis, regardless of the severity of symptoms. Pediatr Allergy Immunol. 2015;26:323–9.

2. Spergel JM, Brown-Whitehorn TF, Cianferoni A, Shuker M, Wang ML, Verma R, Liacouras CA. Identification of causative foods in children with eosinophilic esophagitis treated with an elimination diet. J Allergy Clin Immunol. 2012;130:461–7 e465.

3. Vernon N, Shah S, Lehman E, Ghaffari G. Comparison of atopic features between children and adults with eosinophilic esophagitis. Allergy Asthma Proc. 2014;35:409–14.

## A36 Allergy testing and dietary management in pediatric eosinophilic esophagitis (EoE): a retrospective review of a tertiary Canadian centre’s experience

### Mary McHenry^1^, Anthony Otley^1,3^,Wade Watson^1,2^

#### ^1^Department of Pediatrics, Faculty of Medicine, Dalhousie University, Halifax, NS, Canada, ^2^Division of Allergy, IWK Health Centre, Halifax, NS, Canada, ^3^Division of Gastroenterology, IWK Health Centre, Halifax, NS, Canada

##### **Correspondence:** Mary McHenry - jamiesonmary@gmail.com

*Allergy, Asthma and Clinical Immunology* 2016, **12(Suppl 1)**:A36

**Background:** Foods are a major trigger for eosinophilic esophagitis (EoE) in children; however, the optimal method for identifying the causative foods is unclear. We investigated the role of allergy testing in a pediatric tertiary centre and its use in identifying causative foods in children with biopsy-confirmed EoE.

**Methods:** A retrospective analysis of all children diagnosed with EoE by a pediatric gastroenterologist from January 2004 to September 2014 was completed. All patients met criteria for diagnosis defined by symptoms of esophageal dysfunction and biopsy-proven eosinophilia with greater than 15 eosinophils per high-power field (hpf).

**Results:** Ninety-eight (98) children were included (average age 10.4 years, 77 % male, and 61 % atopic). Presenting symptoms included dysphagia (52 %), abdominal pain (38 %), and GERD (35 %). 69 patients were assessed by an allergist, 62 had skin prick testing (SPT) to foods; 40 were positive, most commonly to egg, soy, peanut and rice. 50 had atopy patch testing (APT); 26 were positive, most commonly to chicken, egg, wheat and corn. 46 patients underwent dietary intervention as part of their management, with 27 patients (57 %) reporting symptom improvement. 14 patients had follow-up endoscopy and nine had documented histologic improvement. With dietary interventions, the average number of foods avoided was 2.7.

**Conclusions:** Approximately half of the EoE patients undergoing dietary modifications based on allergy testing reported symptom improvement. When compared to the Six-Food Elimination Diet (SFED), patients avoided on average fewer foods. A staged-approach using SPT and APT followed by SFED may be an option for some patients, with the advantage of potentially avoiding less foods. This benefit needs to be balanced against the time required for allergy testing. Further studies comparing the feasibility of these two dietary approaches in the management of EoE in children are needed.

## A37 Visualizing the impact of atopic and allergic skin disease

### Dominik A. Nowak^1^, John N. Kraft^2^

#### ^1^School of Medicine, McMaster University, Hamilton, ON, L8S 4L8, Canada, ^2^Lynde Institute for Dermatology, Markham, ON, L3P 1X2, Canada

##### **Corresopondence:** Dominik A. Nowak - dominik.nowak@medportal.ca

**Background:** Cutaneous disease is known to impact psychosocial wellbeing. This sentiment is especially true in the chronic inflammatory dermatoses such as atopic dermatitis and idiopathic urticaria. Degree of itch plays a role, as do age and socioeconomic status. There is poor correlation, nonetheless, between severity and impact of skin disease, and physicians are themselves poor at gauging the psychosocial impact of skin problems [1]. We set out to visualize this impact by means of a word cloud.

**Methods:** Participants answered the open-ended question “How has your skin affected you (e.g. your mood, self-image, relationships, discomfort)?” Responses were transcribed into the word cloud software Wordle [http://www.wordle.net].

**Fig. 5:**
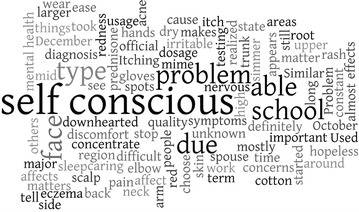
Word cloud depicting relative quantity of words used in response to the question, “How has your skin affected you (e.g. your mood, self-image, relationships, discomfort)?” Only inclusive of participants with atopic or allergic skin disease

**Results:** Eighty responses were recorded, with a mean age of 44 and a mean word count of 20. Twenty-five of 80 participants screened positive for depression by scoring three or above on the two-item PHQ-2 tool. Seven of eight participants whose complaints included the atopic or allergic screened positive—these included patients with atopic dermatitis, idiopathic urticaria, hand eczema, and allergic contact dermatitis. More frequent phrases were given a proportionally larger size in the graphic. Figure 5 visualizes the answers provided by the eight atopic and allergic patients.

**Conclusions:** Clinicians cannot overlook psychosocial wellbeing in skin disease. Specialists pressed for time may consider aligning themselves with a skin-emotion expert, be it a social worker or psychiatrist [2].

**References**

1. Fried RG, Gupta MA, Gupta AK. Depression and skin disease. Dermatol Clin. 2005;23(4):657–64.

2. Fried RG. Nonpharmacologic management of psychodermatologic conditions. Semin Cutan Med Surg. 2013;32(2):119–25.

## A38 Cystic fibrosis with and without nasal polyposis in pediatric patients: a cross-sectional comparative study

### Mihaela Paina^1^, Ahmed A. Darwish Hassan^2^, Delia Heroux^2^, Lynn Crawford^2^, Gail Gauvreau^2^, Judah Denburg^2^, Linda Pedder^1^, Paul K. Keith^2^

#### ^1^Department of Pediatrics, McMaster University, Hamilton, ON, L8S 4K1, Canada, ^2^Department of Medicine, McMaster University, Hamilton, ON, L8N 3Z5, Canada

##### **Correspondence:** Mihaela Paina - mihaela.paina@medportal.ca

*Allergy, Asthma and Clinical Immunology* 2016, **12(Suppl 1)**:A38

**Background:** Sinonasal disease is often diagnosed in patients with cystic fibrosis (CF). Some studies suggest that CF patients with nasal polyps have less severe pulmonary disease and overall improved survival. Our study examines factors associated with nasal polyposis in pediatric CF patients.

**Methods:** Cross-sectional study of 26 CF patients (2008–2011). Nasal polyps were diagnosed by anterior rhinoscopy. The study group (n = 16, 61.5 %): CF patients with nasal polyposis. The comparison group (n = 10, 38.5 %): CF patients without nasal polyposis. Clinical/biochemical data were gathered. Pulmonary function test, skin prick test, sputum/throat culture, nasal lavage and genotype analysis were performed. Minitab (*t* test, Chi square test) and a p value of < 0.05 were used for statistical analysis and significance, respectively.

**Results:** CF patients with nasal polyps are more likely to have Aspergillus serum precipitins (50 vs 10 %, p = 0.02). Positive allergy skin prick tests are common (50 vs 70 %, p = 0.29). Atopy to Aspergillus fumigatus is most prevalent in the nasal polyposis group (62.5 vs 42.85 %, p = 0.29). Other appreciable characteristics of this group are: better nutritional status (mean BMI 20.7 vs 18.7 kg/m^2^, p = 0.06), ΔF508 homozygosity (68.7 vs 40 %, p = 0.13), suboptimal vitamin D levels in winter (83.3 vs 50 %, p = 0.14) and better pulmonary function (mean FEV1 % predicted 82 vs. 78, p = 0.4). Staphylococcus aureus is the most common pathogen in the sputum/throat cultures and neutrophils are prevalent in all nasal lavage samples, with no difference between the groups.

**Conclusions:** Patients with CF and nasal polyposis may constitute a clinical subgroup within the spectrum of the disease. These patients appear to have a higher exposure to Aspergillus. This, together with the higher prevalence of atopy to Aspergillus, may suggest an association with nasal polyposis. Also more likely are: ΔF508/ΔF508 genotype, better pulmonary function**/**nutritional status, and lower vitamin D levels in winter. Our results confirm and complement those of previous studies.

**Acknowledgements:** This research study received funding from the Ontario Thoracic Society (Block Term Grant).

## A39 Evaluation of macrolide antibiotic hypersensitivity: the role of oral challenges in children

### Bahar Torabi^1^, Marie-Noel Primeau^1^, Christine Lejtenyi^1^, Elaine Medoff^1^, Jennifer Mill^1^, Moshe Ben-Shoshan^1^

#### ^1^Pediatric Allergy and Immunology, Montreal Children’s Hospital, McGill University, Montreal, QC, H4A 3J1, Canada

##### **Correspondence:** Bahar Torabi - bahar.torabi@mail.mcgill.ca

*Allergy, Asthma and Clinical Immunology* 2016, **12(Suppl 1)**:A40

**Background:** Studies suggest that up to 10 % of children develop rashes while on antibiotics for respiratory infections [1]. The majority are diagnosed without further evaluation and are labelled as allergic to the implicated antibiotic. There is sparse data regarding the percentage of established macrolide allergy among children who present with suspected macrolide hypersensitivity. Furthermore, there are no standardized skin tests to confirm the presence of macrolide hypersensitivity [2]. Our objective was to assess the risk of a hypersensitivity reaction through the use of a graded oral challenge in patients with a suspected macrolide allergy.

**Methods:** As part of the LAACTAM (β-LActam and other Antibiotics allergy in Children: Tests, assessment and Management) study, children presenting to the allergy clinic at the Montreal Children’s Hospital for the assessment of a potential macrolide antibiotic allergy were recruited. Data on demographics and reaction characteristics was collected through a standardized questionnaire. Patients underwent a graded challenge (10 and 90 % of the dose) of either azithromycin or clarithromycin, and were observed for 1 h after the last dose. In addition, patients were contacted every 12 months to query on potential non-immediate reactions or reactions on subsequent use.

**Results:** Fifty-one subjects with a macrolide allergy were recruited. 80.4 % (95 % CI 66.5, 89.7 %) had reported a reaction to clarithromycin and 19.6 % (95 % CI 10.3, 33.5 %) to azithromycin. The majority of reactions were isolated to the skin and occurred within the first 3 days of the antibiotic course (Table 10). Three subjects out of 51 (5.9 % [95 % CI 1.5, 17.2 %]) had a positive graded challenge. All three reactions were limited to the skin and occurred within the first hour of observation. No late reactions were documented and none of the patients reported repeated use of the culprit macrolide.

**Table 10:** Characteristics of macrolide hypersensitivity reactions in children

Age at time of reaction (median, IQR)	1.25 years (0.48, 3.09)
Gender (%)	
Male (95 % CI)	52.9 % (38.6, 66.8)
Symptoms	
Urticaria	62.7 % (48.1, 75.5)
Generalized pruritus	33.3 % (21.1, 48.0)
Maculopapular rash	25.5 % (14.8, 39.9)
Angioedema	15.7 % (7.5, 29.1)
Gastrointestinal	3.9 % (0.7, 14.6)
Throat tightness	2.0 % (0.1, 11.8)
Breathing difficulties	2.0 % (0.1, 11.8)
Arthritis/arthralgia	2.0 % (0.1, 11.8)
Time of reaction after 1st dose	
After 1–3 days	62.7 % (48.1, 75.5)
After 4–7 days	21.6 % (11.8, 35.7)
After >7 days	11.8 % (4.9, 24.6)
After treatment ended	2.0 % (0.1, 11.8)
Duration of reaction	
1–3 days	80.3 % (66.5, 89.7)
4–7 days	13.7 % (6.2, 26.8)
>7 days	5.8 % (1.5, 17.2)

**Conclusion:** The risk of a reaction in children with a history of a macrolide hypersensitivity is low and seems to occur within 1 h of the oral challenge. Currently an oral provocation is the best tool in diagnosing a macrolide allergy. Larger studies are needed to support these findings and identify risk factors associated with positive challenges.

**References**

1. Coker TR, Chan LS, Newberry SJ, Limbos MA, Suttorp MJ, Shekelle PG, Takata GS. Diagnosis, microbial epidemiology, and antibiotic treatment of acute otitis media in children: a systematic review. JAMA. 2010;304:2161–9.

2. Fernandez TD, Mayorga C, Ariza A, Corzo JL, Torres M. Allergic reactions to antibiotics in children. Curr Opin Allergy Clin Immunol. 2014;14:278–85.

## A40 Venom allergy testing: is a graded approach necessary?

### Jaclyn A. Quirt^1^, Xia Wen^2^, Jonathan Kim, Angel Jimenez Herrero, Harold L. Kim^1,3^

#### ^1^Division of Clinical Immunology and Allergy, Department of Medicine, McMaster University, Hamilton, ON, L8S 4L8, Canada, ^2^McGill University, Montreal, QC, H3A 0G4, Canada, ^3^Division of Clinical Immunology and Allergy, Department of Medicine, Western University, London, ON, N6A 4V2, Canada

##### **Correspondence:** Jaclyn A. Quirt - jaclyn.quirt@medportal.ca

*Allergy, Asthma and Clinical Immunology* 2016, **12(Suppl 1)**:A41

**Background:** Many institutions recommend a stepwise approach to intradermal testing for venom allergy [1]. This is costly and uncomfortable for the patient. The rational for this approach is the risk of potential adverse reactions to testing with the maximal dose alone. Strohmeier et al. [2] recently described the safety of a simultaneous approach to testing different venom concentrations, and Yocum has previously described the safety of a two-step approach to testing [3]. Our objective of this study was to evaluate the safety of a single step approach to venom allergy testing.

**Methods:** We retrospectively reviewed charts of 300 consecutive patients with suspected hymenoptera venom allergy based on history that underwent venom allergy testing in a single allergist’s clinic where a single step protocol had been adopted. Charts were reviewed for patient characteristics including medications, testing protocol used, results of testing, and reported immediate and delayed adverse reactions to testing.

**Results:** All patients underwent testing with an identical single-step protocol with an intradermal dose of 0.02 mL of a 1.0 μg/mL concentration of each of the five commercially available vespid and bee venoms. Patients ranged from 4 to 83 years of age. Only one patient reported an adverse reaction to testing, which was delayed until the morning following his visit. There were no immediate adverse reactions. This patient was successfully started on venom immunotherapy subsequent to his reaction.

**Conclusion:** A single step venom allergy intradermal testing protocol with a 1.0 μg/mL concentration of commercially available extracts is a safe option, which, if adopted into practice, would potentially lead to more streamlined care for patients and cost savings for the medical system.

**References**

1. Golden DBK, Moffitt J, Nicklas RA, Freeman T, Graft DF, Reisman RE et al. Stinging insect hypersensitivity: a practice parameter update 2011. J Allergy Clin Immunol. 2011;127:852–4.

2. Strohmeier B, Aberer W, Bokanovic D, Komericki P, Sturm GJ. Simultaneous intradermal testing with hymenoptera venoms is safe and more efficient than sequential testing. Allergy. 2013;68:542–4.

3. Yocum MW, Gosselin VA, Yunginger JW. Safety and efficacy of an accelerated method for venom skin testing. J Allergy Clin Immunol. 1996;97:1424–5.

## A41 The role of oral challenges in evaluating cephalosporin hypersensitivity reactions in children

### Magdalena J. Grzyb^1^, Marie-Noël Primeau^1^, Christine Lejtenyi^1^, Elaine Medoff^1^, Jennifer Mill^1^, Moshe Ben-Shoshan^1^

#### ^1^Pediatric Allergy and Clinical Immunology, Department of Pediatrics, McGill University, Montreal, QC, H4A 3J1, Canada

##### **Correspondence:** Magdalena J. Grzyb - magdalena.grzyb@mail.mcgill.ca

*Allergy, Asthma and Clinical Immunology* 2016, **12(Suppl 1)**:A42

**Background:** Characterizing reactions and assessing the safety of future cephalosporin administration in children is important, given the critical role these antibiotics play in the empirical treatment of serious infections. Because skin tests to cephalosporins remain to be validated [1], we aimed to evaluate the usefulness of oral challenges in assessing the risk of future reaction to these drugs.

**Methods:** Children presenting to drug allergy clinics at the Montreal Children’s Hospital during a consecutive 3-year period starting in March 2012, with a history of suspected hypersensitivity to cephalosporins, received graded oral challenges (10 %, followed by the remaining 90 % of the daily dose). We collected data on patient demographic and clinical characteristics using a standardized questionnaire and assessed the risk for immediate (within 1 h) and non-immediate reactions.

**Results:** Of the 56 children (52 % males) evaluated, 45 (80.4 %) had a suspected hypersensitivity to cefprozil [95 % CI 67.2, 89.3]. The rest of the study participants had a history of suspected reaction to cefixime (n = 4), cephalexin (n = 3), cefuroxime (n = 2), cefazolin (n = 1) or ceftriaxone (n = 1). Most of the initial reactions had been cutaneous, with 73.1 % of respondents reporting hives [95 % CI 59.5, 83.8], 16.1 % reporting maculopapular rash [95 % CI 8.1, 28.8] and 14.3 % angioedema [95 % CI 6.8, 26.8]. The majority of the reactions had occurred on first exposure to the drug and 1-3 days into the course, at 66.1 % [95 % CI 52.1, 77.8] and 63.3 % [95 % CI 49.5, 75.9], respectively. 32.1 % of the reactions developed 1-8 h after the most recent dose had been taken [95 % CI 20.6, 46.1] and 39 % developed more than 8 h after the dose [95 % CI 26.8, 79.2]. Following oral challenge, 2 participants developed immediate mild (cutaneous) reactions (3.6 %; 95 % CI 0.6, 13.4], both to cefprozil. Two participants developed non-immediate mild (cutaneous) reactions (one to cefprozil and the other to cephalexin).

**Conclusion:** The risk of reaction is low in children with a history of suspected hypersensitivity to cephalosporins. Oral challenges appear to be safe in this population, and may be the best tool available to diagnose cephalosporin hypersensitivity. Larger studies are needed to support these results.

**Reference**

1. Joint task force on practice parameters, American academy of allergy, asthma, and immunology: drug allergy: an updated practice parameter. Ann Allergy Asthma Immunol. 2010;105:259e273.

## A42 Breastfeeding and infant wheeze, atopy and atopic dermatitis: findings from the Canadian Healthy Infant Longitudinal Development Study

### Meghan B. Azad^1^, Zihang Lu^2^, Allan B. Becker^1^, Padmaja Subbarao^2^, Piushkumar J. Mandhane^3^, Stuart E. Turvey^4^, Malcolm R. Sears^5^, the CHILD Study Investigators^6^

#### ^1^Department of Pediatrics and Child Health, University of Manitoba and Children’s Hospital Research Institute of Manitoba, Winnipeg, MB, Canada, ^2^Department of Pediatrics, Hospital for Sick Children, University of Toronto, Toronto, ON, Canada, ^3^Department of Pediatrics, University of Alberta, Edmonton, AB, Canada, ^4^Department of Pediatrics, Child and Family Research Institute and British Columbia Children’s Hospital, University of British Columbia, Vancouver, BC, Canada, ^5^Department of Medicine, McMaster University, Hamilton, ON, Canada, ^6^Canadian Healthy Infant Longitudinal Development Study

##### **Correspondence:** Meghan B. Azad - meghan.azad@umanitoba.ca

*Allergy, Asthma and Clinical Immunology* 2016, **12(Suppl 1)**:A43

**Background:** The impact of breastfeeding on asthma and allergy development is controversial. We aimed to characterize the association of breastfeeding with childhood wheeze, atopy and atopic dermatitis in the first year of life.

**Methods:** We studied 2587 infants with complete breastfeeding data from the Canadian Healthy Infant Longitudinal Development (CHILD) birth cohort. Infant diet and wheezing episodes were reported by mothers at 3, 6, and 12 months. Atopy was determined by skin testing and atopic dermatitis was diagnosed at 12 months. Breastfeeding was classified as exclusive (human milk only), partial (supplemented with formula, other beverages or solid foods) or none.

**Results:** Breastfeeding rates were 82 % at 3 months (52 % exclusive, 30 % partial), 74 % at 6 months (13 % exclusive, 61 % partial), and 46 % at 12 months (partial). In their first year, 21 % of infants experienced ≥ 1 wheezing episode, 14 % were sensitized to ≥1 allergen, and 12 % were diagnosed with atopic dermatitis. Breastfeeding rates were lower among younger mothers, First Nations women, and those who smoked or did not have a postsecondary degree. Independent of these maternal characteristics, increasing duration and intensity of breastfeeding were associated with a reduced risk of wheezing: adjusted odds ratio (aOR) 0.97 (95 % CI 0.94–0.99) for each additional month of breastfeeding; aOR 0.68 (0.50–0.92) for exclusive versus no breastfeeding at 3 months. In contrast, breastfeeding was associated with an increased risk of atopy: aOR 1.04 (1.01–1.07) for each additional month; aOR 1.46 (1.02–2.10) for exclusive versus no breastfeeding. Breastfeeding was not associated with atopic dermatitis.

**Conclusions:** In the CHILD cohort, increasing duration and intensity of breastfeeding are associated with a lower risk of wheeze and a higher risk of atopy in the first year of life. Ongoing research will address potential confounding by reverse causation, extend analyses through early childhood, and evaluate physician-diagnosed asthma and food allergy.

## A43 *IL33* DNA methylation in bronchial epithelial cells is associated to asthma

### Anne-Marie Boucher-Lafleur^1^, Valérie Gagné-Ouellet^1^, Éric Jacques^2^, Sophie Plante^2^, Jamila Chakir^2^, Catherine Laprise^1^

#### ^1^Département des Sciences Fondamentales, Université du Québec à Chicoutimi, Chicoutimi, QC, Canada, ^2^Centre de recherche, Institut Universitaire de Cardiologie et de Pneumologie, Ste-Foy, QC, Canada

##### **Correspondence:** Anne-Marie Boucher-Lafleur - anne-marie.boucher-lafleur1@quac.ca

*Allergy, Asthma and Clinical Immunology* 2016, **12(Suppl 1)**:A44

**Background:** Inflammation is a key mechanism of asthma pathogenesis [1]. The bronchial epithelium acts as a barrier involved in innate and adaptive immunity through secretion of inflammatory mediators [2]. Interleukin (IL) 33 is an alarmin cytokine released by airway epithelial cells [3] that has been identified as a gene of susceptibility in asthma in several studies [4–6], as well as being a potential severity biomarker of asthma [7]. *IL33* gene expression was increased in epithelial cells from asthmatic individuals [8]. However, less is known about the epigenetic regulation of *IL33* particularly in severe asthma. In this context, we aimed to characterize DNA methylation (DNA-me) and expression signatures of *IL33* in epithelial cells obtained from mild and severe asthmatic individuals.

**Methods:** A total of 21 bronchial epithelial cell (BEC) lines from asthmatics individuals (mild n = 7; severe n = 4) and control individuals (n = 10) were used for DNA and mRNA extraction. DNA-me was measured by bis-pyrosequencing and mRNA level was assessed by qRT-PCR. Student t tests were performed to analyze the association between DNA-me and asthma severity (significant for Δβ > 5 % and p > 0.05). Pearson correlations were used to analyze the correlation between DNA-me and mRNA level.

**Results:** A hypomethylation for mild and severe BEC groups (Δβ = 22 %, p = 0.003; Δβ = 17 %, p = 0.090 respectively) was observed. The DNA-me of *IL33* promoter was also negatively correlated with mRNA levels (r = −0.750, p = 0.050), suggesting an upregulation of gene expression by DNA-me. The total mRNA level for *IL33* was increased in BECs from mild and severe asthmatic individuals (foldchange = 3.4, p = 0.017; foldchange = 4.7, p = 0.060 respectively).

**Conclusion:** Methylation modifications in *IL33* promoter could modulate gene expression in epithelial cells of asthmatic individuals. This study highlighted the relevance of *IL33* as a potential biomarker of asthma severity.

**References**

1. Calhoun WJ. The spectrum of asthma: an introduction. In: Brasier AR, editor. Heterogeneity in asthma. 1st ed. Galveston: Springer; 2014. p. 16–9.

2. Erle DJ, Sheppard D. The cell biology of asthma. J Cell Biol. 2014;205:621–31.

3. Cayrol C, Girard J. IL-33: an alarmin cytokine with crucial roles in innate immunity, inflammation and allergy. Curr Opin Immunol. 2014;31:31–7.

4. Moffat MF, Gut IG, Demenais F, Strachan, DP, Bouzignon, E, Heath S, von Mutius E, Farrall M, Lathrop M, Cookson WO. A large-scale, consortium-based genomewide association study of asthma. N Eng J Med. 2010;363:1211–21.

5. Torgeson DG, Ampleford EJ, Chiu GY, Gauderman WJ, Gignoux CR, Graves PE, Himes BE, Levin AM, Mathias RA, Hancock DB et al. Meta-analysis of genome-wide association studies of asthma in ethSnically diverse North American populations. Nat Genet. 2011;43:887–92.

6. Bønnelykke K, Sleiman P, Neilsen K, Kreiner-Moller E, Mercader JM, Belgrave D, den Dekker HT, Husby A, Sevelsted A, Faura-Tellez G et al. A genome-wide association study identifies CDHR3 as a susceptibility locus for early childhood asthma with severe exacerbation. Nat Genet. 2014;46:51–5.

7. Guo Z, Wu J, Zhao J, Liu F, Chen Y, Bi L, Liu S, Dong L. IL-33 promotes airway remodeling and is a marker of asthma disease severity. J Asthma. 2014;51:863–9.

8. Préfontaine A, Nadigel J, Chouiali F, Audusseau S, Semlali A, Chakir J, Martin JG, Hamid Q. Increased IL-33 expression by epithelial cells in bronchial asthma. J Allergy Clin Immunol. 2010;125:752–4.

## A44 NRF2 mediates the antioxidant response to organic dust-induced oxidative stress in bronchial epithelial cells

### Michael Chen^1^, Toby McGovern^1,2^, Mikael Adner^2^, James G. Martin^1^

#### ^1^Research Institute of the McGill University Health Centre, Department of Medicine, McGill University, Montréal, QC, Canada, ^2^Institute of Environmental Medicine, Karolinska Institutet, Stockholm, Sweden

##### **Correspondence:** Michael Chen - michael.chen5@mail.mcgill.ca

*Allergy, Asthma and Clinical Immunology* 2016, **12(Suppl 1)**:A45

**Background:** Bronchial epithelial cells play an important role in mediating the response to airway injury and repair through the release of chemotactic factors, such as interleukin 8 (IL-8) and antioxidant production. These antioxidant mechanisms are particularly important in the context of neutrophil-mediated pulmonary disease. Inhalation of organic dust (OD) from swine confinement facilities leads to pulmonary neutrophilia, airway hyperresponsiveness and oxidative stress. We sought to examine the role of NRF2, a critical transcription factor involved in mediating the endogenous antioxidant response, in bronchial epithelial cells following OD exposure. We hypothesized that OD exposure would increase NRF2 activity and antioxidant production in human bronchial epithelial cells.

**Methods:** A human bronchial epithelial cell line (BEAS-2B) was stimulated for 24 h with 100 µg OD. Supernatant was retained for evaluation of IL-8 by ELISA. qPCR was performed for NRF2-dependent and NRF2-independent antioxidant genes. NRF2 nuclear translocation was quantified at various time points using an NRF2 luciferase reporter assay and by immunofluorescence.

**Results:** OD exposure resulted in an increase of IL-8 release. mRNA expression levels of NRF2-dependent antioxidant genes HO-1, NQO1 and GCLM, but not of NRF2-independent antioxidant genes SOD 1, and catalase were increased following OD exposure compared to control cells. OD exposure induced a time and dose-dependent increase in luminescence indicating NRF2 nuclear translocation, a result confirmed by quantification of NRF2 nuclear localization by immunofluoresence.

**Conclusions:** OD exposure induces IL-8 release, NRF2 nuclear translocation, and upregulation of antioxidant gene expression in BEAS-2B cells. These results suggest a dual role for bronchial epithelial cells in mediating neutrophil recruitment as the endogenous antioxidant response. We demonstrated that the endogenous oxidative properties of OD are specific to NRF2 and that NRF2 is a critical mediator of OD-induced oxidative stress in bronchial epithelial cells.

## A45 The effects of perinatal distress, immune biomarkers and mother-infant interaction quality on childhood atopic dermatitis (rash) at 18 months

### Nela Cosic^1,2^, Henry Ntanda^1,2^, Gerald Giesbrecht^3^, Anita Kozyrskyj^4^, Nicole Letourneau^1,2^

#### ^1^Faculty of Nursing, Department of Pediatrics and Psychiatry, University of Calgary, Calgary, AB, Canada, ^2^Faculty of Medicine, Department of Pediatrics and Psychiatry, Cumming School of Medicine, University of Calgary, Calgary, AB, Canada, ^3^Faculty of Medicine, Department of Pediatrics, Cumming School of Medicine, University of Calgary, Calgary, AB, Canada, ^4^Faculty of Medicine and Dentistry, Department of Pediatrics, University of Alberta and School of Public Health, University of Alberta, Edmonton, AB, Canada

##### **Correspondence:** Nela Cosic - ncosic@ucalgary.ca

*Allergy, Asthma and Clinical Immunology* 2016, **12(Suppl 1)**:A46

**Background:** Perinatal psychosocial distress, including stressful life events, anxiety and depression are risk factors for childhood atopic dermatitis (rash) [1]. Maternal psychosocial distress is associated with excess prenatal stress hormones [2] and reduction in the quality of maternal-infant interaction [3], which may affect biomarkers of infant immunity such as interleukin (IL) levels and predispose the growing child to rash [4, 5]. This study will: (1) describe the association between perinatal distress and infant interleukins at 3 months of age, (2) describe the association of interleukins and atopic dermatitis (rash), and (3) build a best fit model from the identified associations in (1) and (2).

**Methods:** 120 women reported distress levels during pregnancy and at 3 months postpartum. Venous blood was collected from their 3-month-old infants to assess plasma interleukin levels. Maternal-child interaction was measured 6 months postpartum with the nursing child assessment teaching scale. Presence and number of skin areas affected by rash were assessed via parent report at 18 months. Correlation and multiple regression analyses identified the best fit model for rash using forward stepwise regression.

**Results:** Prenatal depression (r = −0.23, p = 0.01) and stressful life events pre- and post-natally (r = 0.27, p = 0.00) were associated with IL2p70. Pre- and post-natal anxiety were associated with IL8 (r = −0.28, p = 0.02) and IL4 (r = −0.24, p = 0.04). IL10 was associated with child skin rash (r = −0.25, p = 0.01) at 18 months. 20 % of the variance in childhood rash at 18 months was explained by the model (Table 11).

**Table 11:** Multiple regression analysis for variables predicting child skin rash

Variables	Estimate	Standard error	P value
Maternal age	−0.03	0.01	0.023
Maternal education	0.11	0.06	0.091
IL10	−1.31	0.41	0.002
Perinatal stress	−0.12	0.06	0.057
Maternal-infant interaction quality	0.02	0.01	0.018

*Note* IL10 was transformed by taking the square. * P < 0.05

**Conclusions:** Perinatal distress is associated with elevated infant ILs. Demographic variables (maternal age, education), IL10, perinatal stressful life events (e.g. separation/divorce, family death) and maternal-infant interaction best predicted skin rash in 18-month-old infants. We will now evaluate how maternal-child interaction moderates associations between both (1) perinatal distress and (2) immune biomarkers, and atopic dermatitis in children at 18 months.

**Acknowledgements:** Funding for this project was provided by the Canadian Institutes of Health Research, the Alberta Centre for Child, Community and Family Research, AllerGen NCE, and the University of Calgary Markin Studentship. Generous guidance in accurate formation of variables was provided by Dr. Allan Becker. We would also like to thank the participants of the Alberta Pregnancy Outcomes and Nutrition (APrON) Fetal Programming sub-study for their efforts and commitment to supporting this research.

**References**

1. Peters JL, Cohen S, Staudenmayer J, Hosen J, Platts Mills TA, Wright RJ. Prenatal negative life events increases cord blood IgE: interactions with dust mite allergen and maternal atopy. Allergy. 2012;67(4):545–51.

2. Giesbrecht G, Campbell T, Letourneau N, Kooistra L, Kaplan B, APrON Study Team. Psychological distress and salivary cortisol covary within persons during pregnancy. Pscyhoneuroendocrinology. 2011;27(12):171–80.

3. Letourneau N, Watson B, Duffett-Leger L, Hegadoren K, Tryphonopoulos P. Cortisol patterns of depressed mothers and their infants are related to maternal–infant interactive behaviours. J Reprod Infant Psychol. 2011;29(5):439–59.

4. Wright RJ. Prenatal maternal stress and early caregiving experiences: implications for childhood asthma risk. Paediatr Perinat Epidemiol. 2007, 21(s3):8–14.

5. Von Hertzen LC. Maternal stress and T-cell differentiation of the developing immune system: possible implications for the development of asthma and atopy. J Allergy Clin Immuno. 2002;109(6):923–8.

## A46 Examining the immunological mechanisms associated with cow’s milk allergy

### Bassel Dawod^1^, Jean Marshall^1,2^

#### ^1^Department of Pathology, Dalhousie University, Halifax, NS, Canada, ^2^Department of Microbiology and Immunology, Dalhousie University, Halifax, NS, Canada

##### **Correspondence:** Bassel Dawod - dawod@dal.ca

*Allergy, Asthma and Clinical Immunology* 2016, **12(Suppl 1)**:A47

**Background:** Oral tolerance is a state of unresponsiveness of the immune system to food antigens. Failure of tolerance can lead to future allergic reactions. Milk allergy, is the most common childhood allergy, affecting between 2–7 % of children [1]. Beta-lactoglobulin (BLG) is a major allergen in cow’s milk. Several factors, such as transforming growth factor-beta, vitamin A, and soluble toll-like receptors (TLRs) that are found in breast milk, are thought to enhance oral tolerance toward breastmilk-transferred antigens. In contrast, we have shown previously that PAM_3_CSK_4_, a TLR2 activator, is able to disrupt oral tolerance toward ovalbumin in mice. The variable levels of these tolerogenic or sensitizing factors in breast milk or baby formulas might be critical to the development of tolerance or allergy. The objective of this work was to better understand the impact of these milk factors in the development of tolerance toward milk-antigens.

**Methods:** Models of oral tolerance to cow’s milk, BLG, and ovalbumin were established. Oral tolerance was assessed in wild type and TLR2-deficient mice through analysis of antigen-specific antibody levels after a systemic antigen challenge. The development of antigen-specific Tregs was also assessed.

**Results:** Oral administration of doses of skim milk (above 1 mg/mL protein for 7 days) or BLG induced the development of oral tolerance, independently of TLR2. Immunoglobulin-E levels in antigen fed mice were 35 % of the control (n = 15). In contrast to ovalbumin, tolerance induction to BLG in milk was not altered by the addition of PAM_3_CSK_4_. Heat-killed *Lactobacilli* also did not modify milk tolerance. These results indicate that immunoregulatory factors in milk modify the response to an oral TLR2 activator.

**Conclusion:** This research could provide important insights into the significance of specific milk contents for the development of milk and other allergies, potentially informing both allergy prevention and treatment strategies.

**Acknowledgements:** Funded by AllerGen NCE and Canadian Institutes of Health Research (CIHR).

**Reference**

1. Bahna SL. Cow’s milk allergy versus cow milk intolerance. Ann Allergy Asthma Immunol. 2002;89(6): 56–60.

## A47 Tryptase levels in children presenting with anaphylaxis to the Montréal Children’s Hospital

### Sarah De Schryver^1^, Michelle Halbrich^2^, Ann Clarke^3^, Sebastian La Vieille^4^, Harley Eisman^5^, Reza Alizadehfar^1^, Lawrence Joseph^6^, Judy Morris^7^, Moshe Ben-Shoshan^1^

#### ^1^Division of Allergy and Clinical Immunology, Department of Pediatrics, Montréal Children’s Hospital, Montréal, QC, Canada, ^2^Division of Paediatric Allergy and Clinical Immunology, Department of Paediatrics, University of Manitoba, Winnipeg, MB, Canada, ^3^Division of Rheumatology, Department of Medicine, University of Calgary, Calgary, AB, Canada, ^4^Food Directorate, Health Canada, Ottawa, ON, Canada, ^5^Department of Emergency Medicine, Department of Pediatrics, Montréal Children’s Hospital, Montréal, QC, Canada, ^6^Department of Epidemiology and Biostatistics, McGill University, Montréal, QC, Canada, ^7^Department of Emergency Medicine, Hôpital du Sacré-Cœur, Montréal, QC, Canada

##### **Correspondence:** Sarah De Schryver - sarah.deschryver@yahoo.com

*Allergy, Asthma and Clinical Immunology* 2016, **12(Suppl 1)**:A48

**Background:** The study aimed to evaluate tryptase levels in children presenting with anaphylaxis, to examine predictors of elevated tryptase (defined as levels ≥11.4 ng/mL), and to compare tryptase levels during and post-anaphylaxis.

**Methods:** Between April 2011 and September 2014, data were collected on anaphylaxis cases at the Montreal Children’s Hospital Emergency Department. Cases were recruited either prospectively or identified retrospectively through chart review. Total tryptase levels were measured within 2 h following onset of symptoms. Levels during reaction and approximately 10 months after reaction were compared using confidence intervals based on paired means using the t distribution. Logistic and linear regression models were fit to estimate the associations between tryptase levels and sociodemographic and clinical characteristics of anaphylaxis.

**Results:** Over a three-year period, 165 children were recruited and had serum tryptase levels measured. Among those, the mean tryptase level was 7.6 μg/l (SD 6.4) and 32 cases [19.4 % (95 % CI, 13.8, 26.4 %)] had elevated tryptase levels. Elevated levels were found more frequently in severe reactions compared to moderate and mild reactions (Table 12).

**Table 12:** Elevated serum tryptase levels during reaction according to severity of reaction

Grades of anaphylaxis	Elevated tryptase (N)	Elevated tryptase %, 95 % CI
Overall	32/165	19.4 (13.8, 26.4)
Mild	6/32	18.8 (7.8, 37)
Moderate	19/121	15.7 (9.9, 32.7)
Severe	7/12	58.3 (28.6, 83.5)

Of the 54 children with post-reaction tryptase levels, the mean tryptase level was 8.2 μg/l during reaction and 3.7 μg/l post-reaction; yielding a difference of 3.5 (95 % CI 2.96, 3.96). Only severe reactions were associated with elevated tryptase levels (≥11.4 μg/l) [OR 7.2 (95 % CI 2.11, 24.40)]. Factors associated with an increase in tryptase levels regardless of previously published thresholds were severe reactions and milk trigger [beta = 10.3 (95 % CI 6.9, 13.7) and 4.3 (95 % CI 0.5, 8.0)], respectively.

**Conclusions:** Our study results do not support the role of tryptase as a reliable diagnostic biomarker for the diagnosis of anaphylaxis in children. Assessing the difference between levels during and post-reaction may improve the diagnostic utility of tryptase, mainly in severe reactions or reactions triggered by milk. Future studies are needed to evaluate more reliable diagnostic biomarkers.

**Acknowledgements:** This study was supported by the Allergy, Genes, and Environment (AllerGen) Network of Centres of Excellence (NCE) and Health Canada.

**Competing interests:** The authors have no conflicts of interest to declare.

## A48 Secondhand tobacco smoke exposure in infancy and the development of food hypersensitivity from childhood to adolescence

### Laura Y. Feldman^1,2^, Jesse D. Thacher^3,4^, Inger Kull^3,4^, Erik Melén^3,4^, Göran Pershagen^3^, Magnus Wickman^3,4^, Jennifer L. P. Protudjer^3,4,*^, Anna Bergström^3,4,*^

#### ^1^Dalla Lana School of Public Health, University of Toronto, Toronto, ON, Canada, ^2^Child Health Evaluative Sciences, The Hospital for Sick Children, Toronto, ON, Canada, ^3^Institute of Environmental Medicine, Karolinska Institutet, Stockholm, Sweden, ^4^Centre for Allergy Research, Karolinska Institutet, Stockholm, Sweden

##### **Correspondence:** Laura Y. Feldman - l.feldman@mail.utoronto.ca

*Allergy, Asthma and Clinical Immunology* 2016, **12(Suppl 1)**:A49

^*^ The authors contributed equally to this work

**Background:** Previous studies have demonstrated a link between early-life exposure to secondhand tobacco smoke (SHS) and allergen-specific immunoglobulin E (IgE) mediated sensitization to food allergens. However, it is unclear whether this association extends to clinical symptoms following food consumption [1]. We aimed to determine if SHS exposure during infancy is associated with food hypersensitivity from childhood to adolescence.

**Methods:** Data were obtained from the BAMSE birth cohort of 4089 Swedish children born in 1994–96 and followed to adolescence [2]. SHS exposure in infancy was assessed through parental report when the children were 2 months old. Food hypersensitivity was defined as the presence of parent-reported symptoms to specific food items at 1, 2, 4, 8, 12 and 16 years. Food sensitization was defined as an IgE ≥ 0.35 kU_A_/l to fx5^®^—a mix of milk, egg, soy, peanut, wheat and codfish allergens—at 4, 8 and 16 years.

Odds ratios (OR) and 95 % confidence intervals (95 % CI) from generalized estimating equations were used to calculate the overall association between SHS exposure in infancy and food hypersensitivity and/or food sensitization. Estimates were initially adjusted for young maternal age (≤25 years) at birth, exclusive breast feeding (≥4 months), parental allergy and socioeconomic status; they were subsequently adjusted for concomitant asthma.

**Results:** SHS exposure in infancy was associated with 1.12 times greater odds of reporting food hypersensitivity (OR 1.12; 95 % CI 0.96–1.30) and 1.29 times greater odds of food sensitization (OR 1.29; 95 % CI 1.06–1.57). With respect to concurrent outcomes, SHS exposure in infancy was associated with 1.43 times greater odds of having both food hypersensitivity and sensitization (OR 1.43; 95 % CI 1.06–1.87); this estimate did not change substantially upon adjustment for concomitant asthma (OR 1.39; 95 % CI 1.03–1.88).

**Conclusions:** SHS exposure in infancy is associated with food sensitization from childhood to adolescence, particularly with concurrent food hypersensitivity.

**Acknowledgements:** This work was supported by AllerGen NCE Inc. (the Allergy, Genes and Environment Network), a member of the Networks of Centres of Excellence Canada program.

**References**

1. Saulyte J, Regueira C, Montes-Martínez A, Khudyakov P, Takkouche B. Active or passive exposure to tobacco smoking and allergic rhinitis, allergic dermatitis, and food allergy in adults and children: a systematic review and meta-analysis. PLoS Med. 2014;11(3):e1001611.

2. Wickman M, Kull I, Pershagen G, Nordvall SL. The BAMSE project: presentation of a prospective longitudinal birth cohort study. Pediatr Allergy Immu. 2002;13(s15):11–3.

## A49 Combined exposure to diesel exhaust and allergen enhances allergic inflammation in the bronchial submucosa of atopic subjects

### Ali Hosseini^1,2^, Tillie L. Hackett^2^, Jeremy Hirota^1,2^, Kelly McNagny^3^, Susan Wilson^4^, Chris Carlsten^1,2^

#### ^1^Department of Medicine, University of British Columbia, Vancouver General Hospital, Vancouver, BC, Canada, ^2^University of British Columbia, Institute for Heart + Lung Health, Centre for Heart Lung Innovation, St. Paul’s Hospital, Vancouver, BC, Canada, ^3^University of British Columbia, Biomedical Research Centre, Vancouver, BC, Canada, ^4^Histochemistry Research Unit, University of Southampton, Southampton, Hampshire, UK

##### **Correspondence:** Ali Hosseini - ali.hosseini@alumni.ubc.ca

*Allergy, Asthma and Clinical Immunology* 2016, **12(Suppl 1)**:A50

**Background:** Asthma is a chronic inflammatory disease of the airways. Diesel exhaust (DE) is a major contributor to ambient particulate matter (PM). There is evidence that PM acts as an adjuvant to biological allergens and aggravates allergic inflammation [1, 2]. We aim to elucidate if DE increases allergen-induced inflammation and cellular immune response in the airways of atopic human subjects.

**Methods:** We recruited 12 volunteer subjects with allergy to house dust mite (HDM), birch or timothy grass. In a randomized blinded, crossover design, subjects were exposed to DE (300 µg PM_2.5_/m^3^) or filtered air for 2 h. One hour following the exposure, segmental allergen challenge was performed by instilling into contralateral lung segments through flexible bronchoscopy, either the allergen extract to which the participant is sensitive to, or placebo (sterile saline). Endobronchial biopsies from the same segments were then obtained 48 h post exposure. Thus, biopsies under four different conditions were acquired: filtered air and saline (FAS), DE and saline (DES), filtered air and allergen (FAA), and DE and allergen (DEA). Tissue biopsies were embedded in glycol methanlacrylate acrylic resin and 2 µm sections were cut and used for immunostaining with monoclonal antibodies to CD4, interleukin (IL)-4, tryptase and eosinophil cationic protein (ECP). Aperio ImageScope software was used to quantify the immunohistochemical staining of positive cells in the bronchial submucosa excluding smooth muscle and glands.

**Results:** The percent positivity for CD4 expression significantly increased from FAS (0.087 ± 0.018) to DEA (0.311 ± 0.06039; p = 0.035) in the bronchial submucosa. The percent positivity for IL-4 expression elevated from FAS (0.127 ± 0.062) to DEA (0.548 ± 0.143; p = 0.034). The percent positivity for tryptase and ECP expression remained unaffected. Data are presented as mean ± SEM.

**Conclusions:** This data suggest that co-exposure to DE and allergen augments CD4+ T cells recruitment and IL-4 expression, thus promoting Th2 polarization in the bronchial submucosa of atopic airways.

**Acknowledgements:** This study is funded by the Canadian Institutes of Health Research (CIHR) and AllerGen NCE Inc. A.H. is supported by the CIHR Transplantation Scholarship Training Program.

**References**

1. Riedl M, Diaz-Sanchez D. Biology of diesel exhaust effects on respiratory function. J Allergy Clin Immunol. 2005;115(2):221–8.

2. Nel AE, Diaz-Sanchez D, Ng D, Hiura T, Saxon A. Enhancement of allergic inflammation by the interaction between diesel exhaust particles and the immune system. J Allergy Clin Immunol. 1998;102(4):539–54.

## A50 Comparison of skin-prick test measurements by an automated system against the manual method

### Saiful Huq^1^, Rishma Chooniedass^1^, Brenda Gerwing^1^, Henry Huang^1^, Diana Lefebvre^2^, Allan Becker^1^

#### ^1^Department of Pediatrics and Child Health, University of Manitoba, Winnipeg, MB, Canada, ^2^Department of Medicine, McMaster University, Hamilton, ON, Canada

##### **Correspondence:** Saiful Huq - shuq@chrim.ca

*Allergy, Asthma and Clinical Immunology* 2016, **12(Suppl 1)**:A51

**Background:** Allergic diseases are the earliest of all chronic diseases to develop in children and their prevalence is on the rise [1, 2]. Skin prick tests (SPT) are considered an important diagnostic tool for allergies [3, 4]. SPT results to a specific allergen are typically calculated by manual measurement of the wheal of a response. The objective of this study is to evaluate a new, automated, image-processing method for wheal measurements against the standard of manual measurements.

The Canadian Healthy Infant Longitudinal Development (CHILD) Study is a general population, longitudinal study that focuses on the development of allergy in early life. For the purposes of this project, we measured SPTs from one-year clinic visits at the Manitoba CHILD site. SPTs were performed for common allergens as well as positive and negative controls, histamine and glycerine respectively.

**Methods:** SPTs were performed for 10 common allergens (cat, dog, cockroach, *D. farinae*, *D. pteronyssinus*, Alternaria, cow’s milk, egg white, soybean and peanut) and controls in 1033 children (mean age 13 ± 2 months). Of the 12,396 tests, there were measurable wheals for 1180 SPTs, identified by both automated and manual methods. By manual measurements 1105 wheals were ≥2 mm while by the automated method 1004 were ≥2 mm. Each child’s results were measured by hand and by an automated scan and measure system developed in our laboratory. The overall diameters averaged 2.94 ± 1.0 (range 0.5–7.8) by the automated method and 3.52 ± 1.1 (range 0.5–9.3) by the manual method with a correlation coefficient of 0.91.

**Results:** The results demonstrate the value of the automated measurements. The potential advantages of the automated method are reproducibility, accuracy, and speed when compared with manual measurements.

**References**

1. Boyce JA, Assa’ad A, Burks AW, et al. Guidelines for the diagnosis and management of food allergy in the United States: report of the NIAID-sponsored expert panel. J Allergy Clin Immunol. 2010;126(60):S1–58.

2. Eigenmann PA. Breaking frontiers for better early allergy diagnosis. Allergy. 2004;59(9): 895–6.

3. Celik-Bilgili S, Mehl A, Verstege A, Staden U, Nocon M, Beyer K, Niggemann B. The predictive value of specific immunoglobulin E levels in serum for the outcome of oral food challenges. Clin Exp Allergy. 2005;35(3): 268–73.

4. Kanny G, Moneret-Vautrin DA, Flabbee J, Beaudouin E, Morisse, M, Thevenin F. Population study of food allergy in France. J Allergy Clin Immunol. 2001;108(1):133–40.

## A51 The accurate identification and quantification of urinary biomarkers of asthma and COPD through the use of novel DIL-LC–MS/MS methods

### Mona M. Khamis^1^, Hanan Awad^1^, Kevin Allen^1^, Darryl J. Adamko^2^, Anas El-Aneed^1^

#### ^1^College of Pharmacy and Nutrition, University of Saskatchewan, Saskatoon, SK, Canada, ^2^College of Medicine, Department of Pediatrics, University of Saskatchewan, Saskatoon, SK, Canada

##### **Correspondence:** Mona M. Khamis - mmh882@mail.usask.ca

*Allergy, Asthma and Clinical Immunology* 2016, **12(Suppl 1)**:A52

**Background:** With the growing power of analytical capabilities, tens-to-hundreds of potential biomarkers are regularly being identified in discovery experiments. However, only a limited number has actually achieved FDA approval for routine clinical practices. One of the major impediment in biomarker discovery pipeline is the lack of coherent and well-established assays for subsequent validation steps [1].

Asthma and chronic obstructive pulmonary disease (COPD) are of particular importance to practitioners due to their overlapping clinical presentations. Diagnosis in regular outpatient clinics can be difficult. Current diagnostic tests are laborsome, unachievable in some cases, and/or insensitive to changes in airway function [2].

Metabolomics has demonstrated promising potential for discovering new biomarkers. Urine is an ideal matrix for metabolomic studies due to its richness in metabolites and ease of collection. A novel proton nuclear magnetic resonance (^1^H-NMR) study verified 50 polar urinary metabolites as candidate biomarkers for the differential diagnosis of asthma and COPD patients. Therefore, the next step in the ‘new biomarker’ pipeline is to accurately quantify and validate these metabolites with respect to their clinical sensitivity and specificity [2].

**Methods:** Two liquid chromatography-tandem mass spectrometry (LC–MS/MS) methods were developed to simultaneously quantify 34 metabolites in urine. Differential isotope labeling (DIL) was applied using ^12^C/^13^C-labeled dimethylaminophenacyl bromide and dansyl chloride reagents.

**Fig. 6:**
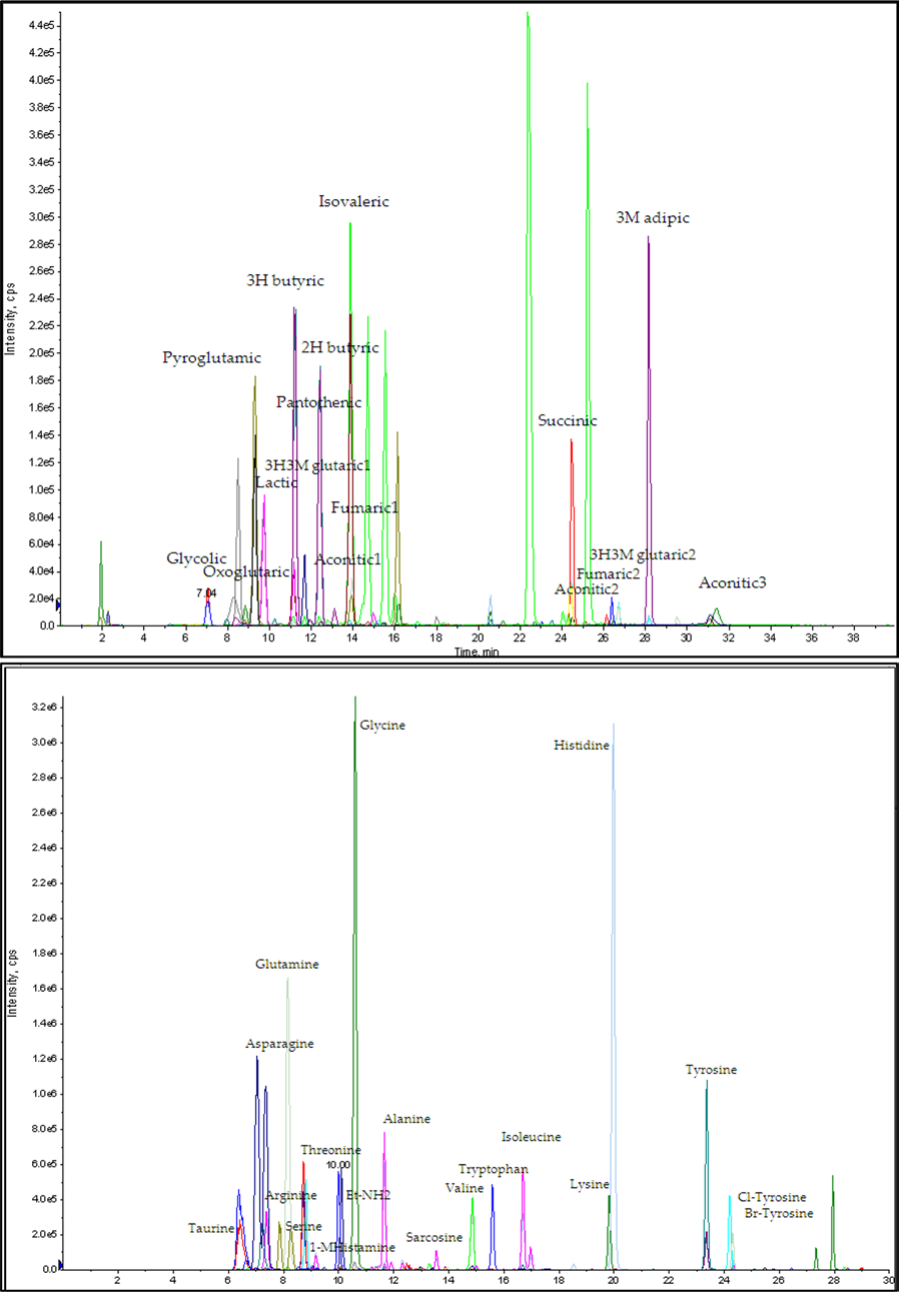
LC-MS/MS chromatogram of **a** ^12^C-dimethylaminophenacyl-labeled metabolites and corresponding ^13^C-internal standards and **b** ^12^C-dansyl-labeled metabolites and corresponding ^13^C-internal standards, in control urine

**Results:** DIL has significantly improved the detection of the polar metabolites in urine using a conventional LC–MS/MS platform. Methods were successfully developed to allow for the quantitative separation of the target metabolites. The use of structurally identical internal standards is an ideal option to correct for matrix effects from indigenous compounds within the urine (Fig. 6).

**Conclusion:** The developed methods are suitable for quantifying 34 candidate metabolites. Currently, we are validating the LC–MS/MS methods according to the FDA guidelines. This will allow us to quantify the targeted metabolites in the urine of asthma and COPD patients.

**References**

1. Beger RD, Colatsky T: Metabolomics data and the biomarker qualification process. Metabolomics. 2012;8:27.

2. Adamko DJ, Nair P, Mayers I, Tsuyuki RT, Regush S, Rowe BH. Metabolomic profiling of asthma and COPD: differentiating diseases. JACI, accepted manuscript. 2014.

## A52 Systemic immune pathways associated with the mechanism of Cat-Synthetic Peptide Immuno-Regulatory Epitopes, a novel immunotherapy, in whole blood of cat-allergic people

### Young Woong Kim^1,2,3^, Daniel R. Gliddon^4^, Casey P. Shannon^3^, Amrit Singh^1,2,3^, Pascal L. C. Hickey^5^, Anne K. Ellis^6,7^, Helen Neighbour^8,9^, Mark Larche^8,9^, Scott J. Tebbutt^1,2,3^

#### ^1^Experimental Medicine, University of British Columbia, Vancouver, BC, Canada, ^2^James Hogg Centre for Heart Lung Innovation, St. Paul’s Hospital, Vancouver, BC, Canada, ^3^Prevention of Organ Failure (PROOF) Centre of Excellence, Vancouver, BC, Canada, ^4^Circassia Ltd., Oxford, UK, ^5^Adiga Life Sciences, Hamilton, ON, Canada, ^6^Departments of Medicine and Biomedical and Molecular Science, Queen’s University, Kingston, ON, Canada, ^7^Allergy Research Unit, Kingston General Hospital, Kingston, ON, Canada, ^8^Department of Medicine, McMaster University, Hamilton, ON, Canada, ^9^Firestone Institute for Respiratory Health, McMaster University, Hamilton, ON, Canada

##### **Correspondence:** Young Woong Kim - YoungWoong.Kim@hli.ubc.ca

*Allergy, Asthma and Clinical Immunology* 2016, **12(Suppl 1)**:A53

**Background:** Allergic rhinitis (AR) is an IgE-mediated inflammatory condition of the nasal mucosa induced after allergen exposure [1]. Cat allergy affects 10–15 % of patients with allergic rhinitis and/or asthma [2]. A novel immunotherapy, Cat-Synthetic Peptide Immuno-Regulatory Epitopes (Cat-SPIRE), composed of seven synthetic peptide T-cell epitopes, acts on allergen-specific T-cells to induce subsequent clinical tolerance to cat allergen.

**Methods:** In this study, 19 participants with a clinical history of cat allergies with significant cat exposure received Cat-SPIRE (4 × 6 nmol intradermal injection, 1 dose every 4 weeks). Clinical symptoms were assessed by Nasal Allergen Challenge (NAC) [3]. Whole blood was collected into PAXgene tubes at baseline and post NAC (1, 2, and 6 h), before and 1 month post treatment. 770 immune genes in the PAXgene blood lysates at 6 h post NAC were profiled using the nanoString nCounter PanCancer Immune Profiling Panel. After normalization, a statistical comparison of pre-versus post-treatment changes was performed and an enrichment pathway analysis undertaken (Enrichr).

**Results:** 70 immune genes were significantly down regulated compared to pre-treatment at the 6 h post NAC time point (FDR < 10 %). These genes were associated with T-cell effector pathways, particularly canonical Th2 cytokines such as IL-4, IL-5, IL-3, IL-6 and TSLP. These changes were accompanied by significant post- versus pre-treatment reductions in clinical symptoms, Total Nasal Symptom Score and Peak Nasal Inspiratory Flow.

**Conclusion:** Following Cat-SPIRE treatment, systemic immune pathways of T-cell effectors were changed in peripheral blood of cat exposed, allergic individuals. The effect on these pathways provides insight towards the mechanism by which Cat-SPIRE induces immune tolerance. Whole blood immune transcriptome profiling in larger sample sizes and/or various time points after NAC may provide biomarkers or tools to discover the immune response or tolerance mechanism.

**References**

1. Pawankar R, et al. Allergic rhinitis and its impact on asthma in Asia Pacific and the ARIA update 2008. World Allergy Organ J. 2012;5(Suppl 3):S212–7.

2. Patel D, et al. Fel d 1–derived peptide antigen desensitization shows a persistent treatment effect 1 year after the start of dosing: a randomized, placebo-controlled study. J Allergy Clin Immunol. 2013;131(1):103–9.

3. Ellis AK, et al. The allergic rhinitis—clinical investigator collaborative (AR-CIC): nasal allergen challenge protocol optimization for studying AR pathophysiology and evaluating novel therapies. Allergy Asthma Clin Immunol. 2015l;11(1):16.

## A53 Reducing the health disparities: online support for children with asthma and allergies from low-income families

### Erika Ladouceur^1^, Miriam Stewart^1^, Josh Evans^2^, Jeff Masuda^3^, Nicole Letourneau^4^, Teresa To^5,6,7^, Malcolm King^8^

#### ^1^Faculty of Nursing, University of Alberta, Edmonton, AB, Canada, ^2^Faculty of Humanities and Social Sciences, Athabasca University, Athabasca, AB, Canada, ^3^School of Kinesiology and Health Studies, Queen’s University, Kingston, ON, Canada, ^4^Faculty of Nursing, University of Calgary, Calgary, AB, Canada, ^5^University of Toronto, Toronto, ON, Canada, ^6^Child Health Evaluative Sciences, The Hospital for Sick Children, Toronto, ON, Canada, ^7^Institute for Clinical Evaluative Sciences, North York, ON, Canada, ^8^Division of Pulmonary Medicine, University of Alberta, Edmonton, AB, Canada

##### **Correspondence:** Erika Ladouceur - eladouce@ualberta.ca

*Allergy, Asthma and Clinical Immunology* 2016, **12(Suppl 1)**:A54

**Background:** Children of low-income families access more urgent care and less preventive care for asthma [1]. Children with a respiratory disease from low-income families also experience a lower quality of life [2, 3]. Our previous online peer/professional support programs have improved children’s asthma and allergy self-management skills, and decreased loneliness [4–8]. However, in our previous programs, participants’ have come from high-income families [4–8]. Consequently, the objective of this research was to determine low-income participants’ perceptions of the impact of the support intervention, the factors influencing these impacts, their satisfaction with the intervention, and recommended changes.

**Methods:** Peer and professional mentors who lived with asthma and allergies and had experience with vulnerable families were recruited and trained to facilitate sessions. Participants in the cohort included 16 children aged 7–11, and adolescents 12–17 from across Canada. Children were provided with internet services for the program duration, chromebooks, and headsets/microphones that they could keep. Eight weekly meetings were offered on the Internet using GoToMeeting^®^; a secure online meeting platform. Quantitative data on health-related outcomes were elicited by standardized measures administered pre- and post-intervention. Qualitative data on the intervention processes was also collected.

**Results:** Preliminary analysis post-intervention, has shown an increase in coping skills and perceived support, and reduced loneliness after participating in the online sessions. Even more significant, in comparison to online programs with higher income families, was the increased understanding and appreciation of asthma/allergy triggers in participants. They also learned new ways to manage their triggers. Many parents reported that their children were less resistant to taking their medication and had a higher adherence to medication post-intervention due to an increased understanding about their medications and use.

**Conclusion:** It is evident that online-peer-support programs for lower income families require a different focus compared to programs developed for higher income families, as they face different challenges and have different needs.

**References**

1. To T, et al. Health outcomes in low-income children with current asthma in Canada. Chronic Dis Can. 2009;29:49–55.

2. Cesaroni G, Farchi S, Davoli M, et al. Individual and area-based indicators of socioeconomic status and childhood asthma. Eur Respir J. 2003;22:619–24.

3. Blais L, Beauchesne MF, Levesque, S. Socioeconomic status and medication prescription patterns in pediatric asthma in Canada. J Adolesc Health. 2006;38(5):e9–16.

4. Masuda JR, Anderson S, Letourneau N, Morgan VS, Stewart M. Reconciling preferences and constraints in building online peer support communities for youth with asthma and allergies. Health Promot Pract. 2013;14(5):741–50. doi:10.1177/1524839912465083.

5. Stewart M, Letourneau N, Masuda JR, Anderson S, McGhan SL. Impacts of online peer support for children with asthma and allergies: “It just helps you every time you can’t breathe well. J Pediatr Nurs. 2013;28:439–52.

6. Stewart M, Letourneau N, Masuda JR, Anderson S, McGhan SL. Online support for children with asthma and allergies. J Fam Nurs. 2013;19(2):171–97. doi: 10.1177/1074840713483573.

7. Letourneau N, Stewart M, Masuda JR, Anderson S, Cicutto L, McGhan SL, Watt S. Impact of online support for youth with asthma and allergies: Pilot study. J Pediatr Nurs. 2012;27(1):65–73.

8. Stewart M, Letourneau N, Masuda JR, Anderson S, McGhan SL. Online solutions to support needs and preferences of parents of children with asthma and allergies. J Fam Nurs. 2011;17(3):357–79. doi:10.1177/107484071141541.

## A54 Epigenetic association of *PSORS1C1* and asthma in the Saguenay-Lac-Saint-Jean asthma study

### Miriam Larouche^1^, Liming Liang^2^, Catherine Laprise^1^

#### ^1^Département des sciences fondamentales, Université du Québec à Chicoutimi, Chicoutimi, QC, Canada, ^2^Departments of Epidemiology and Biostatistics, Harvard T.H. Chan School of Public Health, Boston, MA, USA

##### **Correspondence:** Miriam Larouche - miriam.larouche@uqac.ca

*Allergy, Asthma and Clinical Immunology* 2016, **12(Suppl 1)**:A55

**Background:** Asthma is a chronic respiratory disease involving genetic and environmental interactions [1, 2]. More than 300 genes have been associated with asthma or related phenotypes, and the number continues to increase [3]. The associated common SNPs represent a small part of the genetic component and the exact nature of the causal genetic variants remains to be discovered [4]. One reason for this “missing heritability” is that many genetic factors act primarily through complex mechanisms involving interactions with other genes and environmental factors and/or epigenetic mechanisms [5]. We have shown that genetic variants related to asthma can be mechanistically studied using epigenetic and functional genomic assessment of individual variants [6, 7]. Therefore, we plan to focus on *IL33* pathway following the previous study demonstrating a correlation between methylation signature, expression and asthma in bronchial epithelial cells.

**Methods:** DNA methylation (Δβ) has been measured with an epigenome wide association study (EWAS) using Illumina HumanMethylation450K arrays among 69 asthmatic individuals and 91 controls. Data were extracted for 21,720 genes including 26 in the pathway of *IL33* and the association with asthma was assessed by logistic regression using methylation percentage, asthma phenotype, sex, age, IgE level, smoking status and eosinophils, lymphocytes, monocytes, neutrophils, and basophils counts as covariates. Batch effect was corrected for the analysis.

**Results:** A total of four genes have demonstrated a significant difference of methylation between asthmatic individuals and control ones: *CLSTN2* (Δβ = 7.35 %, p = 0.0008), *CDSN* (Δβ = 7.39 %, p = 0.02), *PSORS1C1* (Δβ = 7.39 %, p = 0.02) and *CCDC57* (Δβ = 5.63 %, p = 0.02).

**Conclusion:** This study shows differences in methylation marks between asthmatic individuals and non-asthmatic non-allergic controls for four genes (*CLSTN2*, *CDSN*, *PSORS1C1*, *CCDC57*). Interestingly, *PSORS1C1*, a susceptibility gene for psoriasis, has already been associated with asthma in a genome wide association study in Latino children [8]. The pyrosequencing of this gene will allow us to confirm this association.

**References**

1. Handoyo S, Rosenwasser LJ. Asthma phenotypes. Curr Allergy Asthma Rep. 2009;9(6): 439–45.

2. Harb H, Renz H. Update on epigenetics in allergic disease. J Allergy Clin Immunol. 2015;135(1):15–24.

3. Lee SH, Park JS, Park CS. The search for genetic variants and epigenetics related to asthma. Allergy Asthma Immunol Res. 2011;3(4): 236–44.

4. Laprise C, Bouzigon E. To define the biological nature of asthma. Curr Opin Allergy Clin Immunol. 2011;11(5):391–2.

5. Wjst M, Sargurupremraj M, Arnold M. Genome-wide association studies in asthma: what they really told us about pathogenesis. Curr Opin Allergy Clin Immunol. 2013;13(1):112–8.

6. Berlivet S, et al. Interaction between genetic and epigenetic variation defines gene expression patterns at the asthma-associated locus 17q12–q21 in lymphoblastoid cell lines. Human Genetics. 2012;131(7):1161–71.

7. Verlaan DJ, et al. Allele-specific chromatin remodeling in the ZPBP2/GSDMB/ORMDL3 locus associated with the risk of asthma and autoimmune disease. Am J Hum Genet. 2009;85(3): 377–93.

8. Galanter JM, et al. Genome-wide association study and admixture mapping identify different asthma associated loci in Latinos: the Genes-environments and Admixture in Latino Americans study. J Allergy Clin Immunol. 2014;134(2): 295–305.

## A55 IL-33 induces cytokine and chemokine production in human mast cells

### Stephanie A. Legere^1^, Ian D. Haidl^1^, Jean-Francois Legaré^1,2^, Jean S. Marshall^1^

#### ^1^Department of Microbiology and Immunology, Dalhousie University, Halifax, NS, Canada, ^2^Department of Surgery, Dalhousie University, Halifax, NS, Canada

##### **Correspondence:** Stephanie A. Legere - stephanie.legere@dal.ca

*Allergy, Asthma and Clinical Immunology* 2016, **12(Suppl 1)**:A56

**Background:** Sterile inflammation is a major mechanism by which tissue damage occurs throughout the body and is also a major determinant of disease progression. It is initiated after sterile physical damage has occurred in local tissues, but can also be initiated in a number of inflammatory disorders, such as asthma and arthritis. Many different cell types appear to be involved in sterile inflammation, but the role of mast cells has been of particular interest. Mast cells are innate immune sentinel cells that reside in many tissues throughout the body that synthesize and secrete many pro- and anti-inflammatory mediators. The alarmin IL-33 is an important component of the sterile inflammatory process and has been shown to directly activate populations of mast cells [1, 2]. Furthermore, IL-33 has been implicated as a potential early-life predictor of childhood asthma and allergy [3]. Therefore, the purpose of this study was to determine the cytokine and chemokine profile of human mast cells activated with IL-33.

**Methods:** Cord blood-derived mast cells (CBMCs) were activated for 24 h with IL-33 at a concentration of 30 ng/mL. To analyse protein production from CBMCs, supernatants were collected and a Luminex assay was conducted to analyse the concentration of 29 cytokines and chemokines. Additionally, gene expression data was analyzed through qPCR to determine gene expression changes upon activation of mast cells with IL-33.

**Results:** It was found that after IL-33 activation, CBMCs had a statistically significant increase in production of IL-13, but not other T_H_2-related cytokines such as IL-4. There was no increase in production of T_H_1-related cytokines, such as IFNγ and IL-12. Production of the cytokines VEGF and GM-CSF were also statistically significantly increased after IL-33 activation of CBMCs.

**Conclusions:** In conclusion, IL-33 activation of human mast cells results in the production of cytokines supportive of T_H_2-driven responses and tissue regeneration.

**References**

1. Allakhverdi Z, Smith DE, Comeau MR, Delespesse G. Cutting edge: the ST2 ligand IL-33 potently activates and drives maturation of human mast cells. J Immunol. 2007;179:2051–4.

2. Iiukura M, Suto H, Kajiwara N, Oboki K, Ohno T, Okayama Y, Saito H, Galli SJ, Nakae S. IL-33 can promote survival, adhesion and cytokine production in human mast cells. Lab Invest. 2007;87:971–8.

3. Ashley-Martin J, Dodds L, Arbuckle TE, Levy AR, Platt RW, Marshall JS. Predictors of interleukin-33 and thymic stromal lymphoprotein levels in cord blood. Pediatr Allergy Immunol. 2015;26:161–7.

## A56 Reference ranges for lung clearance index from infancy to adolescence for Canadian population

### Zihang Lu^1^, Malcolm Sears^2^, Theo J. Moraes^1^, Felix Ratjen^1^, Per Gustafsson^3^, Wendy Lou^4^, Padmaja Subbarao^1^

#### ^1^Division of Respiratory Medicine, Department of Pediatrics, and Program in Physiology and Experimental Medicine, SickKids Research Institute, The Hospital for Sick Children, and University of Toronto, Toronto, Canada, ^2^Department of Medicine, McMaster University, Hamilton, Canada, ^3^Department of Pediatrics, Central Hospital, Skövde, Sweden, ^4^Dalla Lana School of Public Health, University of Toronto, Toronto, Canada

##### **Correspondence:** Zihang Lu - zihang.lu@sickkids.ca

*Allergy, Asthma and Clinical Immunology* 2016, **12(Suppl 1)**:A57

**Background:** Lung clearance index (LCI), a parameter derived from the multiple breath washout (MBW) test, is a sensitive marker of ventilation inhomogeneity and has been associated with early lung disease. Appropriate representative normative reference data must be available to correctly interpret individual lung function results. We aim to develop a novel Canadian reference equation for LCI and compare it to previously published reference equations.

**Methods:** Two hundred and sixty-five subjects underwent MBW measurement using an AMIS mass spectrometer and 4 % SF6 as a tracer gas. All subjects were free of prior respiratory distress and had no history of wheezing or exposure to maternal smoking during pregnancy. For subjects under 4 years of age, data were obtained from 196 subjects recruited from the Canadian Healthy Infant Longitudinal Development (CHILD) Study. For subjects aged 4–15 years, data were obtained from 69 identified healthy controls. The lamda-mu-sigma (LMS) method was used to construct reference equation, with LCI modelling in terms of age and height.

**Results:** LCI was found to be independent of race and gender. Compared with reference equation from Lum et al. [1], predicted median LCI based on our reference equation was 0.41 [95 % confidence interval (CI) 0.39–0.43] lower. During infancy to 4 years of age, LCI decreased non-linearly as age and height increased (Table 13). After 4 years of age, LCI remained constant with a mean (standard deviation) of 6.15 (0.39), regardless of change in age and body size. This change in LCI values was associated with a decreased misclassification rate. In a group of well controlled asthmatics from our clinic, 25 % (9/36) subjects had an abnormal LCI when using our reference equation while only 11 % (4/36) were identified using the Lum et al. reference equation.

**Table 13:** Reference equation for lung clearance index (LCI) from infancy to 15 years of age

	Age ≤4	Age >4
Skewness (L)	−2.12	–
Predicted (M)	6.88–6.95 (*Age*/10)^2^ − 1.16 [(log *Length*)/10]^3^	6.15
Coefficient of variance (S)	0.07	–
Upper limited of normal (ULN)	*Predicted* × [(1.96 × 0.07 × (−2.12)) + 1]^1/−2.12^	6.91

**Conclusions:** Population-specific equations are necessary for the proper interpretation of clinical results.

**Reference**

1. Lum S, Stocks J, Stanojevic S, Wade A, Robinson Paul, Gustafsson P, Brown M, Aurora P, Subbarao P, Hooe A, Sonnappa S. Age and height dependence of lung clearance index and functional residual capacity. Eur Respir J. 2013;41:1371–7.

## A57 Kingston allergy birth cohort: cohort profile and mother/child characteristics to age 2

### Michelle L. North^1,2,3^, Elizabeth Lee^1^, Vanessa Omana^1^, Jenny Thiele^1,2,3^, Jeff Brook^4^, Anne K. Ellis^1,2,3^

#### ^1^Department of Biomedical and Molecular Sciences, Queen’s University, Kingston, ON, Canada, ^2^Division of Allergy and Immunology, Department of Medicine, Queen’s University, Kingston, ON, Canada, ^3^Allergy Research Unit, Kingston General Hospital, Kingston, ON, Canada, ^4^Air Quality Research Division, Environment Canada, Toronto, ON, Canada

##### **Correspondence:** Michelle L. North - northm@queensu.ca

*Allergy, Asthma and Clinical Immunology* 2016, **12(Suppl 1)**:A58

**Background:** The Kingston allergy birth cohort (KABC) was instigated to study the role of gene-environment interactions and epigenetic modifications in the developmental origins of allergy. Kingston General Hospital was chosen as the collection site, as it serves rural/urban residents with diverse socioeconomic status and has documented challenges such as a high rate of maternal smoking.

**Methods:** Pregnant women completed a health/environmental survey (n = 594). Umbilical cord blood was collected from 454 deliveries and environment/health surveys to age 2 encompassing 327 years of person-time from 235 children. We examined home-environment characteristics by urban/rural residence and socioeconomic status (SES; National Household Survey). Exposure to smoke was active/passive >3 days/week. For rates of parentally-reported skin, respiratory and food symptoms of potential allergic disease, only wheezing/coughing not associated with illness, and rashes that were not cradle cap, diaper rash or infectious were considered.

**Results:** Maternal and both-parent allergies were self-reported in 27.5, and 22.3 %. The KABC encompassed a high proportion of rural residents (41.3 %) and mothers exposed to smoke (25.9 %). Rural participants had significantly higher household income, less post-secondary education, more fireplace use, and more indoor dogs. Urban participants were significantly more likely to live near a major road. The homes of lowest-tertile-SES participants were significantly older, more likely to be apartment-style and to be located near a major road, relative to highest-tertile-SES. However, low-SES participants were significantly less likely to cook with gas, have a fireplace or an attached garage. The rate of development of parentally-reported skin, respiratory and food reactions in the KABC was 0.36, 0.17, and 0.17 cases/person-year, respectively.

**Conclusions:** We found that both residing in rural/urban and high/low-SES areas affected characteristics of the home and potential environmental exposures. Further analyses will explore rate differences between children with various exposures. Mother/child pairs are returning for skin testing and epigenetic analysis of umbilical cord and peripheral blood.

**Acknowledgements:** This abstract is presented on behalf of the KABC study group and epigenetic studies being carried out as a part of Rapid Environmental Effects on Genes: the Lens of Epigenetics (REEGLE). Investigators also include Michael Kobor and Miriam Diamond.

## A58 Cow’s milk protein specific IgE, IgA and IgG4 as a predictor of outcome in oral immunotherapy

### Tanvir Rahman^1^, Duncan Lejtenyi^2^, Sarah De Schryver^2^, Ryan Fiter^1^, Ciriaco Piccirillo^3^, Moshe Ben-Shoshan^2^, Bruce Mazer^1,2^

#### ^1^Meakins-Christie Laboratories, McGill University, Montréal, QC, Canada, ^2^Division of Pediatric Allergy and Clinical Immunology, Montréal Children’s Hospital, QC, Canada, ^3^Division of Allergy and Clinical Immunology, Montréal General Hospital, Montréal, QC, Canada

##### **Correspondence:** Tanvir Rahman - tanvir.rahman2@mail.mcgill.ca

*Allergy, Asthma and Clinical Immunology* 2016, **12(Suppl 1)**:A59

**Background:** Cow’s milk allergy (CMA) is defined as immunologic adverse reactions to cow’s milk proteins (CMP). It affects 2–3 % of infants [1]. Approximately 15 % of the CMA population retains their allergic status even after 8 years of age [2].

**Methods:** Twenty patients were recruited in a case control study of cow’s milk (CM) oral immunotherapy (OIT). Blood samples were collected at baseline and at multiple timepoints during the trial. Specific IgE, IgA and IgG4 to casein, β-lactoglobulin (BLG) and α-lactalbumin (ALA) were measured in OIT recipients (n = 9) by using Enzyme-Linked Immunosorbent Assays (ELISA).

**Fig. 7:**
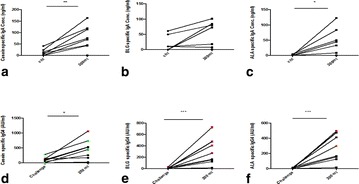
CMP-specific IgA to casein (**a**) and ALA (**c**) increased significantly from pre-therapy to 300 mL challenge with CM (unpaired t test, **P < 0.01 and *P < 0.05 respectively for casein and ALA). Casein (**d**), BLG (**e**), and ALA (**f**) specific IgG4 increased significantly baseline at the completion of OIT (unpaired *t* test, *p < 0.05, ***p < 0.001, ***p < 0.001 respectively for casein, BLG, and ALA)

**Results:** CMP-sIgE decreased multiple times baseline in 6 of 9 OIT recipients, remained high in 2 subjects, and unaltered in 1 individual due to OIT (data not shown). At the same time, specific IgA and specific IgG4 increased (Fig. 7) in parallel with the decrease in CMP-sIgE. Specific IgA increased significantly baseline in 7 of 9 patients in case of casein, and in 6 of 9 patients in case of BLG and ALA. Again, specific IgG4 to casein increased significantly baseline in 8 of 9 patients, and in all 9 patients in case of BLG and ALA.

**Conclusions:** In summary, a significant decrease in CMP-sIgE and a concomitant increase in CMP-sIgA and -IgG4 were observed in successful CM OIT recipients. These findings demand an extensive study of pro-inflammatory (e.g. T-helper cells) and anti-inflammatory cells (e.g. T- and B-regulatory cells) along with their secreted cytokines to elucidate the mechanism of the development of tolerance in CM OIT.

**References**

1. Saarinen KM, Juntunen-Backman K, Järvenpää AN, Kuitunen P, Lope L, Renlund M, Siivola M, Savilahti E. Supplementary feeding in maternity hospitals and the risk of cow’s milk allergy: a prospective study of 6209 infants. J Allergy Clin Immunol. 1999;104(2):457–61.

2. Saarinen KM, et al. Clinical course and prognosis of cow’s milk allergy are dependent on milk specific IgE status. J Allergy Clin Immunol. 2005;116(4):869–75.

## A59 Age of peanut introduction and development of reactions and sensitization to peanut

### Elinor Simons^1^, Allan B. Becker^1^, Rishma Chooniedass^1^, Kyla Hildebrand^2^, Edmond S. Chan^2^, Stuart Turvey^2^, Padmaja Subbarao^3^, Malcolm Sears^4^

#### ^1^Section of Allergy and Immunology, Department of Pediatrics and Child Health, University of Manitoba, Winnipeg, MB, Canada, ^2^Division of Allergy and Immunology, Department of Pediatrics, British Columbia Children’s Hospital, Vancouver, BC, Canada, ^3^Department of Pediatrics, Hospital for Sick Children, Toronto, ON, Canada, ^4^Division of Respirology, Department of Medicine, McMaster University, Hamilton, ON, Canada

##### **Correspondence:** Elinor Simons - elinor.simons@umanitoba.ca

*Allergy, Asthma and Clinical Immunology* 2016, **12(Suppl 1)**:A60

**Background:** Sensitization to peanut places a child at risk for anaphylaxis to peanut, although some peanut-sensitized children tolerate eating peanut. We evaluated the association between timing of peanut introduction in infancy and development of reactions and sensitization to peanut by age 3 years.

**Methods:** Caregivers of participants in the population-based Canadian Healthy Infant Longitudinal Development (CHILD) Study prospectively reported their child’s first introduction and reactions to peanut starting at age 6 months. At ages 1 and 3 years, the children underwent skin prick testing for sensitization to peanut and other food allergens, and an assessment for eczema/atopic dermatitis. We conducted multivariable logistic regression to determine if later introduction of peanut was associated with increased odds of reactions and sensitization to peanut.

**Results:** Among participants from the Manitoba and Vancouver sites (n = 1610), peanut was introduced into the diet at ages 6 (1.3 %), 9 (14.1 %), 12 (27.4 %) and >12 (42.3 %) months; 14.9 % had not tried peanut by 18 months. At age 1 year, 2.4 % of children had moderate-to-severe eczema. By age 3 years, 5.6 % of children had a reaction to peanut and 7.2 % had at least one positive skin prick test to peanut, 3.4 % to milk, and 9.3 % to egg. In the first 3 years, 53.9 % of peanut-sensitized children had reacted to peanut and 77.8 % of children who had reacted to peanut were peanut-sensitized. Children with later peanut introduction (on a continuous scale from 6–18 months) had higher odds of a reaction (1.04; 95 % CI 1.02–1.06) and sensitization (1.04; 1.03–1.05) to peanut, after adjustment for sensitization to egg and moderate-to-severe eczema.

**Conclusion:** In a population-based sample of Canadian children, later peanut introduction was associated with reported reactions and sensitization to peanut by age 3 years. Further investigation is needed to characterize the association between timing of peanut introduction and persistent peanut allergy.

## A60 Multi-omic blood biomarker signatures of the late phase asthmatic response

### Amrit Singh^1^, Casey P. Shannon^1^, Young Woong Kim^1^, Mari DeMarco^1^, Kim-Anh Le Cao^2^, Gail M. Gauvreau^3^, J. Mark FitzGerald^1^, Louis-Philippe Boulet^4^, Paul M. O’Byrne^3^, Scott J. Tebbutt^1^

#### ^1^Department of Medicine, University of British Columbia, Vancouver, BC, Canada, ^2^Translational Research Institute, University of Queensland, Brisbane, Australia, ^3^Department of Medicine, McMaster University, Hamilton, ON, Canada, ^4^Centre de Pneumologie de L’Hopital, Université Laval, Québec City, QC, Canada

##### **Correspondence:** Amrit Singh - Amrit.Singh@hli.ubc.ca

*Allergy, Asthma and Clinical Immunology* 2016, **12(Suppl 1)**:A61

**Background:** Individuals with allergic asthma respond differently, but reproducibly, to allergen inhalation challenge. Some individuals develop an isolated early response (early responders, ERs) while others also go on to develop a late response (dual responders, DRs). Since new drugs for allergic asthma are targeted towards attenuating the late phase response, the purpose of this study is to identify a multi-omic biomarker panel that can screen mild asthmatics for those likely to exhibit the late response.

**Methods:** 32 individuals participated in the allergen inhalation challenge as part of the AllerGen clinical investigator collaborative. 15 (17) participants were classified as ERs (DRs), respectively. Blood samples were collected prior to the allergen inhalation challenge. Measurements of cell counts were obtained using a hematolyzer; gene transcript relative levels using RNA sequencing; metabolite levels using tandem mass spectrometry. Sparse generalized canonical correlation discriminant analysis was used to classify ERs and DRs by integrating all three datasets, adjusting for age, sex and allergen. Geneset enrichment analysis was performed using Enrichr.

**Results:** After pre-processing and filtering, 5 cell-types, 11,879 gene transcripts and 124 metabolites were retained for downstream analysis. The multi-signature classifier consisted of 2 cell-types, 10 gene transcripts and 10 metabolites and had an error rate of 20 ± 8.3 % (10 × 5-fold cross-validation). The cells selected in the multi-signature panel included lymphocytes and monocytes. The selected metabolites were enriched for phosphatidylcholines. The top 10 enriched pathways in the selected gene transcripts included the phosphatidylinositol signalling system.

**Conclusions:** Molecular signatures in blood can be used to screen for asthmatic individuals that develop the late asthmatic response. This study implicates the arachidonic acid metabolism pathway in leading to the early or late phase asthmatic response.

## A61 Early life gut microbial alterations in children diagnosed with asthma by three years of age

### Leah T. Stiemsma^1,2^, Marie-Claire Arrieta^1,3^, Jasmine Cheng^1,2^, Pedro A. Dimitriu^1^, Lisa Thorson^3^, Sophie Yurist^3^, Boris Kuzeljevic^2^, Diana L. Lefebvre^4,5^, Padmaja Subbarao^6,7^, Piush Mandhane^8,9^, Allan Becker^10^, Malcolm R. Sears^5^, Kelly M. McNagny^11^, Tobias Kollmann^2, 12^, the CHILD Study Investigators, William W. Mohn^1^, B. Brett Finlay^1,3,13^, Stuart E. Turvey^2,12^

#### ^1^Department of Microbiology and Immunology, University of British Columbia, Vancouver, BC, Canada, ^2^The Child and Family Research Institute, Vancouver, BC, Canada, ^3^Michael Smith Laboratories, University of British Columbia, Vancouver, BC, Canada, ^4^St. Joseph’s Healthcare, Hamilton, ON, Canada, ^5^Department of Medicine, McMaster University, Hamilton, ON, Canada, ^6^Department of Pediatrics, University of Toronto, Toronto, ON, Canada, ^7^Hospital for Sick Children, Toronto, ON, Canada, ^8^Department of Pediatrics, University of Alberta, Edmonton, AB, Canada, ^9^School of Public Health, University of Alberta, Edmonton, AB, Canada, ^10^Department of Pediatrics and Child Health, University of Manitoba, Winnipeg, MB, Canada, ^11^The Biomedical Research Centre, University of British Columbia, Vancouver, BC, Canada, ^12^Department of Pediatrics, University of British Columbia, Vancouver, BC, Canada, ^13^Department of Biochemistry and Molecular Biology, University of British Columbia, Vancouver, BC, Canada

##### **Correspondence:** Leah T. Stiemsma - lthomas@cfri.ca

*Allergy, Asthma and Clinical Immunology* 2016, **12(Suppl 1)**:A62

**Background:** Asthma is the most prevalent childhood disease affecting over 300 million people worldwide. Our research group previously associated features of the early life gut microbiota (at 3 months and 1 year of age) in children with risk of active asthma at school age (determined by the asthma predictive index). These features included underrepresentation of four bacterial genera and a decreased production of microbial derived metabolites. We hypothesize that this constitutes a dysbiotic early life microbiota, which is also associated with asthma diagnosed in children by 3 years of age and sought to identify additional bacterial taxa that may be associated with this asthmatic phenotype.

**Methods:** 287 children enrolled in the Canadian Healthy Infant Longitudinal Development (CHILD) Study were classified according to the diagnosis of asthma by 3 years of age. Bacterial 16S rDNA from 3-month and 1-year stool samples from these children was extracted, amplified, and subjected to high throughput Illumina sequencing. Specific bacterial genera and species were quantified by quantitative PCR from all children with asthma and a subset of controls with no history of asthma symptoms. An exact logistic regression model was used to assess the effects of potentially confounding variables (i.e. antibiotic exposure, mode of birth).

**Results:** 16S sequence analysis of our sample cohort identified differentially abundant bacterial populations between asthmatics and controls in stool collected at 3 months and 1 year of age. Additionally, qPCR identified significant shifts in the abundance of specific bacterial genera in stool collected at 3 months and 1 year of age in the asthmatic group when compared to controls.

**Conclusions:** Shifts in the relative abundance of specific gut bacterial populations in early life are associated with asthma diagnosed in children by 3 years of age.

## A62 The relationship between food sensitization and atopic dermatitis at age 1 year in a Canadian birth cohort

### Maxwell M. Tran^1^, Diana L. Lefebvre^1^, Chinthanie F. Ramasundarahettige^1^, Allan B. Becker^2^, Wei Hao Dai^3^, Padmaja Subbarao^3^, Piush J. Mandhane^4^, Stuart E. Turvey^5^, Malcolm R. Sears^1^

#### ^1^Department of Medicine, McMaster University, Hamilton, ON, Canada, ^2^Department of Pediatrics and Child Health, University of Manitoba, Winnipeg, MB, Canada, ^3^Department of Paediatrics, University of Toronto, Toronto, ON, Canada, ^4^Department of Pediatrics, University of Alberta, Edmonton, AB, Canada, ^5^Department of Pediatrics, University of British Columbia, Vancouver, BC, Canada

##### **Correspondence:** Maxwell M. Tran - tranmm@mcmaster.ca

*Allergy, Asthma and Clinical Immunology* 2016, **12(Suppl 1)**:A63

**Background:** The relationship between immunoglobulin E (IgE)-mediated food sensitization and the development of atopic dermatitis during infancy is not well-characterized. Past studies have shown that food sensitization is associated with the occurrence of atopic diseases [1–3], including atopic dermatitis, although this relationship has not been examined in a population-based birth cohort at 1 year. Our aim was to address this knowledge gap by analyzing results from nearly 3000 children enrolled in the Canadian Healthy Infant Longitudinal Development (CHILD) Study.

**Methods:** An algorithm based on Child Health questionnaires completed by parents when their children were 1 year old was used to classify children as having definite, possible, or no atopic dermatitis. Atopic dermatitis classification was cross-tabulated with skin prick test (SPT) results to food allergens, specifically peanut, milk, egg, and soybean. A positive SPT was defined by a wheal diameter of ≥2 mm compared to the negative control. In total, 2626 children who underwent skin testing and had corresponding questionnaire data were included in this project.

**Results:** Of 2626 children, 526 (20.0 %) had definite atopic dermatitis and 906 (34.5 %) had possible atopic dermatitis (Table 14). There was an increasing trend in the proportion of positive SPTs with increasing certainty of atopic dermatitis (Cochran-Armitage trend test, p < 0.001). Sensitization to any food allergen was associated with higher odds of having definite or possible atopic dermatitis (OR 2.04, 95 % CI 1.64–2.53). Specifically, sensitization to peanut (p < 0.0001), egg (p < 0.0001), and milk (p = 0.0021) were significantly associated with atopic dermatitis, but sensitization to soybean (p = 0.3470) was not.

**Table 14:** Cross-tabulation of food sensitization and atopic dermatitis classification

SPT results	Atopic dermatitis classification			
	Definite	Possible	No	Total
Sensitized to ≥1 food allergen(s)	112 (21.3 %)	98 (10.8 %)	109 (9.1 %)	319 (12.1 %)
Non-sensitized	414 (78.7 %)	808 (89.2 %)	1085 (90.9 %)	2307 (87.9 %)
Total	526	906	1194	2626

**Conclusions:** IgE-mediated sensitization to any food allergen at 1 year was associated with higher odds of having definite or possible atopic dermatitis. Sensitization to peanut, egg white, and milk, but not soybean, were significantly associated with atopic dermatitis.

**Acknowledgements:** The authors are grateful to the families who participated in this study, the CHILD Study team, and the Allergy, Genes and Environment (AllerGen) Network of Centres of Excellence.

**References**

1. Chiu CY, Huang YL, Tsai MH, Tu YL, Hua MC, Yao TC, et al. Sensitization to food and inhalant allergens in relation to atopic diseases in early childhood: a birth cohort study. PLoS One. 2014;9(7):e102809.

2. Hill DJ, Sporik R, Thorburn J, Hosking CS. The association of atopic dermatitis in infancy with immunoglobulin E food sensitization. J Pediatr. 2000;137(4):475–9.

3. Eller E, Kjaer HF, Høst A, Andersen KE, Bindslev-Jensen C. Development of atopic dermatitis in the DARC birth cohort. Pediatr Allergy Immunol. 2010;21:307–14.

## A63 Allergen inhalation enhances toll-like receptor-induced thymic stromal lymphopoietin receptor expression by hematopoietic progenitor cells in mild asthmatics

### Damian Tworek^1^, Delia Heroux^1^, Seamus N. O’Byrne^1^, Paul M. O’Byrne^1^, Judah A. Denburg^1^

#### ^1^Department of Medicine, McMaster University, Hamilton, ON, Canada

##### **Correspondence:** Damian Tworek - damian.tworek@gmail.com

*Allergy, Asthma and Clinical Immunology* 2016, **12(Suppl 1)**:A64

**Background:** Hematopoietic progenitor cells (HPCs) may act as pro-inflammatory effector cells by themselves and directly contribute to allergic inflammation. HPCs express the receptor for thymic stromal lymphopoietin (TSLPR) and respond TSLP by rapidly releasing high levels of pro-inflammatory Th2-like cytokines and chemokines. HPCs also express toll-like receptors (TLRs)-2, -4 and -9, and TLR ligation induces eosinophil-basophil differentiation from HPCs. In the current study we investigated the effects of allergen inhalation on TLR-induced TSLPR expression by HPCs.

**Methods:** Seven mild allergic asthmatics underwent bronchial allergen challenges. All subjects developed a dual response to inhaled allergen. Blood was collected before and 24 h after the challenge. CD34-enriched peripheral blood (pb) cell populations were stimulated with TLR-2 (lipoteichoic acid, LTA), TLR-4 (lipopolysaccharide, LPS) or TLR-9 (ODN2006) ligands. TLR expression by pb HPCs as well as TSLPR expression by HPCs stimulated with TLR agonists pre- and post-challenge were measured by flow cytometry.

**Results:** There was no significant change in TLR-2, TLR-4 or TLR-9 expression post allergen inhalation. TSLPR was barely detected on unstimulated HPCs. However, overnight stimulation of HPCs with LPS and ODN2006 induced a significant increase in TSLPR expression compared with unstimulated HPCs (p < 0.01 and p < 0.05, respectively). In addition, LPS- and ODN2006-induced TSLPR expression by HPCs was more pronounced post-allergen compared to baseline (p < 0.001 and p < 0.05, respectively). No significant effect of LTA on TSLPR expression was noted.

**Conclusions:** Allergen exposure changes the response of HPCs to TLR stimulation by enhancing TSLP (and thus pro-inflammatory) responsiveness. These findings suggest that HPCs may be relevant in the pathogenesis of pathogen-related asthma exacerbations.

## A64 The allergic rhinitis clinical investigator collaborative—replicated eosinophilia on repeated cumulative allergen challenges in nasal lavage samples

### Laura Walsh^1^, Mena Soliman^1,2^, Jenny Thiele^1,2^, Lisa M. Steacy^2^, Daniel E. Adams^2^, Anne K. Ellis^1,2^

#### ^1^Departments of Medicine and Biomedical and Molecular Science, Queen’s University, Kingston, ON, Canada, ^2^Allergy Research Unit, Kingston General Hospital, Kingston, ON, Canada

##### **Correspondence:** Laura Walsh - 12lw1@queensu.ca

*Allergy, Asthma and Clinical Immunology* 2016, **12(Suppl 1)**:A65

**Background:** The allergic rhinitis clinical investigator collaborative (AR-CIC) is a national initiative conducting standardized nasal allergen challenges (NAC) to investigate the effectiveness of therapeutic agents for allergic rhinitis (AR), as well as analyze biomarkers and potential pathways of the allergic response. The NAC involves direct exposure of the nasal mucosa to the allergen of interest and evaluation of clinical, cellular and molecular outcomes.

**Methods:** 10 ragweed allergic participants, with a history of AR and a skin prick test to short ragweed ≥3 mm than the negative control, were enrolled. Participants were screened for inclusion/exclusion criteria and their qualifying allergen concentration determined. Participants returned 21–28 days later and received the cumulative allergen concentration on two NAC visits separated by 21 days. Nasal lavage samples were collected at baseline, 1, 6, and 24 h after allergen challenge. The samples were kept on ice until cytospin slides were prepared and stained with Diff-Quik™. Differential cell counts were completed for each sample to determine the eosinophil fraction as a percent of the total white blood cells. GraphPad Prism™ was used for all statistical analysis.

**Results:** Starting at 1 h, there was an increase in eosinophils after NAC which was significant at 24 h at both NAC visits (p ≤ 0.05). When compared to non-allergic controls, obtained previously under similar study conditions, a significant increase in the eosinophil population of allergic participants was observed for both NAC visits at 1 and 6 h post NAC time points (p ≤ 0.05). 24-hour samples for non-allergics were not collected in the previous study. No significant differences were identified between the consecutive NAC visits concerning eosinophil fraction in allergics at any time point.

**Conclusions:** There were no significant differences in eosinophil fraction between the two NAC visits, indicating repeatability of nasal eosinophilia using the repeated cumulative allergen challenges (RCAC) protocol.

## A65 The CHILD Study: optimizing subject retention in pediatric longitudinal cohort research

### Linda Warner^1^, Mary Ann Mauro^1^, Robby Mamonluk^1^, Stuart E. Turvey^1^

#### ^1^Department of Pediatrics, University of British Columbia, Vancouver, BC, Canada

##### **Correspondence:** Linda Warner - lwarner@cfri.ca

*Allergy, Asthma and Clinical Immunology* 2016, **12(Suppl 1)**:A66

**Background:** The Canadian Healthy Infant Longitudinal Development (CHILD) Study is a multicentre birth cohort study following children for at least the first 5 years of life to determine how environmental and genetic variables impact health, and particularly the development of asthma, eczema, and allergies. Subject retention is essential for optimal scientific integrity.

**Methods:** Vancouver-based participants completed surveys after 1 and 5 years. The 1-year surveys focused on what was important to families for their continued participation and reasons for potential withdrawal (reported at the 2013 Canadian Society of Allergy and Clinical Immunology Scientific Meeting). The 5-year surveys reassessed reasons for ongoing participation and evaluated retention strategies.

**Results:** The first 79 subjects, due for the 5-year clinic visit, were analyzed through April 2015: 69/79 (87 %) completed the visit, 1/79 (1 %) transferred to the Toronto site, 3/79 (4 %) booked future visits, and efforts to schedule the remaining 5/79 (6 %) are ongoing. Importantly, no participants have been lost to follow-up at this stage, including 15 families who moved outside of Vancouver. Of the 69 visits completed: 57/68 (84 %) of blood samples were collected, including only 2 refusals and 10 failures related to lab closure; 68/69 (99 %) of urine samples were collected; 69/69 (100 %) skin prick tests were completed; and 68/69 (99 %) spirometry assessments were completed.

From survey data, parents identified their highest priority for study participation as: desire to improve child health globally (68 %), a positive experience for the child (19 %), ease of online questionnaires (6 %), monetary reimbursement (4 %) and flexibility in scheduling (2 %).

**Conclusion:** Successfully engaging participants is crucial to achieving study objectives, particularly in a pediatric longitudinal study where there is a high likelihood for change over time. In our Vancouver experience, ongoing engagement with families through surveys has ensured that challenges are identified and addressed during the course of the study. Ultimately, active participant engagement in the CHILD Study likely enhanced subject retention and protocol completion to optimize scientific integrity.

## A66 Differential expression of C3a and C5a in allergic asthma

### ChenXi Yang^1,2,3^, Amrit Singh^1,2^, Casey P. Shannon^1,2^, Young Woong Kim^1,2^, Ed M. Conway^3^, Scott J. Tebbutt^1,2^

#### ^1^Centre for Heart and Lung Innovation, University of British Columbia, Vancouver, BC, Canada, ^2^Prevention of Organ Failure (PROOF) Centre of Excellence, Vancouver, BC, Canada, ^3^Centre for Blood Research, University of British Columbia, Vancouver, BC, Canada

##### **Correspondence:** ChenXi Yang - y.yolanda0507@gmail.com

*Allergy, Asthma and Clinical Immunology* 2016, **12(Suppl 1)**:A67

**Background:** Allergic asthma is an airway inflammatory disease characterized by airway obstruction, increasing bronchovascular permeability, and airway hyperresponsiveness. Two phenotypes—early responders (ER), who only experience an acute bronchoconstriction within minutes following the allergen exposure, and dual responders (DR), who also experience a further longer-lasting bronchoconstriction several hours after the initial exposure—are involved in the disease, but the mechanism that leads to the two phenotypes remains elusive. Activation of the complement system, which is part of innate immunity, is strongly associated with the disease. Various models have shown that the complement anaphylatoxins, C3a and C5a, play an important role in regulating the allergic response owing to their pro-inflammatory effects [1]. Therefore, we hypothesized that C3a and C5a are differentially abundant in plasma of ERs, DRs and non-asthmatic controls.

**Methods:** 14 mild asthmatic and 6 non-asthmatic control individuals participated in our study. Peripheral whole blood samples were collected using EDTA tubes and the samples were further processed to obtain plasma. Quidel MicroVue™ C3a Plus and C5a EIA kits were used to measure C3a and C5a expression levels, respectively. Student’s t-tests were then used to compare C3a and C5a levels in plasma of ERs, DRs and non-asthmatic controls.

**Results:** C3a levels were lower in controls compared to ERs (p < 0.01) and DRs (p < 0.05) but there was no significant difference between ERs and DRs. C5a levels were up-regulated in ERs compared to DRs (p < 0.01) and controls (p < 0.05). The down-regulation of C5a in DRs may be due to the fact that C5a is protective during allergen sensitization.

**Conclusions:** Both C3a and C5a expression levels are associated with allergic asthma. C3a is lower in non-asthmatics compared to ERs and DRs; C5a is higher in ERs compared to DRs and non-asthmatics. Further validation is ongoing.

**Reference**

1. Zhang X, Kohl J. A complex role of complement in allergic asthma. Expert Rev Clin Immunol. 2010;6(2):269–77.

